# Supplementary material for: Implementation barriers and facilitators of remote monitoring, remote consultation and digital care platforms through the eyes of healthcare professionals: a review of reviews
Source: BMJ Open. 2024 Jun 10;14(6):e075833. doi: 10.1136/bmjopen-2023-075833 (PMC11168143; doi:10.1136/bmjopen-2023-075833)
Supplement: Supplementary data [file bmjopen-2023-075833supp001.pdf]

Supplementary material

Supplementary Table 1. In- and exclusion criteria

Inclusion criteria	Exclusion criteria
• Publication type: systematic review, meta-analysis, literature review. At least when systematic review was mentioned in title or abstract.<br>• Describing barriers, facilitators, successes, inhibitions, failures for the adoption, implementation, maintenance of technology in practice.<br>• From the healthcare professional perspective: medical specialists, nurse practitioners, and physician assistants.	• Articles written in other language than Dutch, German, or English<br>• Publication type: research protocol, empirical studies, opinion papers, conference abstract.<br>• No digital technology described<br>• Focus on other phase than implementation, adoption, maintenance phase of digital technology<br>• No patient interaction with the technology<br>• Different target group (managerial, patient, primary care, nurse).<br>• Article describes a framework related to technology implementation<br>• No barriers or facilitators described

Oudbier SJ, *et al. BMJ Open* 2024; 14:e075833. doi: 10.1136/bmjopen-2023-075833

Supplementary Table 2. PICO with original PubMed search and results (and translated to Embase and Web of Science)

PICO*	Participants	Intervention	Context	Outcome
Research question	Medical specialists, nurse practitioners, physician assistants involved with digital technology	Digital technology based on patient interaction	Implementation, adoption, maintenance of technology in healthcare	Barriers and facilitators
PubMed	"Physicians"[Mesh] OR "Physician Assistants"[Mesh] OR "Nurse Practitioners"[Mesh] "Attitude of health personnel"[Mesh] OR "Allergist*"[tiab] OR "Anesthesiologist*"[tiab] OR "Anaesthesiologist*"[tiab] OR "Cardiologist*"[tiab] OR "Dermatologist*"[tiab] OR "Doctor*"[tiab] OR "Endocrinologist*"[tiab] OR "Gastroenterologist*"[tiab] OR "Geriatrician*"[tiab] OR "Geriatrist*"[tiab] OR "Gerontologist*"[tiab] OR "Gynaecologist*" OR "Gynecologist*"[tiab] OR "Hematologist*"[tiab] OR "Hepatologist*"[tiab] OR "Hospitalist*"[tiab] OR "Immunologist*"[tiab] OR "Infectious disease specialist*"[tiab] OR "Internist*"[tiab] OR "Intensivist*"[tiab] OR "Medical geneticist*"[tiab] OR "Medical microbiologist*"[tiab] OR "Medical specialist*"[tiab] OR "Nephrologist*"[tiab] OR "Neurologist*"[tiab] OR "Obstetrician*"[tiab] OR "Oncologist*"[tiab] OR "Ophthalmologist*"[tiab] OR "Orthopedic specialist*"[tiab] OR "Otolaryngologist*"[tiab] OR "Pathologist*"[tiab] OR "Paediatrician*"[tiab] OR "Pediatrician*"[tiab] OR "Physician*"[tiab] OR "Neonatologist*"[tiab] OR "Neurosurgeon*"[tiab] OR "Pathologist*"[tiab] OR "Physiatrist*"[tiab] OR "Psychiatrist*"[tiab] OR "Physician*"[tiab] OR "Pulmonologist*"[tiab] OR "Radiologist*"[tiab] OR "Radiotherapist*"[tiab] OR "Rheumatologist*"[tiab] OR "Surgeon*"[tiab] OR "Neurosurgeon*"[tiab] OR "Urologist*"[tiab] OR "nurse practitioner*"[tiab]	"Telemedicine"[Mesh] OR "User-Computer Interface"[Mesh] OR "Multimedia"[Mesh] OR "Cell phones"[Mesh] OR "Public health informatics"[Mesh] OR "Medical informatics"[Mesh] OR "Computers, handheld"[Mesh] OR "Mobile Applications"[Mesh] OR "Internet"[Mesh] OR "Patient Portals"[Mesh] OR "econsult*"[tiab] OR "e-consult*"[tiab] OR "ediagnos*"[tiab] OR "e diagnos*"[tiab] OR "mobile health*"[tiab] OR "mhealth*"[tiab] OR "m health*"[tiab] OR "telehealth*"[tiab] OR "tele health"[tiab] OR "remote consult*"[tiab] OR "teleconsult*"[tiab] OR "tele consult*"[tiab] OR "telediagnos*"[tiab] OR "tele diagnos*"[tiab] OR "telemedic*"[tiab] OR "tele medic*"[tiab] OR "telemonitor*"[tiab] OR "tele monitor*"[tiab] OR "ehealth*"[tiab] OR "e-health*"[tiab] OR "telecare"[tiab] OR "tele care"[tiab] OR "digital health"[tiab] OR "app"[tiab] OR "apps"[tiab] OR "smartphone*"[tiab] OR "phone application*"[tiab] OR "telephone application*"[tiab] OR "mobile application*"[tiab] OR "mobile technolog*"[tiab] OR "health technolog*"[tiab] OR "health application*"[tiab] OR "internet*"[tiab] OR "world wide web*"[tiab] OR "webportal*"[tiab] OR "web portal*"[tiab] OR "patient portal*"[tiab] OR "ipad"[tiab] OR "ipads"[tiab] OR "telecounsel*"[tiab] OR "tele counsel*"[tiab] OR "remote counsel*"[tiab] OR "distance consult*"[tiab] OR "distance counsel*"[tiab] OR "distant consult*"[tiab] OR "patient monitoring"[tiab] OR "interactive voice response*"[tiab] OR "multimedia"[tiab] OR "Mhapps"[tiab] OR "e-coach*"[tiab] OR "wearable*"[tiab] OR "handheld"[tiab] OR "virtual patient*"[tiab] OR "computer"[tiab] OR "digital*"[tiab]	"Organisational Innovation"[Mesh] OR "organisational chang*"[tiab] OR "organisational chang*"[tiab] OR "organisational innovat*"[tiab] OR "organisational innovat*"[tiab] OR "implement*"[tiab] OR "adopt*"[tiab] OR "transform*"[tiab]	No search terms included

* The C of Comparison is replaced to Context, since we did not look for studies with controlled conditions (placebo/no digital technology). Search terms between columns were combined with AND; the search is limited to publication type [systematic reviews, literature reviews and meta-analysis].

Supplementary Table 2. PubMed Results (17 June 2022)

Search	PubMed Query – June 17, 2022	Results
#5	#1 AND #2 AND #3 AND #4	4,945
#4	(("Review Literature as Topic"[Mesh] OR "Review"[Publication Type] OR "Meta-Analysis as Topic"[Mesh] OR "review*"[tiab] OR "meta-analys*"[tiab] OR "Meta-Analysis "[Publication Type] OR systematic[sb]) NOT ("Letter"[Publication Type] OR "Editorial"[Publication Type] OR "Comment"[Publication Type]))	4,149,865
#3	"Organizational Innovation"[Mesh] OR "organizational chang*"[tiab] OR "organisational chang*"[tiab] OR "organizational innovat*"[tiab] OR "organisational innovat*"[tiab] OR "implement*"[tiab] OR "adopt*"[tiab] OR "transform*"[tiab]	1,458,582
#2	"Physicians"[Mesh] OR "Physician Assistants"[Mesh] OR "Nurse Practitioners"[Mesh] "Attitude of health personnel"[Mesh] OR "Allergist*"[tiab] OR "Anesthesiologist*"[tiab] OR "Anaesthesiologist*"[tiab] OR "Cardiologist*"[tiab] OR "Dermatologist*"[tiab] OR "Doctor*"[tiab] OR "Endocrinologist*"[tiab] OR "Gastroenterologist*"[tiab] OR "Geriatrician*"[tiab] OR "Geriatrist*"[tiab] OR "Gerontologist*"[tiab] OR "Gynaecologist*" OR "Gynecologist*"[tiab] OR "Hematologist*"[tiab] OR "Hepatologist*"[tiab] OR "Hospitalist*"[tiab] OR "Immunologist*"[tiab] OR "Infectious disease specialist*"[tiab] OR "Internist*"[tiab] OR "Intensivist*"[tiab] OR "Medical geneticist*"[tiab] OR "Medical microbiologist*"[tiab] OR "Medical specialist*"[tiab] OR "Nephrologist*"[tiab] OR "Neurologist*"[tiab] OR "Obstetrician*"[tiab] OR "Oncologist*"[tiab] OR "Ophthalmologist*"[tiab] OR "Orthopedic specialist*"[tiab] OR "Otolaryngologist*"[tiab] OR "Pathologist*"[tiab] OR "Paediatrician*"[tiab] OR "Pediatrician*"[tiab] OR "Physician*"[tiab] OR "Neonatologist*"[tiab] OR "Neurosurgeon*"[tiab] OR "Pathologist*"[tiab] OR "Physiatrist*"[tiab] OR "Psychiatrist*"[tiab] OR "Physician*"[tiab] OR "Pulmonologist*"[tiab] OR "Radiologist*"[tiab] OR "Radiotherapist*"[tiab] OR "Rheumatologist*"[tiab] OR "Surgeon*"[tiab] OR "Neurosurgeon*"[tiab] OR "Urologist*"[tiab] OR "nurse practitioner*"[tiab] OR "clinician*"[tiab]	1,297,435
#1	"Telemedicine"[Mesh] OR "User-Computer Interface"[Mesh] OR "Multimedia"[Mesh] OR "Cell phones"[Mesh] OR "Public health informatics"[Mesh] OR "Medical informatics"[Mesh] OR "Nursing informatics"[Mesh] OR "Computers, handheld"[Mesh] OR "Mobile Applications"[Mesh] OR "Internet"[Mesh] OR "Patient Portals"[Mesh] OR "econsult*"[tiab] OR "e-consult*"[tiab] OR "ediagnos*"[tiab] OR "e diagnos*"[tiab] OR "mobile health*"[tiab] OR "mhealth*"[tiab] OR "m health*"[tiab] OR "telehealth*"[tiab] OR "tele health"[tiab] OR "remote consult*"[tiab] OR "teleconsult*"[tiab] OR "tele consult*"[tiab] OR "telediagnos*"[tiab] OR "tele diagnos*"[tiab] OR "telemedic*"[tiab] OR "tele medic*"[tiab] OR "telemonitor*"[tiab] OR "tele monitor*"[tiab] OR "ehealth*"[tiab] OR "e-health*"[tiab] OR "telecare"[tiab] OR "tele care"[tiab] OR "digital health"[tiab] OR "app"[tiab] OR "apps"[tiab] OR "smartphone*"[tiab] OR "phone application*"[tiab] OR "telephone application*"[tiab] OR "mobile application*"[tiab] OR "mobile technolog*"[tiab] OR "health technolog*"[tiab] OR "health application*"[tiab] OR "internet*"[tiab] OR "world wide web*"[tiab] OR "webportal*"[tiab] OR "web portal*"[tiab] OR "patient portal*"[tiab] OR "ipad"[tiab] OR "ipads"[tiab] OR "telecounsel*"[tiab] OR "tele counsel*"[tiab] OR "remote counsel*"[tiab] OR "distance consult*"[tiab] OR "distance counsel*"[tiab] OR "distant consult*"[tiab] OR "patient monitoring"[tiab] OR "interactive voice response*"[tiab] OR "multimedia"[tiab] OR "Mhapps"[tiab] OR "e-coach*"[tiab] OR "wearable*"[tiab] OR "handheld"[tiab] OR "virtual patient*"[tiab] OR "computer"[tiab] OR "digital*"[tiab]	1,098,834

Supplementary Table 2. Embase.com Results (17 June 2022)

Search	Embase Query – June 17, 2022	Results
#5	#1 AND #2 AND #3 AND #4	2,976
#4	('systematic review'/exp OR 'meta analysis'/exp OR 'review*':ab,ti,kw OR 'meta-analys*':ab,ti,kw) NOT ('conference abstract'/it OR 'conference review'/it OR 'editorial'/it OR 'erratum'/it OR 'letter'/it OR 'note'/it OR 'short survey'/it)	2,739,615
#3	'organizational chang*':ti,ab,kw OR 'organisational chang*':ti,ab,kw OR 'organizational innovat*':ti,ab,kw OR 'organisational innovat*':ti,ab,kw OR 'implement*':ti,ab,kw OR 'adopt*':ti,ab,kw OR 'transform*':ti,ab,kw	1,776,391
#2	'physician'/exp OR 'physician assistant'/exp OR 'nurse practitioner'/exp OR 'allergist*':ti,ab,kw OR 'anesthesiologist*':ti,ab,kw OR 'anaesthesiologist*':ti,ab,kw OR 'cardiologist*':ti,ab,kw OR 'dermatologist*':ti,ab,kw OR 'doctor*':ti,ab,kw OR 'endocrinologist*':ti,ab,kw OR 'gastroenterologist*':ti,ab,kw OR 'geriatrician*':ti,ab,kw OR 'geriatrist*':ti,ab,kw OR 'gerontologist*':ti,ab,kw OR 'gynaecologist*':ti,ab,kw OR 'gynecologist*':ti,ab,kw OR 'hematologist*':ti,ab,kw OR 'hepatologist*':ti,ab,kw OR 'hospitalist*':ti,ab,kw OR 'immunologist*':ti,ab,kw OR 'infectious disease specialist*':ti,ab,kw OR 'internist*':ti,ab,kw OR 'intensivist*':ti,ab,kw OR 'medical geneticist*':ti,ab,kw OR 'medical microbiologist*':ti,ab,kw OR 'medical specialist*':ti,ab,kw OR 'nephrologist*':ti,ab,kw OR 'neurologist*':ti,ab,kw OR 'obstetrician*':ti,ab,kw OR 'oncologist*':ti,ab,kw OR 'ophthalmologist*':ti,ab,kw OR 'orthopedic specialist*':ti,ab,kw OR 'otolaryngologist*':ti,ab,kw OR 'paediatrician*':ti,ab,kw OR 'pediatrician*':ti,ab,kw OR 'neonatologist*':ti,ab,kw OR 'pathologist*':ti,ab,kw OR 'physiatrist*':ti,ab,kw OR 'psychiatrist*':ti,ab,kw OR 'physician*':ti,ab,kw OR 'pulmonologist*':ti,ab,kw OR 'radiologist*':ti,ab,kw OR 'radiotherapist*':ti,ab,kw OR 'rheumatologist*':ti,ab,kw OR 'surgeon*':ti,ab,kw OR 'neurosurgeon*':ti,ab,kw OR 'urologist*':ti,ab,kw OR 'nurse practitioner*':ti,ab,kw OR 'clinician*':ti,ab,kw	2,201,667
#1	'telehealth'/de OR 'telemedicine'/de OR 'telemonitoring'/exp OR 'internet'/exp OR 'computer interface'/exp OR 'multimedia'/exp OR 'mobile phone'/exp OR 'microcomputer'/exp OR 'mobile application'/exp OR 'medical informatics'/exp OR 'econsult*':ti,ab,kw OR 'e-consult*':ti,ab,kw OR 'ediagnos*':ti,ab,kw OR 'e diagnos*':ti,ab,kw OR 'mobile health*':ti,ab,kw OR 'mhealth*':ti,ab,kw OR 'm health*':ti,ab,kw OR 'telehealth*':ti,ab,kw OR 'tele health':ti,ab,kw OR 'remote consult*':ti,ab,kw OR 'teleconsult*':ti,ab,kw OR 'tele consult*':ti,ab,kw OR 'telediagnos*':ti,ab,kw OR 'tele diagnos*':ti,ab,kw OR 'telemedic*':ti,ab,kw OR 'tele medic*':ti,ab,kw OR 'telemonitor*':ti,ab,kw OR 'tele monitor*':ti,ab,kw OR 'ehealth*':ti,ab,kw OR 'e-health*':ti,ab,kw OR 'telecare':ti,ab,kw OR 'tele care':ti,ab,kw OR 'digital health':ti,ab,kw OR 'app':ti,ab,kw OR 'apps':ti,ab,kw OR 'smartphone*':ti,ab,kw OR 'phone application*':ti,ab,kw OR 'telephone application*':ti,ab,kw OR 'mobile application*':ti,ab,kw OR 'mobile technolog*':ti,ab,kw OR 'health technolog*':ti,ab,kw OR 'health application*':ti,ab,kw OR 'internet*':ti,ab,kw OR 'world wide web*':ti,ab,kw OR 'webportal*':ti,ab,kw OR 'web portal*':ti,ab,kw OR 'patient portal*':ti,ab,kw OR 'ipad':ti,ab,kw OR 'ipads':ti,ab,kw OR 'telecounsel*':ti,ab,kw OR 'tele counsel*':ti,ab,kw OR 'remote counsel*':ti,ab,kw OR 'distance consult*':ti,ab,kw OR 'distance counsel*':ti,ab,kw OR 'distant consult*':ti,ab,kw OR 'patient monitoring':ti,ab,kw OR 'interactive voice response*':ti,ab,kw OR 'multimedia':ti,ab,kw OR 'mhapps':ti,ab,kw OR 'e-coach*':ti,ab,kw OR 'wearable*':ti,ab,kw OR 'handheld':ti,ab,kw OR 'virtual patient*':ti,ab,kw OR 'computer':ti,ab,kw OR 'digital*':ti,ab,kw	889,047

Supplementary Table 2. Clarivate Analytics/Web of Science Core Collection Results (17 June 2022)

Search	Web of Science Query – June 17, 2022	Results
#5	#1 AND #2 AND #3 AND #4	2,693
#4	TS=("review*" OR "meta-analys*")	3,174,557
#3	TS=("organizational chang*" OR "organisational chang*" OR "organizational innovat*" OR "organisational innovat*" OR "implement*" OR "adopt*" OR "transform*")	3,965,854
#2	TS=("Allergist*" OR "Anesthesiologist*" OR "Anaesthesiologist*" OR "Cardiologist*" OR "Dermatologist*" OR "Doctor*" OR "Endocrinologist*" OR "Gastroenterologist*" OR "Geriatrician*" OR "Geriatrist*" OR "Gerontologist*" OR "Gynaecologist*" OR "Gynecologist*" OR "Hematologist*" OR "Hepatologist*" OR "Hospitalist*" OR "Immunologist*" OR "Infectious disease specialist*" OR "Internist*" OR "Intensivist*" OR "Medical geneticist*" OR "Medical microbiologist*" OR "Medical specialist*" OR "Nephrologist*" OR "Neurologist*" OR "Obstetrician*" OR "Oncologist*" OR "Ophthalmologist*" OR "Orthopedic specialist*" OR "Otolaryngologist*" OR "Pathologist*" OR "Paediatrician*" OR "Pediatrician*" OR "Physician*" OR "Neonatologist*" OR "Neurosurgeon*" OR "Pathologist*" OR "Physiatrist*" OR "Psychiatrist*" OR "Physician*" OR "Pulmonologist*" OR "Radiologist*" OR "Radiotherapist*" OR "Rheumatologist*" OR "Surgeon*" OR "Neurosurgeon*" OR "Urologist*" OR "nurse practitioner*" OR "clinician*")	1,227,838
#1	TS=("econsult*" OR "e-consult*" OR "ediagnos*" OR "e diagnos*" OR "mobile health*" OR "mhealth*" OR "m health*" OR "telehealth*" OR "tele health" OR "remote consult*" OR "teleconsult*" OR "tele consult*" OR "telediagnos*" OR "tele diagnos*" OR "telemedic*" OR "tele medic*" OR "telemonitor*" OR "tele monitor*" OR "ehealth*" OR "e-health*" OR "telecare" OR "tele care" OR "digital health" OR "app" OR "apps" OR "smartphone*" OR "phone application*" OR "telephone application*" OR "mobile application*" OR "mobile technolog*" OR "health technolog*" OR "health application*" OR "internet*" OR "world wide web*" OR "webportal*" OR "web portal*" OR "patient portal*" OR "ipad" OR "ipads" OR "telecounsel*" OR "tele counsel*" OR "remote counsel*" OR "distance consult*" OR "distance counsel*" OR "distant consult*" OR "patient monitoring" OR "interactive voice response*" OR "multimedia" OR "Mhapps" OR "e-coach*" OR "wearable*" OR "handheld" OR "virtual patient*" OR "computer" OR "digital*")	1,564,093

Oudbier SJ, *et al. BMJ Open* 2024; 14:e075833. doi: 10.1136/bmjopen-2023-075833

Supplementary Table 3. Quality assessment of included studies (n=33)

**Author (year)**	**Q1** Is the review question clearly and explicitly stated?	**Q2** Were the inclusion criteria appropriate for the review question?	**Q3** Was the search strategy appropriate?	**Q4** Were the sources and resources used to search for studies adequate?	**Q5** Were the criteria for appraising the studies appropriate?[1]	**Q6** Was the critical appraisal conducted by two or more reviewers independently?[1]	**Q7** Were methods implemented to minimise errors in data extraction?	**Q8** Were the methods used to combine studies appropriate?[1,2]	**Q9** Was the likelihood of publication bias assessed?[1]	**Q10** Were recommendations for policy and/or practice supported by the reported data?	**Q11** Were the specific drivers for new research appropriate?
Almathami, Win and Vlahu-Gjorgievska (1)	Yes	Yes	Yes	Yes	Yes	**Unclear**	Yes	Yes	No	Yes	**Unclear**
Antoun (2)	Yes	**Unclear**	**Unclear**	**Unclear**	NA[*]	NA	**Unclear**	NA	NA	Yes	Yes
Appleton, Williams (3)	Yes	Yes	Yes	Yes	Yes	Yes	Yes	Yes	NA	Yes	Yes
Batterham, Sunderland (4)	Yes	Yes	**Unclear**	Yes	**Unclear**	**Unclear**	**Unclear**	Yes	NA	Yes	Yes
Bokolo (5)	Yes	Yes	Yes	Yes	NA	NA	**Unclear**	Yes	NA	Yes	Yes
Brunton, Bower and Sanders (6)	Yes	Yes	Yes	Yes	Yes	Yes	Yes	Yes	NA	Yes	Yes
Cowan, McKean (7)	Yes	**Unclear**	Yes	No	NA	NA	**Unclear**	Yes	NA	Yes	Yes
de Grood, Raissi (8)	Yes	Yes	Yes	Yes	NA	NA	Yes	Yes	NA	Yes	Yes
De Guzman, Snoswell (9)	Yes	Yes	Yes	Yes	Yes	Yes	Yes	Yes	NA	Yes	Yes
Finkelstein, Knight (10)	Yes	Yes	Yes	Yes	NA	NA	Yes	Yes	NA	Yes	Yes
Gagnon, Ngangue (11)	Yes	Yes	Yes	Yes	NA	NA	Yes	Yes	No	Yes	**Unclear**
Greenhalgh, A'Court and Shaw (12)	Yes	**Unclear**	No	Yes	NA	NA	No	Yes	NA	Yes	Yes
Hassibian and Hassibian (13)	Yes	**Unclear**	No	Yes	NA	NA	**Unclear**	NA	NA	Yes	**Unclear**
Hopstaken, Verweij (14)	Yes	Yes	Yes	Yes	Yes	Yes	Yes	Yes	NA	Yes	Yes
Jacob, Sanchez-Vazquez and Ivory (15)	Yes	Yes	Yes	Yes	Yes	**Unclear**	Yes	Yes	NA	Yes	Yes

Oudbier SJ, *et al. BMJ Open* 2024; 14:e075833. doi: 10.1136/bmjopen-2023-075833

Lewinski, Rushton (16)	Yes	Yes	Yes	Yes	Yes	**Unclear**	Yes	Yes	NA	Yes	Yes
Li, Talaei-Khoei (17)	Yes	Yes	Yes	Yes	**Unclear**	**Unclear**	Yes	Yes	NA	Yes	Yes
Liyanage-Don, Fung (18)	Yes	Yes	Yes	Yes	NA	NA	**Unclear**	Yes	NA	Yes	Yes
Maclure, Stewart and Strath (19)	Yes	Yes	Yes	Yes	Yes	Yes	Yes	Yes	NA	Yes	Yes
Meng, McAiney (20)	Yes	Yes	Yes	Yes	NA	NA	Yes	Yes	NA	Yes	Yes
Metzger, Jatana (21)	Yes	Yes	Yes	Yes	NA	NA	Yes	Yes	NA	Yes	Yes
Mileski, Kruse (22)	Yes	Yes	Yes	Yes	NA	NA	Yes	Yes	NA	Yes	No
Muir, de Boer (23)	Yes	Yes	Yes	Yes	Yes	Yes	No	Yes	NA	Yes	Yes
O'Cathail, Sivanandan (24)	Yes	Yes	Yes	Yes	NA	NA	Yes	Yes	NA	Yes	Yes
Palacholla, Fischer (25)	Yes	Yes	Yes	Yes	NA	NA	Yes	Yes	NA	Yes	Yes
Slater, Campbell (26)	Yes	Yes	Yes	Yes	Yes	Yes	Yes	Yes	NA	Yes	Yes
Smith, Jacobsen (27)	Yes	Yes	Yes	Yes	Yes	Yes	Yes	Yes	NA	Yes	Yes
Varsi, Solberg Nes (28)	Yes	Yes	Yes	Yes	Yes	No	Yes	Yes	NA	Yes	Yes
Vedel, Akhlaghpour (29)	Yes	Yes	Yes	Yes	Yes	Yes	Yes	Yes	**Unclear**	Yes	Yes
Vyas, Hambrick (30)	Yes	Yes	Yes	No	NA	NA	Yes	NA	NA	Yes	Yes
Whitelaw, Pellegrini (31)	Yes	Yes	Yes	Yes	NA	NA	Yes	Yes	NA	Yes	Yes
Xyrichis, Iliopoulou (32)	Yes	Yes	Yes	Yes	Yes	Yes	Yes	Yes	NA	Yes	Yes
Zaman, Khan (33)	Yes	Yes	Yes	Yes	NA	NA	Yes	Yes	NA	Yes	Yes

[1] Questions not applicable for other review types. [2] Question interpreted more broadly in the assessment of other review types: is the synthesis appropriate for the review question? [*] *Not applicable*

References

1. Almathami HKY, Win KT, Vlahu-Gjorgievska E. Barriers and Facilitators That Influence Telemedicine-Based, Real-Time, Online Consultation at Patients' Homes: Systematic Literature Review. J Med Internet Res. 2020;22(2):e16407.
2. Antoun J. Electronic mail communication between physicians and patients: a review of challenges and opportunities. Fam Pract. 2016;33(2):121-6.
3. Appleton R, Williams J, San Juan NV, Needle JJ, Schlief M, Jordan H, et al. Implementation, adoption, and perceptions of telemental health during the COVID-19 pandemic: systematic review. Journal of medical Internet research. 2021;23(12):e31746.
4. Batterham PJ, Sunderland M, Calear AL, Davey CG, Christensen H, Teesson M, et al. Developing a roadmap for the translation of e-mental health services for depression. Aust N Z J Psychiatry. 2015;49(9):776-84.
5. Bokolo AJ. Exploring the adoption of telemedicine and virtual software for care of outpatients during and after COVID-19 pandemic. Ir J Med Sci. 2021;190(1):1-10.
6. Brunton L, Bower P, Sanders C. The Contradictions of Telehealth User Experience in Chronic Obstructive Pulmonary Disease (COPD): A Qualitative Meta-Synthesis. PLoS One. 2015;10(10):e0139561.
7. Cowan KE, McKean AJ, Gentry MT, Hilty DM. Barriers to Use of Telepsychiatry: Clinicians as Gatekeepers. Mayo Clinic Proceedings. 2019;94(12):2510-23.
8. de Grood C, Raissi A, Kwon Y, Santana MJ. Adoption of e-health technology by physicians: a scoping review. J Multidiscip Healthc. 2016;9:335-44.
9. De Guzman KR, Snoswell CL, Taylor ML, Senanayake B, Haydon HM, Batch JA, et al. A Systematic Review of Pediatric Telediabetes Service Models. Diabetes Technol Ther. 2020;22(8):623-38.
10. Finkelstein J, Knight A, Marinopoulos S, Gibbons MC, Berger Z, Aboumatar H, et al. Enabling patient-centered care through health information technology. Evid Rep Technol Assess (Full Rep). 2012(206):1-1531.
11. Gagnon MP, Ngangue P, Payne-Gagnon J, Desmartis M. m-Health adoption by healthcare professionals: a systematic review. Journal of the American Medical Informatics Association. 2016;23(1):212-20.
12. Greenhalgh T, A'Court C, Shaw S. Understanding heart failure; explaining telehealth - a hermeneutic systematic review. BMC Cardiovasc Disord. 2017;17(1):156.
13. Hassibian MR, Hassibian S. Telemedicine acceptance and implementation in developing countries: Benefits, categories, and barriers. Razavi International Journal of Medicine. 2016;4(3).
14. Hopstaken JS, Verweij L, van Laarhoven CJ, Blijlevens NM, Stommel MW, Hermens RP. Effect of digital care platforms on quality of care for oncological patients and barriers and facilitators for their implementation: Systematic review. Journal of Medical Internet Research. 2021;23(9):e28869.
15. Jacob C, Sanchez-Vazquez A, Ivory C. Social, Organizational, and Technological Factors Impacting Clinicians' Adoption of Mobile Health Tools: Systematic Literature Review. Jmir Mhealth and Uhealth. 2020;8(2).
16. Lewinski AA, Rushton S, Van Voorhees E, Boggan JC, Whited JD, Shoup JP, et al. Implementing remote triage in large health systems: A qualitative evidence synthesis. Res Nurs Health. 2021;44(1):138-54.
17. Li J, Talaei-Khoei A, Seale H, Ray P, Macintyre CR. Health Care Provider Adoption of eHealth: Systematic Literature Review. Interact J Med Res. 2013;2(1):e7.
18. Liyanage-Don N, Fung D, Phillips E, Kronish IM. Implementing Home Blood Pressure Monitoring into Clinical Practice. Current Hypertension Reports. 2019;21(2).
19. Maclure K, Stewart D, Strath A. A systematic review of medical and non-medical practitioners' views of the impact of ehealth on shared care. European Journal of Hospital Pharmacy. 2014;21(1):54-62.

20. Meng G, McAiney C, Perlman CM, McKillop I, Tisseverasinghe T, Chen HH. Service process factors affecting patients' and clinicians' experiences on rapid teleconsultation implementation in out-patient neurology services during COVID-19 pandemic: a scoping review. BMC health services research. 2022;22(1):1-17.
21. Metzger G, Jatana K, Apfeld J, Deans KJ, Minneci PC, Halaweish I. State of telemedicine use in pediatric surgery in the USA-where we stand and what we can gain from the COVID-19 pandemic: A scoping review. World Journal of Pediatric Surgery. 2021;4(1).
22. Mileski M, Kruse CS, Catalani J, Haderer T. Adopting Telemedicine for the Self-Management of Hypertension: Systematic Review. JMIR Med Inform. 2017;5(4):e41.
23. Muir SD, de Boer K, Nedeljkovic M, Meyer D. Barriers and facilitators of videoconferencing psychotherapy implementation in veteran mental health care environments: a systematic review. BMC Health Serv Res. 2020;20(1):999.
24. O'Cathail M, Sivanandan MA, Diver C, Patel P, Christian J. The Use of Patient-Facing Teleconsultations in the National Health Service: Scoping Review. JMIR Med Inform. 2020;8(3):e15380.
25. Palacholla RS, Fischer N, Coleman A, Agboola S, Kirley K, Felsted J, et al. Provider- and Patient-Related Barriers to and Facilitators of Digital Health Technology Adoption for Hypertension Management: Scoping Review. JMIR Cardio. 2019;3(1):e11951.
26. Slater H, Campbell JM, Stinson JN, Burley MM, Briggs AM. End User and Implementer Experiences of mHealth Technologies for Noncommunicable Chronic Disease Management in Young Adults: Systematic Review. J Med Internet Res. 2017;19(12):e406.
27. Smith SM, Jacobsen JHW, Atlas AP, Khoja A, Kovoor JG, Tivey DR, et al. Telehealth in surgery: an umbrella review. ANZ journal of surgery. 2021;91(11):2360-75.
28. Varsi C, Solberg Nes L, Kristjansdottir OB, Kelders SM, Stenberg U, Zangi HA, et al. Implementation Strategies to Enhance the Implementation of eHealth Programs for Patients With Chronic Illnesses: Realist Systematic Review. J Med Internet Res. 2019;21(9):e14255.
29. Vedel I, Akhlaghpour S, Vaghefi I, Bergman H, Lapointe L. Health information technologies in geriatrics and gerontology: a mixed systematic review. Journal of the American Medical Informatics Association. 2013;20(6):1109-19.
30. Vyas KS, Hambrick HR, Shakir A, Morrison SD, Tran DC, Pearson K, et al. A Systematic Review of the Use of Telemedicine in Plastic and Reconstructive Surgery and Dermatology. Ann Plast Surg. 2017;78(6):736-68.
31. Whitelaw S, Pellegrini DM, Mamas MA, Cowie M, Van Spall HGC. Barriers and facilitators of the uptake of digital health technology in cardiovascular care: a systematic scoping review. Eur Heart J Digit Health. 2021;2(1):62-74.
32. Xyrichis A, Iliopoulou K, Mackintosh NJ, Bench S, Terblanche M, Philippou J, Sandall J. Healthcare stakeholders' perceptions and experiences of factors affecting the implementation of critical care telemedicine (CCT): qualitative evidence synthesis. Cochrane Database Syst Rev. 2021;2(2):Cd012876.
33. Zaman SB, Khan RK, Evans RG, Thrift AG, Maddison R, Islam SMS. Exploring Barriers to and Enablers of the Adoption of Information and Communication Technology for the Care of Older Adults With Chronic Diseases: Scoping Review. JMIR aging. 2022;5(1):e25251.
