# Supplementary material for: Implementation barriers and facilitators of remote monitoring, remote consultation and digital care platforms through the eyes of healthcare professionals: a review of reviews
Source: BMJ Open. 2024 Jun 10;14(6):e075833. doi: 10.1136/bmjopen-2023-075833 (PMC11168143; doi:10.1136/bmjopen-2023-075833)
Supplement: Author's manuscript [file bmjopen-2023-075833.draft_revisions.pdf]

BMJ Open is committed to open peer review. As part of this commitment we make the peer review history of every article we publish publicly available.

When an article is published we post the peer reviewers' comments and the authors' responses online. We also post the versions of the paper that were used during peer review. These are the versions that the peer review comments apply to.

The versions of the paper that follow are the versions that were submitted during the peer review process. They are not the versions of record or the final published versions. They should not be cited or distributed as the published version of this manuscript.

BMJ Open is an open access journal and the full, final, typeset and author-corrected version of record of the manuscript is available on our site with no access controls, subscription charges or pay-per-view fees (http://bmjopen.bmj.com).

If you have any questions on BMJ Open's open peer review process please email info.bmjopen@bmj.com

BMJ Open

Implementation barriers and facilitators of remote monitoring, remote consultation and digital care platforms through the eyes of healthcare professionals: a review of reviews

Journal:	*BMJ Open*
Manuscript ID	bmjopen-2023-075833
Article Type:	Original research
Date Submitted by the Author:	19-May-2023
Complete List of Authors:	Oudbier, Susan; Amsterdam Public Health Research Institute; Amsterdam Public Health Research Institute, Department of Medical Psychology<br>Souget-Ruff, S.P.; Amsterdam Public Health Research Institute, Department of Medical Psychology<br>Chen, B.S.J.; Amsterdam Public Health Research Institute, Department of Medical Psychology<br>Ziesemer, K.A.; Vrije Universiteit<br>Meij, J.J.; Amsterdam Public Health Research Institute<br>Smets, Ellen M. A.; University of Amsterdam,
Keywords:	Health informatics < BIOTECHNOLOGY & BIOINFORMATICS, Information technology < BIOTECHNOLOGY & BIOINFORMATICS, Telemedicine < BIOTECHNOLOGY & BIOINFORMATICS

SCHOLARONE™
Manuscripts

I, the Submitting Author has the right to grant and does grant on behalf of all authors of the Work (as defined in the below author licence), an exclusive licence and/or a non-exclusive licence for contributions from authors who are: i) UK Crown employees; ii) where BMJ has agreed a CC-BY licence shall apply, and/or iii) in accordance with the terms applicable for US Federal Government officers or employees acting as part of their official duties; on a worldwide, perpetual, irrevocable, royalty-free basis to BMJ Publishing Group Ltd ("BMJ") its licensees and where the relevant Journal is co-owned by BMJ to the co-owners of the Journal, to publish the Work in this journal and any other BMJ products and to exploit all rights, as set out in our licence.

The Submitting Author accepts and understands that any supply made under these terms is made by BMJ to the Submitting Author unless you are acting as an employee on behalf of your employer or a postgraduate student of an affiliated institution which is paying any applicable article publishing charge ("APC") for Open Access articles. Where the Submitting Author wishes to make the Work available on an Open Access basis (and intends to pay the relevant APC), the terms of reuse of such Open Access shall be governed by a Creative Commons licence – details of these licences and which Creative Commons licence will apply to this Work are set out in our licence referred to above.

Other than as permitted in any relevant BMJ Author's Self Archiving Policies, I confirm this Work has not been accepted for publication elsewhere, is not being considered for publication elsewhere and does not duplicate material already published. I confirm all authors consent to publication of this Work and authorise the granting of this licence.

For peer review only

Implementation barriers and facilitators of remote monitoring, remote consultation and digital care platforms through the eyes of healthcare professionals: a review of reviews

Oudbier, S.J.[a,b], MD, Souget-Ruff, S.P.[b], Chen, B.S.J.[b], Bsc, Ziesemer, K.A[c], Msc., Meij, J.J., PhD[a,d], Smets, E.M.A., PhD[b]

[a] Amsterdam UMC location Vrije Universiteit Amsterdam, Outpatient Division, Amsterdam Public Health research institute, De Boelelaan 1117, Amsterdam, The Netherlands

[b] Amsterdam UMC location AMC Amsterdam, Department of Medical Psychology, Amsterdam Public Health research institute, Meibergdreef 9, Amsterdam, The Netherlands

[c] Medical Library, Vrije Universiteit, Amsterdam, The Netherlands

[d] Amsterdam UMC, location AMC, Department of Human Genetics, Meibergdreef 9, Amsterdam, The Netherlands

Corresponding author

Susan Oudbier

Outpatient Division (VIII)

Location AMC | Tks0-136 Meibergdreef 9, 1105 AZ Amsterdam

T: 0613455752 | E: s.oudbier@amsterdamumc.nl

For peer review only

For peer review only - http://bmjopen.bmj.com/site/about/guidelines.xhtml

Abstract

Background

Digital transformation is a necessity considering the steady incline in healthcare costs, the growing aging population, and rising number of people living with chronic diseases. The usage of eHealth in patient care could be a potential solution to these societal issues, but the implementation of digital technologies that include patient interaction remains challenging. In order to navigate such complexities, healthcare professionals (HCPs) perceptions have to be considered. The objective of this review is to identify the barriers and facilitators involved in technology implementation, from the HCP perspective.

Methods

Embase.com, PubMed, and Web of Science were searched up to June 17, 2022. Search terms included digital technology, terms related to the HCP, and implementation. Quantitative and qualitative reviews evaluating digital technologies that included patient interaction were considered eligible. Three reviewers independently selected and assessed eligible reviews and conducted a critical appraisal. Regarding the data collection, two reviewers independently synthesized and interpreted data on barriers and facilitators.

Results

Thirty-three reviews met our inclusion criteria. Barriers and facilitators were categorized by four levels 1) the organisation, 2) the HCP, 3) the patient, and 4) technical aspects. Main barriers as well as facilitators on each level were (lack of) training (n=22/33), (un)familiarity with technology (n=17/33), (loss of) communication (n=13/33), and security and confidentiality issues (n=17/33).

Conclusions

Major barriers or facilitators on each level that were frequently reported by HCPs should be addressed in the implementation strategy, for instance, by adequate training, familiarizing HCPs with

For peer review only - http://bmjopen.bmj.com/site/about/guidelines.xhtml

the technology, adapting the technology to the patient preferences, and addressing technical issues. Barriers on the HCP-and patient-level can be overcome by investigating the needs of the end-users. As we shift from traditional face-to-face care models towards new modes of care delivery, further research is needed to better understand the role of digital technology in the HCP-patient relationship.

Keywords digital transformation, health care professionals, barriers, facilitators, patient interaction, digital technology, implementation

For peer review only

For peer review only - http://bmjopen.bmj.com/site/about/guidelines.xhtml

Article Summary

- This is the first umbrella review highlighting the barriers and facilitators from the end-users perspective, i.e., the HCPs, in implementing digital technology in healthcare.
- Strengths of this review include its comprehensive search on digital technologies, narrowed down to technologies that include patient interaction
- A limitation of this study is that barriers and facilitators as reported by HCPs are being in a state of constant flux. As healthcare will continue to face challenges, it is important to also consider the social environment and context within the implementation of digital technology.

Funding statement

This research received no specific grant from any funding agency in the public, commercial or not-for-profit sectors.

Competing interest statement

Enclosed.

Data sharing statement

No extra data available as presented in this review.

Word count

4000 words

For peer review only

Introduction

The introduction of the electronic health record (EHR) was a major digital transformer; it improved the exchange of data, communication between patients and health care providers (HCPs), and healthcare accessibility, thus enhancing overall quality of care (1-3). Another potential digital transformer in healthcare includes the introduction of eHealth, which is defined as 'the use of information and communications technology in support of health and health-related fields' (4). In an attempt to respond to the challenges the healthcare sector is facing, telemedicine, smartphone apps, and wearables (i.e., small electronic devices that use sensors to measure biometric values and physical activity (5)) have been developed as possible eHealth solutions. eHealth can provide efficiency gains such as improved cost-efficiency (6), enhanced patient diagnosis (7), and improved adherence to treatment and patient outcomes (8).

The importance of eHealth is growing for several reasons. Firstly, efficiency must be improved to address increasing healthcare costs, as well as demographic and epidemiological challenges such as increased life expectancy, increased rates of chronic disease, and population growth (9). Secondly, there is huge societal demand for digital transformation in healthcare, as many processes in other sectors are now digitalized. For example, passengers in the airline industry use digital technology at every phase of their journey from booking a plane ticket, to choosing a seat and checking in for flights (10). In contrast, digitalization is lagging behind in the European healthcare system due to difficulties in interoperating between systems, data security and privacy concerns, and the uptake of technologies taking longer than anticipated (11, 12). Compared to other European countries, the Netherlands has more problems with the exchange of data between healthcare sectors and international data exchanges due to an inadequate IT infrastructure, technical limitations, and lack of a central authority for standardization (13). A final reason for the need of digital transformation is related to the significant amount of data that should be stored and analysed to accelerate and to increase the efficiency of health services via big data analytics (2, 14). In order to manage these data streams efficiently and effectively, digital transformation is a

For peer review only

necessity.

Despite all these potential positive implications for healthcare, the implementation, adoption, and scaling of eHealth remains a challenge (15). Many eHealth solutions require some degree of patient interaction, such as the ability to communicate with the HCP within the EHR (i.e., to have a video-consultation or to participate in disease monitoring). Digital transformation therefore does not emerge from the enabling of technology alone; all stakeholders are required to successfully implement such technology within the healthcare system (9). This implies that in order to guarantee adoption, it is crucial to be attentive to the usability by including the end-users of the technology (e.g., the patients and HCPs), in the implementation process (16). Even though HCPs aim to deliver high-quality care, their resistance to change is a frequently mentioned barrier to the implementation of new technologies (17). Thus, understanding the barriers and facilitators as perceived by HCPs is important to enable smooth introduction and efficient use of digital technologies (17). Many studies and reviews have been executed on the subject of eHealth implementation in healthcare; however, research on factors that influence implementation of technology often focuses on a single technology (18, 19), or a specific disease (20, 21). Drawing from these reviews, the perspective of one of the key stakeholders within this implementation, that is he HCP, is often not the main focus of the study. To date, no study has systematically inventoried barriers and facilitators of implementation of digital healthcare technology concentrating on the perspective of the HCP. Furthermore, no review investigated which barriers and facilitators are generic within technology implementation, that is which barriers and facilitators apply regardless of the technology involved, and which vary per digital technology. Thus, the aim of this review is to systematically review the barriers and facilitators of implementations of digital technologies that include patient interaction, specifically focusing on the HCP's perspective.

For peer review only

Methods

This review followed the guidelines of the Preferred Reporting Items for Systematic Reviews and Meta-Analyses (PRISMA) statement (22).

Eligibility criteria

For this review, we considered all eligible literature reviews (regardless of its design) and meta-analyses studying the implementation of digital technologies applying patient interaction and barriers and/or facilitators related to HCPs. Inclusion criteria were 1) the publication type being: literature review and/or meta-analysis, 2) describing barriers, facilitators, successes, inhibitions, failures for the implementation, which includes the adoption and maintenance of the digital technology 3) the technology described should encompass patient interaction, and 4) the barriers and/or facilitators have been described from the perspective of the HCP (i.e. medical specialists, nurse practitioners, and physician assistants). A barrier is defined as any factor that hinders implementation of the technology. A facilitator is defined as something that enables the implementation of the technology. Implementation refers to the process of putting to use or integrating the digital technology into the clinical workflow (23), including the actual adoption and maintenance of the technology. The adoption of a technology is defined as the commitment or uptake of the technology by the HCPs (24), whereas the maintenance of a technology is defined as the ability to consistently achieve the health benefits of the digital technology since it was first implemented (23).

We operationalized the perspective of the HCPs by performing data extraction in three rounds. Firstly, we decided whether the HCP's perspective was the main focus of the included review by considering the title and research question. In that case, all barriers and facilitators were included in this review. Secondly, if the HCP was not a main focus of the research, we examined whether the perspective of the HCP was mentioned as a separate stakeholder in the review. If so, we included the barriers and facilitators as reported to represent their perspective. Thirdly, if healthcare

For peer review only - http://bmjopen.bmj.com/site/about/guidelines.xhtml

professionals were part of a broader group of stakeholders, including e.g. patients or managers, we discussed whether the perspective of the HCP was sufficiently exposed. In case of consensus, we included the barriers and facilitators mentioned by that review. Since there is a clear distinction between care provided outside the hospital (i.e., by the general practitioner, and care provided in the hospital, studies investigating the implementation/adoption of digital technologies in primary care have not been included). Exclusion criteria consisted of 1) article written in another language than Dutch, German, or English, 2) publication type such as research protocol, opinion paper, original empirical study, conference abstract, 3) no digital technology described, 4) focus on other phase than adoption/implementation/maintenance phase of the technology, 5) no patient interaction with the technology, 6) other target group (managerial/primary care/nurse/patient), 7) article describes a framework related to technology implementation, and 8) no barriers/facilitators described.

Information sources and search

The PICO search strategy was executed to systematically review the literature (25). In collaboration with a medical information specialist (KAZ), reviews were identified from several bibliographic databases including PubMed, Embase.com and Clarivate Analytics/Web of Science Core Collection. The search was conducted on June 29, 2021 and updated on June 17, 2022. Search terms including synonyms, closely related words and keywords were used as index terms or free-text words: “digital*”, “transform*” and “physicians”. We used a methodological search filter to specify the results to reviews, meta-analyses and other summarizing evidence. Duplicate articles were excluded using the R-package “ASYSD” an automated deduplication tool (26) followed by manual deduplication in Endnote (X20.0.3) by the medical information specialist (KAZ). The full search strategy used for each database is detailed in the supplementary material.

Study selection

For peer review only - http://bmjopen.bmj.com/site/about/guidelines.xhtml

Using the online Rayyan tool for systematic reviews, three authors independently reviewed the articles on title and abstract using the aforementioned criteria (27). The first author (SJO) reviewed all articles, and the second and the third author (BSJC, SPR) both screened 50% of the articles. Disagreements in the assessment by the reviewers were discussed until consensus was reached. A third reviewer (EMAS/JJM) was consulted when consensus could not be reached. The updated search (on June 17, 2022) was carried out by two reviewers (SJO, SPR), both authors screened 50% of the articles on the updated search. Only articles about which there was doubt were discussed until consensus was reached. In total, four articles could initially not be retrieved, one of which was eventually obtained via a librarian.

Data collection and synthesis

After duplicate removal, data from the selected reviews were independently extracted by two authors (SJO, BSJC, SPR). Disagreements regarding the extracted barriers and facilitators were addressed in a consensus meeting. The following information was extracted: 1) first author, 2) publication date, 3) type of review, 4) digital technologies reviewed, and 5) facilitators and barriers related to implementation. A meta-analysis was not performed since pooling of data was impossible due to heterogeneity of the studies investigating digital technologies and outcomes. After extraction of the barriers and facilitators per review, three authors (SJO, BSJC, SPR) had a consensus meeting to classify the barriers and facilitators. Since there was overlap between the type of digital technologies included in this review and our categorization of the type of technologies. Barriers and facilitators could be allocated to multiple categories.

We distinguished three types of technologies, for which we used definitions derived from the literature. Firstly, remote consultation (RC) is referred to as a remote consultation between a HCP and a patient by video or phone (28). Remote monitoring (RM) is defined as "an ambulatory, non-invasive digital technology used to capture patient data in real time and transmit health information for assessment by a health professional or for self-management." (29, p.4). Lastly, we

For peer review only - http://bmjopen.bmj.com/site/about/guidelines.xhtml

considered a digital care platform (DCP) as a facilitator of digital exchange between a patient and HCP, enabling interaction, coordination, or transaction (30). To ensure the best classification possible, we assigned the digital technologies described in the reviews in duplicate (SJO, SPR) to the most fitting category, and in case of overlap, into multiple categories. Finally, one reviewer (SJO) independently categorized the extracted barriers and facilitators. A second reviewer (SPR) repeated this process of categorization. Disagreements on barriers and facilitators were discussed until consensus was reached.

Quality assessment

The quality of the studies was independently assessed by three reviewers (SJO, BSJC, SPR). BSJC and SPR assessed 50% of the studies, using the Joanna Briggs Institute (JBI) approach for the quality for Systematic Reviews and Research Syntheses (31). A low-quality article is defined when less than five questions within the assessment have been answered with 'Yes'. There were no exclusions made on the basis of a minimum quality threshold. Disagreements in the assessment were resolved until consensus was reached. Some questions (Q5, Q6, and Q9) from this tool did not apply to reviews other than systematic reviews (e.g. scoping reviews); in these cases we reported these questions as not applicable. We interpreted question number 8 (Q8) broadly, as to whether or not synthesis of data was in line with the research question.

For peer review only - http://bmjopen.bmj.com/site/about/guidelines.xhtml

Results

The literature search generated a total of 10614 references: 4945 in PubMed, 2976 in Embase.com and 2693 in Web of Science Core Collection. After removing duplicates of references that were selected from more than one database, 6719 references remained. The search and screening process is illustrated in the PRISMA flow diagram in figure 1.

Review characteristics

All reviews were published between 2012 and 2021, with more than half of the reviews (n=19) published in the last five years. Most reviews were either systematic (n=17) or scoping reviews (n=8). The majority of digital technologies examined in all reviews encompassed telehealth or remote monitoring of patients (Table 1). Most of the diseases examined are cardiovascular disease (n=9), mental health problems (n=7), diabetes mellitus (DM) (n=5), and chronic obstructive pulmonary disease (n=4). Three of the included reviews reported only barriers without facilitators. Table 2 shows the overall quality assessment of the included reviews. Two articles (Antoun (2016) and Hassibian and Hassibian (2016)) scored low on the quality assessment. The main reason for reduction in quality scores was that a number of questions (Q5, Q6, Q9) did not apply because they concerned another type of review than a systematic review.

Barriers and facilitators reported by the included reviews

Generic barriers and facilitators

Barriers and facilitators from the perspective of HCPs, as identified in the included studies, have been organised into the following levels: barriers and facilitators related to the organisation, the individual HCP, the patient, and the technology. Table 3 lists the generic barriers and facilitators for all digital technology types. Overall, the included reviews report less facilitators than barriers. Major overarching themes that some reviews reported as HCP related barriers and others as facilitators

For peer review only - http://bmjopen.bmj.com/site/about/guidelines.xhtml

include (lack of) training (n= 22/33), (un)familiarity with technology (n=17/33), (loss of) communication (n=13/33), and security and confidentiality issues (n=17/33).

Generic barriers on the organisational level that were reported to be experienced by HCPs include an increase in workload (n=16/33), medico legal concerns (n=8/33), and lack of desire to change clinical paradigms (n=5/33). On the HCP level, barriers that have been reported encompass the technology possibly undermining aspects of professional identity (n=11/33), impeding the patient-clinician relationship (n=9/33), and undermining holistic surveillance (n=8/33). On the patient level, major barriers as reported by HCPs were questioning the ability of patients to use the technology (n=9/33), perceiving discomfort with engaging patients to use and to maintain to use the technology (n=4/33), and low health or literacy of patients (n=4/33). Lastly, generic barriers reported on the technological level include technical issues such as access to Wi-Fi or unreliability of the system (n=12/33), lack of harmonization of systems (n=10/33), and negative perception of the technologies aesthetics or design (n=3/33).

Generic facilitators on the organisational level as reported by HCPs included increased efficiency (n=13/33), scheduling capability and flexibility (n=7/33), use of champions (n=7/33), and new roles and responsibilities when using the digital technology (n=5/33). On the HCP level, the barriers and facilitators reported could act both ways, for instance, (lack of) motivation and engagement (n=11/33), level of satisfaction of the HCP (n=6/33), and that the technology could help influencing the decision making process (n=5/33). On the patient level, patient empowerment (n=3/33) was reported by HCPs as a facilitator for implementation. Lastly, technological barriers as reported by HCPs were also reported as facilitators, and include (lack of) usefulness and HCPs perceiving no added value of the technology (n=12/33), and (lack of) usability (n=11/33).

Barriers and facilitators divided per type of technology

For peer review only - http://bmjopen.bmj.com/site/about/guidelines.xhtml

Table 4 lists the barriers and facilitators divided by type of technology: remote consultation (n=27/33), remote monitoring (n=23/33), and digital care platforms (n=16/33). Each type of technology is discussed below.

Remote consultation

HCP related barriers regarding RC include lack of feedback or evaluation of the technology, pressure from other departments or hospitals, HCPs perceiving a sense of isolation when using the technology, and concerns of patients satisfaction and dependency. Facilitators regarding RC include good communication in network.

Remote monitoring

With regard to RM, barriers reported by HCPs involve loss of productivity during the implementation of the technology, lack of feedback or evaluation of the digital technology, a culture that is resistant to change, difficulties establishing a trusting relationship with a patient because of distance, and dependency of patients on monitoring. Facilitators specific to RM include proof of utility of the RM through research.

Digital care platforms

Barriers reported by HCP for DCP include concerns about loss of productivity during implementation, and patient satisfaction. Facilitators reported specific to DCP include a perceived competitive advantage due to the implementation of this technology by creating new opportunities, and whether the platform has proven its utility, preferably through research.

For peer review only - http://bmjopen.bmj.com/site/about/guidelines.xhtml

Table 1. Review characteristics (n=33)

Author (year)	Type of technology[c]	Description from review article	Disease/Discipline
Almathami et al. (2020)	RC	Home online health consultation systems	All health conditions (mainly cardiovascular, COPD, psychotherapy, DM)
Antoun (2016)[a]	RC	Electronic mail communication	Not specified
Appleton et al. (2021)	RC, DCP	Telemental health	Mental health problems
Batterham et al. (2015)	RC, DCP	E-mental health services	Depression
Bokolo (2021)[a]	RC, RM, DCP	Telemedicine and virtual software	For early diagnosis and follow-up
Brunton et al. (2015)	RC, RM	Telehealth	COPD
Cowan et al. (2019)[a]	RC	Tele psychiatry (2-way video therapy)	Psychiatry
de Grood et al. (2016)[b]	RM, DCP	Ehealth technology (EHR, EMR, telemedicine, health information technology, mHealth, e-Prescribing)	Not specified
De Guzman et al. (2020)	RC, RM	Telediabetes service	Paediatrics
Finkelstein et al. (2012)[a]	RC, RM, DCP	Health IT applications (telemedicine, telemonitoring, clinical decision aids, IT-guided disease management)	DM, heart disease, cancer, and other
Gagnon et al. (2016)	RC, RM, DCP	mHealth applications (smartphone, personal digital assistant, remote monitoring system, etc.)	Not specified
Greenhalgh et al. (2017)[a]	RC, RM	Telehealth (remote monitoring of symptoms, behaviour or events, biological data captured such as ECG, heart rate, blood pressure, and captured data via implantable devices)	Heart failure management
Hassibian and Hassibian (2016)[a]	RC, RM	Telemedicine (advice and consultation)	Chronic diseases (such as DM, hypertension, heart diseases)
Hopstaken et al. (2021)	DCP	Digital care platforms (general information, patient-specific information, communication possibilities with their HCP via chat, e-Consult or e-mail)*	Oncology
Jacob et al. (2020)	RM	Mobile health	Mainly acute care, COPD or congestive heart failure, cardiovascular disease, DM
Lewinski et al. (2021)	RC, RM	Telehealth to perform remote triage and monitoring	Adults and their families and caregivers
Li et al. (2013)	RC, RM, DCP	EHealth applications (EHR/EMR, Telemedicine/Telehealth, Intensive Care Information System, e-discharge, Anaesthesia Information Management System, electronic logistics information system)	Not specified
Liyanage-Don et al. (2019)[b]	RM	Home monitoring	Hypertension management
Maclure et al. (2014)	RC, RM, DCP	Ehealth on shared care (telemedicine, generalised Ehealth implementation, EHR)	Not specified
Meng et al. (2022)[b]	RC	Teleconsultation	Neurology
Metzger et al. (2021)[b]	RC	Telemedicine	Paediatric surgery
Mileski et al. (2017)	RC, RM, DCP	Telemedicine	Hypertension
Muir et al. (2020)	RC	Online videoconferencing (psycho)therapy	Veteran mental healthcare
O'Cathail et al. (2020)[b]	RC	Patient-facing teleconsultations	Accident/emergency, psychiatry/psychology, neurology
Palacholla et al. (2019)[b]	RC, RM, DCP	Digital health technology	Hypertension
Slater et al. (2017)	RM	Mobile health technologies	Non-communicable chronic diseases in young adults
Smith et al. (2021)	RC, RM, DCP	Telehealth	Surgery
Varsi et al. (2019)	RC, RM, DCP	Ehealth programmes (mHealth, telehealth, and patient portals)	Chronic conditions (arthritis, chronic pain, COPD, obesity, DM, and mental disorder)
Vedel et al. (2013)	RC, RM, DCP	Health information-/Telecare technologies (telecare, EHR, decision support systems, health information on the web)	Geriatrics, gerontology
Vyas et al. (2017)[a]	RC, RM, DCP	Telemedicine	Plastic and reconstructive surgery, dermatology
Whitelaw et al. (2021)[b]	RC, RM	Remote monitoring, tele-visits/virtual visits	Cardiovascular care
Xyrichis et al. (2021)	RM	Telemedicine	Intensive care
Zaman et al. (2022)[b]	RC, RM, DCP	Information and communication technology	Older adults with chronic diseases

COPD: Chronic Obstructive Pulmonary Disease, DCP: Digital Care Platform, DM: Diabetes Mellitus, ECG: Electrocardiogram, EHR: Electronic Health Record, EMR: Electronic Medical Record, RC: Remote Consultation, RM: Remote Monitoring, RT: Remote Triage [a] Other reviews than systematic, [b] Scoping reviews, [c] Three types of technologies were distinguished: digital care platforms (DCP), remote consultation (RC), and remote monitoring (RM).

For peer review only - http://bmjopen.bmj.com/site/about/guidelines.xhtml

Table 2. Quality assessment of included studies (n=33)

**Author (year)**	**Q1** Is the review question clearly and explicitly stated?	**Q2** Were the inclusion criteria appropriate for the review question?	**Q3** Was the search strategy appropriate?	**Q4** Were the sources and resources used to search for studies adequate?	**Q5** Were the criteria for appraising the studies appropriate?[1]	**Q6** Was the critical appraisal conducted by two or more reviewers independently?[1]	**Q7** Were the method to minimize errors in data extraction?	**Q8** Were the method used to combine studies appropriate?[1,2]	**Q9** Was the likelihood of publication bias assessed?[1]	**Q10** Were recommendations for policy and/or practice supported by the reported data?	**Q11** Were the specific drivers for new research appropriate?
Almathami et al. (2020)	Yes	Yes	Yes	Yes	Yes	**Unclear**	Yes	Yes	No	Yes	**Unclear**
Antoun (2016)	Yes	**Unclear**	**Unclear**	**Unclear**	*NA**	*NA*	**Unclear**	*NA*	*NA*	Yes	Yes
Appleton et al. (2021)	Yes	Yes	Yes	Yes	Yes	Yes	Yes	Yes	*NA*	Yes	Yes
Batterham et al. (2015)	Yes	Yes	**Unclear**	Yes	**Unclear**	**Unclear**	**Unclear**	Yes	*NA*	Yes	Yes
Bokolo (2021)	Yes	Yes	Yes	Yes	*NA*	*NA*	**Unclear**	Yes	*NA*	Yes	Yes
Brunton et al. (2015)	Yes	Yes	Yes	Yes	Yes	Yes	Yes	Yes	*NA*	Yes	Yes
Cowan et al. (2019)	Yes	**Unclear**	Yes	No	*NA*	*NA*	**Unclear**	Yes	*NA*	Yes	Yes
de Grood et al. (2016)	Yes	Yes	Yes	Yes	*NA*	*NA*	Yes	Yes	*NA*	Yes	Yes
De Guzman et al. (2020)	Yes	Yes	Yes	Yes	Yes	Yes	Yes	Yes	*NA*	Yes	Yes
Finkelstein et al. (2012)	Yes	Yes	Yes	Yes	*NA*	*NA*	Yes	Yes	*NA*	Yes	Yes
Gagnon et al. (2016)	Yes	Yes	Yes	Yes	*NA*	*NA*	Yes	Yes	No	Yes	**Unclear**
Greenhalgh et al. (2017)	Yes	**Unclear**	No	Yes	*NA*	*NA*	No	Yes	*NA*	Yes	Yes
Hassibian and Hassibian (2016)	Yes	**Unclear**	No	Yes	*NA*	*NA*	**Unclear**	*NA*	*NA*	Yes	**Unclear**
Hopstaken et al. (2021)	Yes	Yes	Yes	Yes	Yes	Yes	Yes	Yes	*NA*	Yes	Yes
Jacob et al. (2020)	Yes	Yes	Yes	Yes	Yes	**Unclear**	Yes	Yes	*NA*	Yes	Yes
Lewinski et al. (2021)	Yes	Yes	Yes	Yes	Yes	**Unclear**	Yes	Yes	*NA*	Yes	Yes
Li et al. (2013)	Yes	Yes	Yes	Yes	**Unclear**	**Unclear**	Yes	Yes	*NA*	Yes	Yes
Liyanage-Don et al. (2019)	Yes	Yes	Yes	Yes	*NA*	*NA*	**Unclear**	Yes	*NA*	Yes	Yes
Maclure et al. (2014)	Yes	Yes	Yes	Yes	Yes	Yes	Yes	Yes	*NA*	Yes	Yes
Meng et al. (2022)	Yes	Yes	Yes	Yes	*NA*	*NA*	Yes	Yes	*NA*	Yes	Yes
Metzger et al. (2021)	Yes	Yes	Yes	Yes	*NA*	*NA*	Yes	Yes	*NA*	Yes	Yes
Mileski et al. (2017)	Yes	Yes	Yes	Yes	*NA*	*NA*	Yes	Yes	*NA*	Yes	No
Muir et al. (2020)	Yes	Yes	Yes	Yes	Yes	Yes	No	Yes	*NA*	Yes	Yes
O'Cathail et al. (2020)	Yes	Yes	Yes	Yes	*NA*	*NA*	Yes	Yes	*NA*	Yes	Yes
Palacholla et al. (2019)	Yes	Yes	Yes	Yes	*NA*	*NA*	Yes	Yes	*NA*	Yes	Yes
Slater et al. (2017)	Yes	Yes	Yes	Yes	Yes	Yes	Yes	Yes	*NA*	Yes	Yes
Smith et al. (2021)	Yes	Yes	Yes	Yes	Yes	Yes	Yes	Yes	*NA*	Yes	Yes

For peer review only - http://bmjopen.bmj.com/site/about/guidelines.xhtml

Varsi et al. (2019)	Yes	Yes	Yes	Yes	Yes	No	Yes	Yes	*NA*	Yes	Yes
Vedel et al. (2013)	Yes	Yes	Yes	Yes	Yes	Yes	Yes	Yes	**Unclear**	Yes	Yes
Vyas et al. (2017)	Yes	Yes	Yes	No	*NA*	***NA***	Yes	*NA*	*NA*	Yes	Yes
Whitelaw et al. (2021)	Yes	Yes	Yes	Yes	*NA*	*NA*	Yes	Yes	*NA*	Yes	Yes
Xyrichis et al. (2021)	Yes	Yes	Yes	Yes	Yes	Yes	Yes	Yes	*NA*	Yes	Yes
Zaman et al. (2022)	Yes	Yes	Yes	Yes	*NA*	*NA*	Yes	Yes	*NA*	Yes	Yes

[1] Questions not applicable for other review types. [2]Question interpreted more broadly in the assessment of other review types: is the synthesis appropriate for the review question? [*]*Not applicable*

For peer review only

For peer review only - http://bmjopen.bmj.com/site/about/guidelines.xhtml

Table 3. Generic barriers and facilitators in digital technology implementation, from the perspective of the HCP (n=33)

Level of barrier or facilitator	Barrier or facilitator	% of Reviews	References
**Organizational**			
(Lack of) training	-/+		(34-37, 39-44, 46-51, 53, 54, 56, 59, 62, 63)
(Lack of) time and resources	-/+		(35, 40, 41, 43-45, 47-50, 53, 54, 57-59, 63, 64)
Increase in workload	-		(32, 35, 38, 40, 42-44, 46, 47, 49, 50, 53, 56, 57, 62, 63)
(Lack of) leadership and support	-/+		(35, 39-44, 46-48, 50, 51, 56, 62, 63)
(Lack of) reimbursement, financial incentives	-/+		(32, 36, 39, 40, 42, 43, 46, 48, 49, 53, 56, 57, 62)
(Lack of) integration in clinical workflow	-/+		(37, 39, 41-44, 46, 48, 49, 55, 56, 59, 63)
Increased efficiency	+		(34, 35, 39, 42, 43, 45, 46, 50, 52, 53, 55, 58, 62)
Impact on (health) outcomes	-/+		(33, 39, 43, 46, 47, 49, 50, 52, 53, 55, 56, 63)
Medico legal concerns	-		(32, 39, 40, 43, 46, 48, 50, 53)
Scheduling capability and flexibility	+		(34, 35, 39, 43, 46, 47, 55)
(Absence of) champions	+		(39-41, 44, 48, 54, 56)
Working relationships	-/+		(43, 44, 46, 47, 50, 53, 63)
Convenience (cost-efficient, travel-saving, time-saving)	-/+		(34, 44, 46, 51, 53, 58)
New roles and responsibilities	+		(46-48, 59, 63)
(Lack of) desire to change clinical paradigms	-		(36, 39, 40, 44, 64)
(Lack of) standardizations	-		(36, 42, 46, 64)
Socio-political tensions	-		(43, 47, 50)
Lack of purpose and implementation plan	-		(43, 63)
**HCP**			
(Un)familiarity with technology	-/+		(33, 36-38, 42-44, 46-49, 53-55, 60, 63, 64)
(Loss of) communication	-/+		(32, 34, 35, 43, 46, 47, 51-53, 57, 61-63)
Undermines aspects of professional identity	-		(35, 38, 40, 42-44, 46-48, 50, 53)
(Lack of) motivation and engagement	-/+		(33, 34, 43, 44, 46-48, 53, 54, 63, 64)
Impeding patient-clinician relationship	-		(36, 38, 45, 48, 51, 55, 58, 61, 62)
Undermining holistic surveillance	-		(32, 34, 35, 38, 51, 55, 58, 61)
Fear or concerns of technology	-		(32, 39, 40, 44-46)
Level of satisfaction	-/+		(35, 39, 42, 43, 52, 60)
Influence on decision making process	-/+		(40, 46, 47, 52, 57)
Attitude towards technology	-/+		(33, 39, 43, 44, 54)
Characteristics of HCP (IT literacy, age, years of practice)	-/+		(39, 42, 45, 48)
(Reduce/concerns of) overtreatment of patients	-/+		(38, 45, 61)
Threat to face-to-face services	-		(36, 39, 44)
Work-life balance	-/+		(35, 47)
**Patient**			
Questioning ability of patients	-		(34, 38, 44-46, 49, 51, 53, 64)
Adapted to preference and attitude	-/+		(43, 46, 53, 56)
Difficult to engage/maintain use	-		(34, 35, 39, 53)
Low (health/IT) literacy	-		(46, 50, 51, 53)
Patient empowerment	+		(46, 53, 56)
Enabling access for certain groups	-/+		(35, 46, 57)

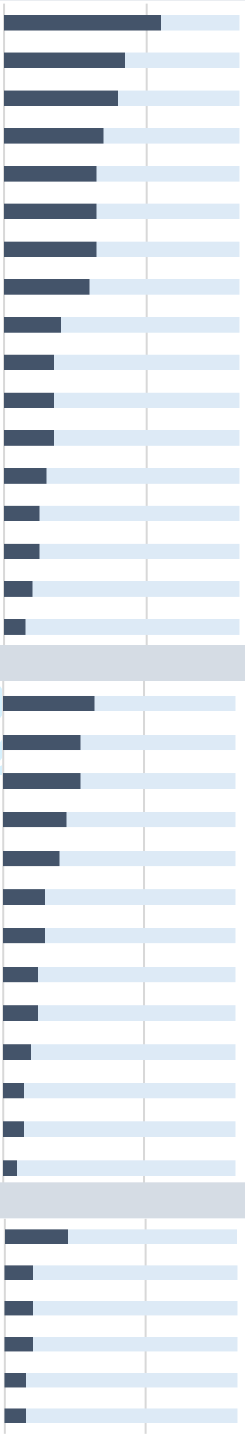

For peer review only - http://bmjopen.bmj.com/site/about/guidelines.xhtml

Technical		
Security and confidentiality (issues)	-/+	(32, 40-43, 45, 46, 48, 50, 53, 55-57, 61-64)
Technical issues (access to Wi-Fi, system unreliability)	-	(42, 43, 46, 49-52, 55-58, 62)
(Lack of) usefulness	-/+	(42, 43, 46, 47, 50, 52, 53, 55-57, 60, 62)
(Lack of) usability	-/+	(34, 40, 42, 43, 45, 46, 49, 50, 53, 55, 56)
(Lack of) harmonization of systems	-	(40, 43, 45, 46, 48-50, 53, 56, 62)
(lack of) technical support	-/+	(34, 46, 48, 51, 56, 57, 59)
Perception of aesthetics/design	-	(43, 46, 60, 62)
Availability of data	-/+	(40, 46, 49, 56)

+ indicates a facilitator, - indicates a barrier, -/+ reported as barrier as well as facilitator, HCP: Health Care Professional. The barrier or facilitator is outlined in this table when reported as barrier or facilitator in all three technologies.

For peer review only

For peer review only - http://bmjopen.bmj.com/site/about/guidelines.xhtml

Table 4. Barriers and facilitators of digital technology implementation from the perspective of the HCP, divided per type of technology.

Level of barrier or facilitator	Type of technology		
**Organizational**			
	**Remote consultation (n=27[a])**	**Remote monitoring (n=23)**	**Digital care platforms (n=16)**
Loss of productivity during implementation		(40)	(40)
Lack of feedback or evaluation	(44, 47)	(44, 47)	
Competitive advantage			(48)
Pressure from other hospitals	(62)	(62)	
Good communication in network	(52)		
Culture that is resistant to change		(46)	
**HCP**			
Sense of isolation	(39, 47)	(47)	
Difficult to establish trust relationship		(63)	
**Patient**			
Concerns of dependency of patients	(38)	(38, 46, 49)	
Patient satisfaction	(35, 39)		(35)
**Technological**			
Proof of utility (by research)		(40)	(40)

*Red means reported as barrier, green means reported as facilitator. [r] References. Orange means reported as both facilitator and barrier. RC: Remote consultation, RM: Remote Monitoring, DCP: Digital Care Platform. The barrier or facilitator is reported when reported in less than three technologies, otherwise a barrier or facilitator is reported in the generic table (Table 3).

For peer review only

For peer review only - http://bmjopen.bmj.com/site/about/guidelines.xhtml

Discussion

This systematic review of reviews aimed to investigate barriers and facilitators of using technology in patient care, as perceived by HCPs. Three types of digital technologies were distinguished: 1) Remote consultation, 2) Remote monitoring, and 3) Digital care platforms. Barriers and facilitators from the perspective of HCP could be categorized as relating to 1) the organisation, 2) the HCPs themselves, 3) the patient, and 4) the technology. Overall, our review shows that there are still major organisational requirements that must be met in order to provide a solid foundation for digital technology implementation. One way to overcome organisational barriers is by addressing them in the implementation strategy. This includes adequate training, planning ahead for potential safety and confidentiality concerns, showing the HCP the eventual benefits such as improved health outcomes by the use of the digital technology, and ensuring fully embedding within their clinical workflow. The results of this review indicate that the organisational facilitators as perceived by HCPs are comparable to the list of organisational barriers HCPs reported. Thus, these factors act as two sides of the same coin. Major facilitators at the organisational level to support a successful implementation strategy include deploying the right organisational leadership and support, making use of change champions, and ensuring good working relationships. However, as healthcare will continue to face new challenges with regard to technology implementation, barriers and facilitators are not fixed entities that need to be resolved for successful implementation. Instead, the implementation of digital technologies should be approached as a dynamic process which needs ongoing management and tailoring, taking social relations and context into account (65).

This review of reviews demonstrates that the majority of research in the past years on barriers and facilitators addressed common organisational barriers of technology implementation. Barriers on the level of the individual HCP, such as loss of communication, impeding the patient-physician relationship, undermining holistic surveillance or undermining aspects of professional identity, have been less frequently researched. For instance, a major barrier on the HCP level is loss of communication, specifically pertaining to remote consultation. As a result, HCPs experienced a

For peer review only - http://bmjopen.bmj.com/site/about/guidelines.xhtml

possibility to misunderstanding and a decreased personal connection with the patient (51, 55). In addition, HCPs perceived a difference in whether one sees the patient digitally for the first time, or whether one has already established a relationship with the patient. This distinction was particularly reported in reviews implementing digital technologies during the Coronavirus pandemic, where it became more common to have an initial digital interaction instead of face-to-face consultation (51). In addition, technology could undermine aspects of holistic surveillance, as physical examinations are not possible and it is more difficult to pick up nonverbal cues (51, 58). Despite the HCP perceiving these barriers, patients were found to be satisfied with teleconsultation and remote monitoring and did not experience this loss in communication (51, 55, 58). Therefore, more research is needed on patients as the other end-users of digital technology, and the influence of digital technology on the HCP-patient relationship.

This review also indicates training of professionals at the start of an implementation trajectory to be highly important. For transformative innovations such as the introduction of a new digital technology, training to let the user adopt and use the new technology is essential (66). However, it is unclear what exactly the training needs of HCPs are and in which frequency training should be offered. Ongoing training, rather than a one-time instruction at the start of the implementation may help in case of rapid staff changes (67). The training itself could vary from practical instructions to hands-on learning, as reported in the study by (68). At the onset, a theoretical approach to training of professionals was used, but soon they switched to practical and training sessions which emphasized building competency in how to acquire the necessary skills, such as using the app, handling swipe hand movements, and using software commands (68).

On the HCP level, major barriers included a loss of communication with patients, and the possibility of technology impeding the patient-HCP relationship. Patient connection is at the essence of being a healthcare professional, and has become more and more difficult in contemporary practice (69). A study conducted in fifty medical departments in the Netherlands showed that healthcare professionals with positive patient relationships were less likely to suffer from patient-

For peer review only - http://bmjopen.bmj.com/site/about/guidelines.xhtml

related burnout even with high job demands (69). In addition, patient-related burnout was more prevalent among HCPs who had less positive patient relationships (70). In light of these findings and with the growing amount of burnouts among HCPs (71), further research is necessary to investigate whether the use of technology in healthcare actually results in a loss of gratifying interaction with patients.

Patient related barriers, as perceived by HCPs, related to questioning the ability of patients to use digital technology, patient anxiety for using the technology, and low literacy of patients. One of the actions of the *World Health Organisation* on their global strategy is that digital technologies should strengthen health equity by promoting an inclusive digital society (72). As digital health literacy requires skills complementary to health literacy, it is important to ensure that digital technologies are available for all patients (73). Therefore, understanding and incorporating intended users' needs and context appears to be a prerequisite for effective use of digital health technology (74), apparently also from the perspective of HCP. This could be achieved by co-creation, which is described as an interaction in which participants work together to create an outcome that is appreciated by both parties (75). By including HCPs in this process, the possibility that the digital solutions will meet the needs and requirements of the end user in terms of content and usability will increase; this, in turn, may enhance technology implementation.

Strengths and limitations

The strengths of this study include its comprehensive search on digital technologies, narrowed down to technologies that include patient interaction, its duplicate approach, and its focus on the end-user. However, the current review was limited by digital technology and policies regulating these being in a state of constant flux. Therefore, barriers and facilitators reported in older studies might be less relevant over time. However, given that half of the reviews included are less than five years old, we consider the publication date to be of minor influence. Another limitation is that some of the included reviews provided little context as a result of which it was hard to categorize findings as

For peer review only - http://bmjopen.bmj.com/site/about/guidelines.xhtml

either a facilitator or a barrier in technology implementation. In such instances, the reviewers either reached a consensus-based decision or reported a finding both as a barrier and facilitator. Finally, the reviews included show differences in terms of the type of technology studied and the disease or condition in which the technology was used. The intention of this review was to provide as complete of an overview as possible of common barriers and facilitators as perceived by HCPs. All technologies in this review include a form of patient interaction, however, barriers or facilitators for a specific technology or implementation may exist.

Conclusions and implications for practice

The implementation of digital technology in healthcare is influenced by several major barriers from the perspective of the HCP that need to be overcome: security and confidentiality issues, adequate training, HCPs unfamiliarity with technology, and HCPs questioning the ability of patients to use technology. In order to successfully implement digital technology, generic barriers and facilitators as well as barriers and facilitators per type of technology have to be considered. Firstly, organisational barriers have been frequently reported and remain problematic. Such barriers can act as facilitators when addressed in the implementation strategy. Secondly, barriers on the level of the HCP and patient level are related to the patient-HCP relationship, the loss of communication, the undermining of holistic surveillance, and questioning the ability of patients. These barriers can be addressed by investigating the needs of the stakeholders involved and by making them part of the development of the digital technology by co-creation. By involving HCPs in this process, the likelihood that the digital tools meets the requirements and needs of the end-user in terms of content and usability will increase. In turn, this bolsters the chance for successful implementation. Thirdly, successful implementation of digital technology demands time and resources. Providing adequate training costs time and should be a continuous process in case of rapid staff changes. Lastly, further research is necessary on the other end-user of digital technology, the patient, in order

For peer review only - http://bmjopen.bmj.com/site/about/guidelines.xhtml

to understand the role digital technology occupies in the patient-HCP relationship, and to understand the needs of this end-user.

Ethics statements

Patient consent for publication

Not applicable.

Ethics approval

Not applicable.

Acknowledgments

The authors wish to thank Dr. L.W.P. Peute, director from the eHealth living and learning lab at the Amsterdam University Medical Center, for her support regarding the tables and figures.

Author contributions

SJO, KAZ, JJM, EMAS were involved with the design of this review. SJO, SPR, BSJC performed the screening of titles and abstracts of the included studies. With regard to data extraction and synthesis, this was performed by SJO, SPR, BSJC. SJO and SPR prepared the original draft of this review. JJM and EMAS contributed to the refinement. All authors have read and approved the final version.

For peer review only

For peer review only - http://bmjopen.bmj.com/site/about/guidelines.xhtml

References

1. Garavand A, Mohseni M, Asadi H, Etemadi M, Moradi-Joo M, Moosavi A. Factors influencing the adoption of health information technologies: a systematic review. Electronic physician. 2016;8(8):2713.

2. Kraus S, Schiavone F, Pluzhnikova A, Invernizzi AC. Digital transformation in healthcare: Analyzing the current state-of-research. Journal of Business Research. 2021;123:557-67.

3. Tsai CH, Eghdam A, Davoody N, Wright G, Flowerday S, Koch S. Effects of electronic health record implementation and barriers to adoption and use: a scoping review and qualitative analysis of the content. Life. 2020;10(12):327.

4. WHO. WHO guideline: recommendations on digital interventions for health system strengthening. Geneva: World Health Organization; 2019.

5. Smith C. What is wearable tech? Everything you need to know explained: Chris Smith; 2019 [Available from: https://www.wareable.com/wearable-tech/what-is-wearable-tech-753

6. Tortorella GL, Fogliatto FS, Espôsto KF, Mac Cawley AF, Vassolo R, Tlapa D, et al. Healthcare costs' reduction through the integration of Healthcare 4.0 technologies in developing economies. Total Quality Management & Business Excellence. 2020:1-21.

7. Shimbo D, Artinian NT, Basile JN, Krakoff LR, Margolis KL, Rakotz MK, et al. Self-measured blood pressure monitoring at home: a joint policy statement from the American Heart Association and American Medical Association. Circulation. 2020;142(4):e42-e63.

8. Hamine S, Gerth-Guyette E, Faulx D, Green BB, Ginsburg AS. Impact of mHealth chronic disease management on treatment adherence and patient outcomes: a systematic review. Journal of medical Internet research. 2015;17(2):e52.

9. Seoane F, Traver V, Hazelzet J. Value-Driven Digital Transformation in Health and Medical Care. Interactive Process Mining in Healthcare: Springer; 2021. p. 13-26.

10. Crespo-Almendros E, Del Barrio-García S. Online airline ticket purchasing: Influence of online sales promotion type and Internet experience. Journal of Air Transport Management. 2016;53:23-34.

11. Olesch A. Digialisation: a missing connectior or health systems in Europe? Eurohealth. 2022;28(3):24-8.

12. Gopal G, Suter-Crazzolara C, Toldo L, Eberhardt W. Digital transformation in healthcare–architectures of present and future information technologies. Clinical Chemistry and Laboratory Medicine (CCLM). 2019;57(3):328-35.

13. van Gemert-Pijnen LJ, editor Digitalisering in de gezondheidszorg nader beschouwd: eindrapport VWS MEVA. Digitalisering nader beschouwd, opdracht van VWS MEVA; 2022.

For peer review only - http://bmjopen.bmj.com/site/about/guidelines.xhtml

14. Manogaran G, Lopez D, Thota C, Abbas KM, Pyne S, Sundarasekar R. Big data analytics in healthcare Internet of Things. Innovative healthcare systems for the 21st century: Springer; 2017. p. 263-84.

15. Smuck M, Odonkor CA, Wilt JK, Schmidt N, Swiernik MA. The emerging clinical role of wearables: factors for successful implementation in healthcare. NPJ Digital Medicine. 2021;4(1):1-8.

16. Yen P-Y, Bakken S. Review of health information technology usability study methodologies. Journal of the American Medical Informatics Association. 2012;19(3):413-22.

17. Shahbaz M, Gao C, Zhai L, Shahzad F, Hu Y. Investigating the adoption of big data analytics in healthcare: the moderating role of resistance to change. Journal of Big Data. 2019;6(1):1-20.

18. Smoll NR, Walker J, Khandaker G. The barriers and enablers to downloading the COVIDSafe app–a topic modelling analysis. Australian and New Zealand Journal of Public Health. 2021;45(4):344-7.

19. Sangers T, Wakkee M, Kramer-Noels E, Nijsten T, Lugtenberg M. Views on mobile health apps for skin cancer screening in the general population: an in-depth qualitative exploration of perceived barriers and facilitators. British Journal of Dermatology. 2021;185(5):961-9.

20. Slevin P, Kessie T, Cullen J, Butler M, Donnelly S, Caulfield B. Exploring the barriers and facilitators for the use of digital health technologies for the management of COPD: a qualitative study of clinician perceptions. QJM: An International Journal of Medicine. 2020;113(3):163-72.

21. Foong HF, Kyaw BM, Upton Z, Tudor Car L. Facilitators and barriers of using digital technology for the management of diabetic foot ulcers: A qualitative systematic review. International wound journal. 2020;17(5):1266-81.

22. Moher D, Liberati A, Tetzlaff J, Altman DG, Group* P. Preferred reporting items for systematic reviews and meta-analyses: the PRISMA statement. Annals of internal medicine. 2009;151(4):264-9.

23. Rabin BA, Brownson RC. Terminology for dissemination and implementation research2017. 19-45 p.

24. Nilsen P. Making sense of implementation theories, models, and frameworks. Implementation Science 30: Springer; 2020. p. 53-79.

25. Liberati A, Altman DG, Tetzlaff J, Mulrow C, Gøtzsche PC, Ioannidis JP, et al. The PRISMA statement for reporting systematic reviews and meta-analyses of studies that evaluate health care interventions: explanation and elaboration. Journal of clinical epidemiology. 2009;62(10):e1-e34.

26. Hair K, Bahor Z, Macleod M, Liao J, Sena ES. The automated systematic search Deduplicator (ASySD): a rapid, open-source, interoperable tool to remove duplicate citations in biomedical systematic reviews. Biorxiv. 2021.

For peer review only

For peer review only - http://bmjopen.bmj.com/site/about/guidelines.xhtml

27. Ouzzani M, Hammady H, Fedorowicz Z, Elmagarmid A. Rayyan-a web and mobile app for systematic reviews. Systematic Reviews, 5, 210. 2016.

28. Richardson E, Aissat D, Williams GA, Fahy N. Keeping what works: remote consultations during the COVID-19 pandemic. Eurohealth. 2020;26(2):73-6.

29. Vegesna A, Tran M, Angelaccio M, Arcona S. Remote patient monitoring via non-invasive digital technologies: a systematic review. Telemedicine and e-Health. 2017;23(1):3-17.

30. Fürstenau D, Auschra C, Klein S, Gersch M. A process perspective on platform design and management: evidence from a digital platform in health care. Electronic Markets. 2019;29(4):581-96.

31. Aromataris E, Fernandez R, Godfrey CM, Holly C, Khalil H, Tungpunkom P. Summarizing systematic reviews: methodological development, conduct and reporting of an umbrella review approach. JBI Evidence Implementation. 2015;13(3):132-40.

32. Antoun J. Electronic mail communication between physicians and patients: a review of challenges and opportunities. Fam Pract. 2016;33(2):121-6.

33. Hassibian MR, Hassibian S. Telemedicine acceptance and implementation in developing countries: Benefits, categories, and barriers. Razavi International Journal of Medicine. 2016;4(3).

34. Almathami HKY, Win KT, Vlahu-Gjorgievska E. Barriers and Facilitators That Influence Telemedicine-Based, Real-Time, Online Consultation at Patients' Homes: Systematic Literature Review. J Med Internet Res. 2020;22(2):e16407.

35. Appleton R, Williams J, San Juan NV, Needle JJ, Schlief M, Jordan H, et al. Implementation, adoption, and perceptions of telemental health during the COVID-19 pandemic: systematic review. Journal of medical Internet research. 2021;23(12):e31746.

36. Batterham PJ, Sunderland M, Calear AL, Davey CG, Christensen H, Teesson M, et al. Developing a roadmap for the translation of e-mental health services for depression. Aust N Z J Psychiatry. 2015;49(9):776-84.

37. Bokolo AJ. Exploring the adoption of telemedicine and virtual software for care of outpatients during and after COVID-19 pandemic. Ir J Med Sci. 2021;190(1):1-10.

38. Brunton L, Bower P, Sanders C. The Contradictions of Telehealth User Experience in Chronic Obstructive Pulmonary Disease (COPD): A Qualitative Meta-Synthesis. PLoS One. 2015;10(10):e0139561.

39. Cowan KE, McKean AJ, Gentry MT, Hilty DM. Barriers to Use of Telepsychiatry: Clinicians as Gatekeepers. Mayo Clinic Proceedings. 2019;94(12):2510-23.

40. de Grood C, Raissi A, Kwon Y, Santana MJ. Adoption of e-health technology by physicians: a scoping review. J Multidiscip Healthc. 2016;9:335-44.

For peer review only - http://bmjopen.bmj.com/site/about/guidelines.xhtml

41. De Guzman KR, Snoswell CL, Taylor ML, Senanayake B, Haydon HM, Batch JA, et al. A Systematic Review of Pediatric Telediabetes Service Models. Diabetes Technol Ther. 2020;22(8):623-38.

42. Finkelstein J, Knight A, Marinopoulos S, Gibbons MC, Berger Z, Aboumatar H, et al. Enabling patient-centered care through health information technology. Evid Rep Technol Assess (Full Rep). 2012(206):1-1531.

43. Gagnon MP, Ngangue P, Payne-Gagnon J, Desmartis M. m-Health adoption by healthcare professionals: a systematic review. Journal of the American Medical Informatics Association. 2016;23(1):212-20.

44. Greenhalgh T, A'Court C, Shaw S. Understanding heart failure; explaining telehealth - a hermeneutic systematic review. BMC Cardiovasc Disord. 2017;17(1):156.

45. Hopstaken JS, Verweij L, van Laarhoven CJ, Blijlevens NM, Stommel MW, Hermens RP. Effect of digital care platforms on quality of care for oncological patients and barriers and facilitators for their implementation: Systematic review. Journal of Medical Internet Research. 2021;23(9):e28869.

46. Jacob C, Sanchez-Vazquez A, Ivory C. Social, Organizational, and Technological Factors Impacting Clinicians' Adoption of Mobile Health Tools: Systematic Literature Review. Jmir Mhealth and Uhealth. 2020;8(2).

47. Lewinski AA, Rushton S, Van Voorhees E, Boggan JC, Whited JD, Shoup JP, et al. Implementing remote triage in large health systems: A qualitative evidence synthesis. Res Nurs Health. 2021;44(1):138-54.

48. Li J, Talaei-Khoei A, Seale H, Ray P, Macintyre CR. Health Care Provider Adoption of eHealth: Systematic Literature Review. Interact J Med Res. 2013;2(1):e7.

49. Liyanage-Don N, Fung D, Phillips E, Kronish IM. Implementing Home Blood Pressure Monitoring into Clinical Practice. Current Hypertension Reports. 2019;21(2).

50. Maclure K, Stewart D, Strath A. A systematic review of medical and non-medical practitioners' views of the impact of ehealth on shared care. European Journal of Hospital Pharmacy. 2014;21(1):54-62.

51. Meng G, McAiney C, Perlman CM, McKillop I, Tisseverasinghe T, Chen HH. Service process factors affecting patients' and clinicians' experiences on rapid teleconsultation implementation in out-patient neurology services during COVID-19 pandemic: a scoping review. BMC health services research. 2022;22(1):1-17.

52. Metzger G, Jatana K, Apfeld J, Deans KJ, Minneci PC, Halaweish I. State of telemedicine use in pediatric surgery in the USA-where we stand and what we can gain from the COVID-19 pandemic: A scoping review. World Journal of Pediatric Surgery. 2021;4(1).

For peer review only

53. Mileski M, Kruse CS, Catalani J, Haderer T. Adopting Telemedicine for the Self-Management of Hypertension: Systematic Review. JMIR Med Inform. 2017;5(4):e41.

54. Muir SD, de Boer K, Nedeljkovic M, Meyer D. Barriers and facilitators of videoconferencing psychotherapy implementation in veteran mental health care environments: a systematic review. BMC Health Serv Res. 2020;20(1):999.

55. O'Cathail M, Sivanandan MA, Diver C, Patel P, Christian J. The Use of Patient-Facing Teleconsultations in the National Health Service: Scoping Review. JMIR Med Inform. 2020;8(3):e15380.

56. Palacholla RS, Fischer N, Coleman A, Agboola S, Kirley K, Felsted J, et al. Provider- and Patient-Related Barriers to and Facilitators of Digital Health Technology Adoption for Hypertension Management: Scoping Review. JMIR Cardio. 2019;3(1):e11951.

57. Slater H, Campbell JM, Stinson JN, Burley MM, Briggs AM. End User and Implementer Experiences of mHealth Technologies for Noncommunicable Chronic Disease Management in Young Adults: Systematic Review. J Med Internet Res. 2017;19(12):e406.

58. Smith SM, Jacobsen JHW, Atlas AP, Khoja A, Kovoor JG, Tivey DR, et al. Telehealth in surgery: an umbrella review. ANZ journal of surgery. 2021;91(11):2360-75.

59. Varsi C, Solberg Nes L, Kristjansdottir OB, Kelders SM, Stenberg U, Zangi HA, et al. Implementation Strategies to Enhance the Implementation of eHealth Programs for Patients With Chronic Illnesses: Realist Systematic Review. J Med Internet Res. 2019;21(9):e14255.

60. Vedel I, Akhlaghpour S, Vaghefi I, Bergman H, Lapointe L. Health information technologies in geriatrics and gerontology: a mixed systematic review. Journal of the American Medical Informatics Association. 2013;20(6):1109-19.

61. Vyas KS, Hambrick HR, Shakir A, Morrison SD, Tran DC, Pearson K, et al. A Systematic Review of the Use of Telemedicine in Plastic and Reconstructive Surgery and Dermatology. Ann Plast Surg. 2017;78(6):736-68.

62. Whitelaw S, Pellegrini DM, Mamas MA, Cowie M, Van Spall HGC. Barriers and facilitators of the uptake of digital health technology in cardiovascular care: a systematic scoping review. Eur Heart J Digit Health. 2021;2(1):62-74.

63. Xyrichis A, Iliopoulou K, Mackintosh NJ, Bench S, Terblanche M, Philippou J, et al. Healthcare stakeholders' perceptions and experiences of factors affecting the implementation of critical care telemedicine (CCT): qualitative evidence synthesis. Cochrane Database Syst Rev. 2021;2(2):Cd012876.

For peer review only - http://bmjopen.bmj.com/site/about/guidelines.xhtml

64. Zaman SB, Khan RK, Evans RG, Thrift AG, Maddison R, Islam SMS. Exploring Barriers to and Enablers of the Adoption of Information and Communication Technology for the Care of Older Adults With Chronic Diseases: Scoping Review. JMIR aging. 2022;5(1):e25251.

65. Checkland K, Harrison S, Marshall M. Is the metaphor of'barriers to change'useful in understanding implementation? Evidence from general medical practice. Journal of health services research & policy. 2007;12(2):95-100.

66. Gallouj F, Weinstein O. Innovation in services. Research policy. 1997;26(4-5):537-56.

67. Versluis A, van Luenen S, Meijer E, Honkoop PJ, Pinnock H, Mohr DC, et al. SERIES: eHealth in primary care. Part 4: addressing the challenges of implementation. European Journal of General Practice. 2020;26(1):140-5.

68. Dugstad J, Eide T, Nilsen ER, Eide H. Towards successful digital transformation through co-creation: a longitudinal study of a four-year implementation of digital monitoring technology in residential care for persons with dementia. BMC Health Serv Res. 2019;19(1):366.

69. Scheepers R, Silkens M, van den Berg J, Lombarts K. Associations between job demands, job resources and patient-related burnout among physicians: results from a multicentre observational study. BMJ Open. 2020;10(9):e038466.

70. Shanafelt TD. Enhancing meaning in work: a prescription for preventing physician burnout and promoting patient-centered care. Jama. 2009;302(12):1338-40.

71. Hiver C, Villa A, Bellagamba G, Lehucher-Michel MP. Burnout prevalence among European physicians: a systematic review and meta-analysis. Int Arch Occup Environ Health. 2022;95(1):259-73.

72. Organization WH. Global strategy on digital health 2020-2025. 2021.

73. Smith B, Magnani JW. New technologies, new disparities: The intersection of electronic health and digital health literacy. International Journal of Cardiology. 2019;292:280-2.

74. Thies K, Anderson D, Cramer B. Lack of Adoption of a Mobile App to Support Patient Self-Management of Diabetes and Hypertension in a Federally Qualified Health Center: Interview Analysis of Staff and Patients in a Failed Randomized Trial. JMIR Hum Factors. 2017;4(4):e24.

75. Prahalad CK, Ramaswamy V. Co-creation experiences: The next practice in value creation. Journal of interactive marketing. 2004;18(3):5-14.

For peer review only - http://bmjopen.bmj.com/site/about/guidelines.xhtml

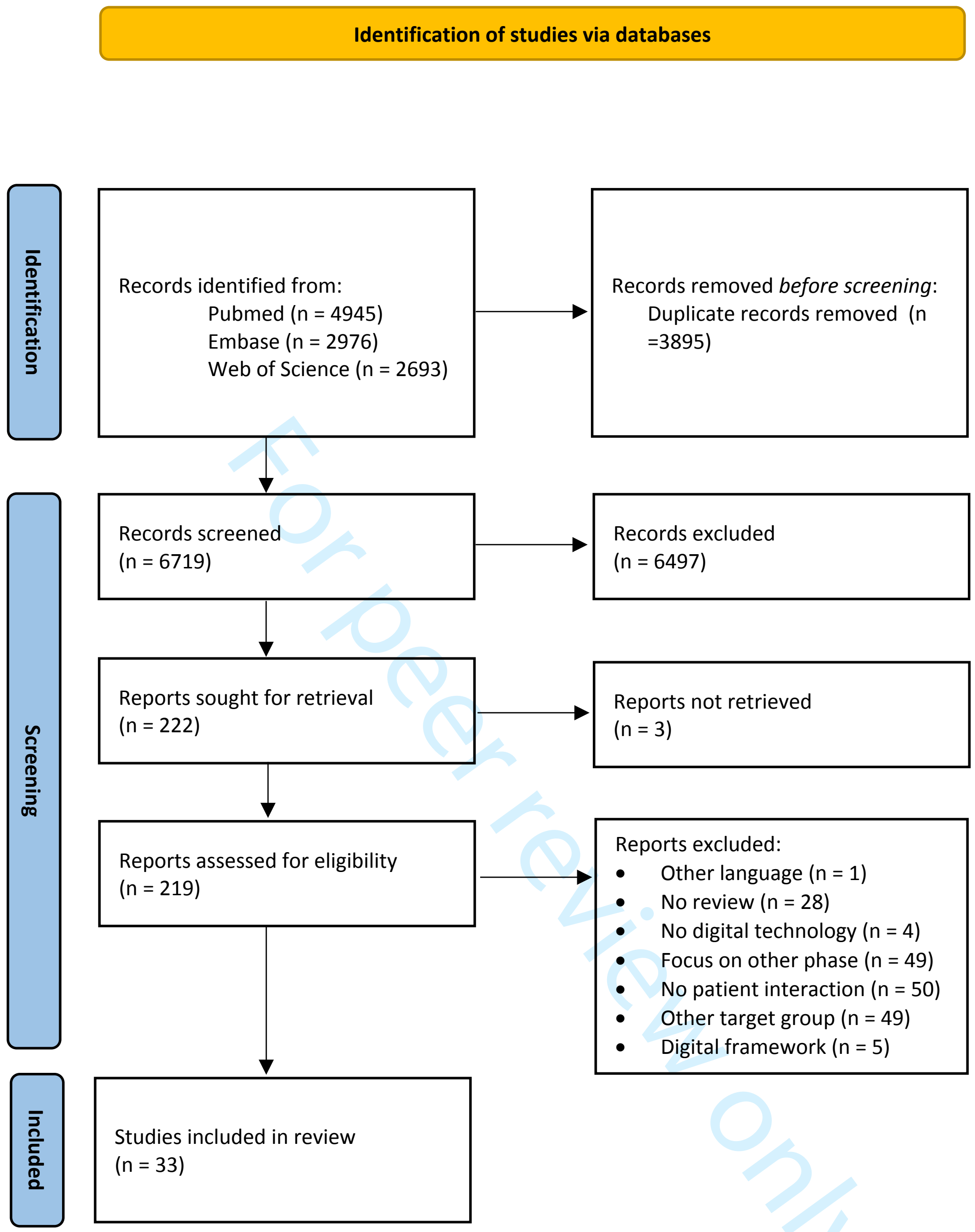

Figure 1. Flowchart of the article selection

For peer review only

Supplementary material

Table 1. In- and exclusion criteria

Inclusion criteria	Exclusion criteria
• Publication type: systematic review, meta-analysis, literature review. At least when systematic review was mentioned in title or abstract.<br>• Describing barriers, facilitators, successes, inhibitions, failures for the adoption, implementation, maintenance of technology in practice.<br>• From the healthcare professional perspective: medical specialists, nurse practitioners, and physician assistants.	• Articles written in other language than Dutch, German, or English<br>• Publication type: research protocol, empirical studies, opinion papers, conference abstract.<br>• No digital technology described<br>• Focus on other phase than implementation, adoption, maintenance phase of digital technology<br>• No patient interaction with the technology<br>• Different target group (managerial, patient, primary care, nurse).<br>• Article describes a framework related to technology implementation<br>• No barriers or facilitators described

For peer review only

Table 2. PICO with original PubMed search (translated to Embase and Web of Science)

PICO*	Participants	Intervention	Context	Outcome
Research question	Medical specialists, nurse practitioners, physician assistants involved with digital technology	Digital technology based on patient interaction	Implementation, adoption, maintenance of technology in healthcare	Barriers and facilitators
PubMed	"Physicians"[Mesh] OR "Physician Assistants"[Mesh] OR "Nurse Practitioners"[Mesh] "Attitude of health personnel"[Mesh] OR "Allergist*"[tiab] OR "Anesthesiologist*"[tiab] OR "Anaesthesiologist*"[tiab] OR "Cardiologist*"[tiab] OR "Dermatologist*"[tiab] OR "Doctor*"[tiab] OR "Endocrinologist*"[tiab] OR "Gastroenterologist*"[tiab] OR "Geriatrician*"[tiab] OR "Geriatrist*"[tiab] OR "Gerontologist*"[tiab] OR "Gynaecologist*" OR "Gynecologist*"[tiab] OR "Hematologist*"[tiab] OR "Hepatologist*"[tiab] OR "Hospitalist*"[tiab] OR "Immunologist*"[tiab] OR "Infectious disease specialist*"[tiab] OR "Internist*"[tiab] OR "Intensivist*"[tiab] OR "Medical geneticist*"[tiab] OR "Medical microbiologist*"[tiab] OR "Medical specialist*"[tiab] OR "Nephrologist*"[tiab] OR "Neurologist*"[tiab] OR "Obstetrician*"[tiab] OR "Oncologist*"[tiab] OR "Ophthalmologist*"[tiab] OR "Orthopedic specialist*"[tiab] OR "Otolaryngologist*"[tiab] OR "Pathologist*"[tiab] OR "Paediatrician*"[tiab] OR "Pediatrician*"[tiab] OR "Physician*"[tiab] OR "Neonatologist*"[tiab] OR "Neurosurgeon*"[tiab] OR "Pathologist*"[tiab] OR "Physiatrist*"[tiab] OR "Psychiatrist*"[tiab] OR "Physician*"[tiab] OR "Pulmonologist*"[tiab] OR "Radiologist*"[tiab] OR "Radiotherapist*"[tiab] OR "Rheumatologist*"[tiab] OR "Surgeon*"[tiab] OR "Neurosurgeon*"[tiab] OR "Urologist*"[tiab] OR "nurse practitioner*"[tiab]	"Telemedicine"[Mesh] OR "User-Computer Interface"[Mesh] OR "Multimedia"[Mesh] OR "Cell phones"[Mesh] OR "Public health informatics"[Mesh] OR "Medical informatics"[Mesh] OR "Computers, handheld"[Mesh] OR "Mobile Applications"[Mesh] OR "Internet"[Mesh] OR "Patient Portals"[Mesh] OR "econsult*"[tiab] OR "e-consult*"[tiab] OR "ediagnos*"[tiab] OR "e diagnos*"[tiab] OR "mobile health*"[tiab] OR "mhealth*"[tiab] OR "m health*"[tiab] OR "telehealth*"[tiab] OR "tele health"[tiab] OR "remote consult*"[tiab] OR "teleconsult*"[tiab] OR "tele consult*"[tiab] OR "telediagnos*"[tiab] OR "tele diagnos*"[tiab] OR "telemedic*"[tiab] OR "tele medic*"[tiab] OR "telemonitor*"[tiab] OR "tele monitor*"[tiab] OR "ehealth*"[tiab] OR "e-health*"[tiab] OR "telecare"[tiab] OR "tele care"[tiab] OR "digital health"[tiab] OR "app"[tiab] OR "apps"[tiab] OR "smartphone*"[tiab] OR "phone application*"[tiab] OR "telephone application*"[tiab] OR "mobile application*"[tiab] OR "mobile technolog*"[tiab] OR "health technolog*"[tiab] OR "health application*"[tiab] OR "internet*"[tiab] OR "world wide web*"[tiab] OR "webportal*"[tiab] OR "web portal*"[tiab] OR "patient portal*"[tiab] OR "ipad"[tiab] OR "ipads"[tiab] OR "telecounsel*"[tiab] OR "tele counsel*"[tiab] OR "remote counsel*"[tiab] OR "distance consult*"[tiab] OR "distance counsel*"[tiab] OR "distant consult*"[tiab] OR "patient monitoring"[tiab] OR "interactive voice response*"[tiab] OR "multimedia"[tiab] OR "Mhapps"[tiab] OR "e-coach*"[tiab] OR "wearable*"[tiab] OR "handheld"[tiab] OR "virtual patient*"[tiab] OR "computer"[tiab] OR "digital*"[tiab]	"Organisational Innovation"[Mesh] OR "organisational chang*"[tiab] OR "organisational chang*"[tiab] OR "organisational innovat*"[tiab] OR "organisational innovat*"[tiab] OR "implement*"[tiab] OR "adopt*"[tiab] OR "transform*"[tiab]	No search terms included

* The C of Comparison is replaced to Context, since we did not look for studies with controlled conditions (placebo/no digital technology). Search terms between columns were combined with AND; the search is limited to publication type [systematic reviews, literature reviews and meta-analysis].

For peer review only

PubMed Results (17 June 2022)

Search	PubMed Query – June 17, 2022	Results
#5	#1 AND #2 AND #3 AND #4	4,945
#4	(("Review Literature as Topic"[Mesh] OR "Review"[Publication Type] OR "Meta-Analysis as Topic"[Mesh] OR "review*"[tiab] OR "meta-analys*"[tiab] OR "Meta-Analysis "[Publication Type] OR systematic[sb]) NOT ("Letter"[Publication Type] OR "Editorial"[Publication Type] OR "Comment"[Publication Type]))	4,149,865
#3	"Organizational Innovation"[Mesh] OR "organizational chang*"[tiab] OR "organisational chang*"[tiab] OR "organizational innovat*"[tiab] OR "organisational innovat*"[tiab] OR "implement*"[tiab] OR "adopt*"[tiab] OR "transform*"[tiab]	1,458,582
#2	"Physicians"[Mesh] OR "Physician Assistants"[Mesh] OR "Nurse Practitioners"[Mesh] "Attitude of health personnel"[Mesh] OR "Allergist*"[tiab] OR "Anesthesiologist*"[tiab] OR "Anaesthesiologist*"[tiab] OR "Cardiologist*"[tiab] OR "Dermatologist*"[tiab] OR "Doctor*"[tiab] OR "Endocrinologist*"[tiab] OR "Gastroenterologist*"[tiab] OR "Geriatrician*"[tiab] OR "Geriatrist*"[tiab] OR "Gerontologist*"[tiab] OR "Gynaecologist*" OR "Gynecologist*"[tiab] OR "Hematologist*"[tiab] OR "Hepatologist*"[tiab] OR "Hospitalist*"[tiab] OR "Immunologist*"[tiab] OR "Infectious disease specialist*"[tiab] OR "Internist*"[tiab] OR "Intensivist*"[tiab] OR "Medical geneticist*"[tiab] OR "Medical microbiologist*"[tiab] OR "Medical specialist*"[tiab] OR "Nephrologist*"[tiab] OR "Neurologist*"[tiab] OR "Obstetrician*"[tiab] OR "Oncologist*"[tiab] OR "Ophthalmologist*"[tiab] OR "Orthopedic specialist*"[tiab] OR "Otolaryngologist*"[tiab] OR "Pathologist*"[tiab] OR "Paediatrician*"[tiab] OR "Pediatrician*"[tiab] OR "Physician*"[tiab] OR "Neonatologist*"[tiab] OR "Neurosurgeon*"[tiab] OR "Pathologist*"[tiab] OR "Physiatrist*"[tiab] OR "Psychiatrist*"[tiab] OR "Physician*"[tiab] OR "Pulmonologist*"[tiab] OR "Radiologist*"[tiab] OR "Radiotherapist*"[tiab] OR "Rheumatologist*"[tiab] OR "Surgeon*"[tiab] OR "Neurosurgeon*"[tiab] OR "Urologist*"[tiab] OR "nurse practitioner*"[tiab] OR "clinician*"[tiab]	1,297,435
#1	"Telemedicine"[Mesh] OR "User-Computer Interface"[Mesh] OR "Multimedia"[Mesh] OR "Cell phones"[Mesh] OR "Public health informatics"[Mesh] OR "Medical informatics"[Mesh] OR "Nursing informatics"[Mesh] OR "Computers, handheld"[Mesh] OR "Mobile Applications"[Mesh] OR "Internet"[Mesh] OR "Patient Portals"[Mesh] OR "econsult*"[tiab] OR "e-consult*"[tiab] OR "ediagnos*"[tiab] OR "e diagnos*"[tiab] OR "mobile health*"[tiab] OR "mhealth*"[tiab] OR "m health*"[tiab] OR "telehealth*"[tiab] OR "tele health"[tiab] OR "remote consult*"[tiab] OR "teleconsult*"[tiab] OR "tele consult*"[tiab] OR "telediagnos*"[tiab] OR "tele diagnos*"[tiab] OR "telemedic*"[tiab] OR "tele medic*"[tiab] OR "telemonitor*"[tiab] OR "tele monitor*"[tiab] OR "ehealth*"[tiab] OR "e-health*"[tiab] OR "telecare"[tiab] OR "tele care"[tiab] OR "digital health"[tiab] OR "app"[tiab] OR "apps"[tiab] OR "smartphone*"[tiab] OR "phone application*"[tiab] OR "telephone application*"[tiab] OR "mobile application*"[tiab] OR "mobile technolog*"[tiab] OR "health technolog*"[tiab] OR "health application*"[tiab] OR "internet*"[tiab] OR "world wide web*"[tiab] OR "webportal*"[tiab] OR "web portal*"[tiab] OR "patient portal*"[tiab] OR "ipad"[tiab] OR "ipads"[tiab] OR "telecounsel*"[tiab] OR "tele counsel*"[tiab] OR "remote counsel*"[tiab] OR "distance consult*"[tiab] OR "distance counsel*"[tiab] OR "distant consult*"[tiab] OR "patient monitoring"[tiab] OR "interactive voice response*"[tiab] OR "multimedia"[tiab] OR "Mhapps"[tiab] OR "e-coach*"[tiab] OR "wearable*"[tiab] OR "handheld"[tiab] OR "virtual patient*"[tiab] OR "computer"[tiab] OR "digital*"[tiab]	1,098,834

Embase.com Results (17 June 2022)

Search	Embase Query – June 17, 2022	Results
#5	#1 AND #2 AND #3 AND #4	2,976
#4	('systematic review'/exp OR 'meta analysis'/exp OR 'review*':ab,ti,kw OR 'meta-analys*':ab,ti,kw) NOT ('conference abstract'/it OR 'conference review'/it OR 'editorial'/it OR 'erratum'/it OR 'letter'/it OR 'note'/it OR 'short survey'/it)	2,739,615
#3	'organizational chang*':ti,ab,kw OR 'organisational chang*':ti,ab,kw OR 'organizational innovat*':ti,ab,kw OR 'organisational innovat*':ti,ab,kw OR 'implement*':ti,ab,kw OR 'adopt*':ti,ab,kw OR 'transform*':ti,ab,kw	1,776,391
#2	'physician'/exp OR 'physician assistant'/exp OR 'nurse practitioner'/exp OR 'allergist*':ti,ab,kw OR 'anesthesiologist*':ti,ab,kw OR 'anaesthesiologist*':ti,ab,kw OR 'cardiologist*':ti,ab,kw OR 'dermatologist*':ti,ab,kw OR 'doctor*':ti,ab,kw OR 'endocrinologist*':ti,ab,kw OR 'gastroenterologist*':ti,ab,kw OR 'geriatrician*':ti,ab,kw OR 'geriatrist*':ti,ab,kw OR 'gerontologist*':ti,ab,kw OR 'gynaecologist*':ti,ab,kw OR 'gynecologist*':ti,ab,kw OR 'hematologist*':ti,ab,kw OR 'hepatologist*':ti,ab,kw OR 'hospitalist*':ti,ab,kw OR 'immunologist*':ti,ab,kw OR 'infectious disease specialist*':ti,ab,kw OR 'internist*':ti,ab,kw OR 'intensivist*':ti,ab,kw OR 'medical geneticist*':ti,ab,kw OR 'medical microbiologist*':ti,ab,kw OR 'medical specialist*':ti,ab,kw OR 'nephrologist*':ti,ab,kw OR 'neurologist*':ti,ab,kw OR 'obstetrician*':ti,ab,kw OR 'oncologist*':ti,ab,kw OR 'ophthalmologist*':ti,ab,kw OR 'orthopedic specialist*':ti,ab,kw OR 'otolaryngologist*':ti,ab,kw OR 'paediatrician*':ti,ab,kw OR 'pediatrician*':ti,ab,kw OR 'neonatologist*':ti,ab,kw OR 'pathologist*':ti,ab,kw OR 'physiatrist*':ti,ab,kw OR 'psychiatrist*':ti,ab,kw OR 'physician*':ti,ab,kw OR 'pulmonologist*':ti,ab,kw OR 'radiologist*':ti,ab,kw OR 'radiotherapist*':ti,ab,kw OR 'rheumatologist*':ti,ab,kw OR 'surgeon*':ti,ab,kw OR 'neurosurgeon*':ti,ab,kw OR 'urologist*':ti,ab,kw OR 'nurse practitioner*':ti,ab,kw OR 'clinician*':ti,ab,kw	2,201,667
#1	'telehealth'/de OR 'telemedicine'/de OR 'telemonitoring'/exp OR 'internet'/exp OR 'computer interface'/exp OR 'multimedia'/exp OR 'mobile phone'/exp OR 'microcomputer'/exp OR 'mobile application'/exp OR 'medical informatics'/exp OR 'econsult*':ti,ab,kw OR 'e-consult*':ti,ab,kw OR 'ediagnos*':ti,ab,kw OR 'e diagnos*':ti,ab,kw OR 'mobile health*':ti,ab,kw OR 'mhealth*':ti,ab,kw OR 'm health*':ti,ab,kw OR 'telehealth*':ti,ab,kw OR 'tele health':ti,ab,kw OR 'remote consult*':ti,ab,kw OR 'teleconsult*':ti,ab,kw OR 'tele consult*':ti,ab,kw OR 'telediagnos*':ti,ab,kw OR 'tele diagnos*':ti,ab,kw OR 'telemedic*':ti,ab,kw OR 'tele medic*':ti,ab,kw OR 'telemonitor*':ti,ab,kw OR 'tele monitor*':ti,ab,kw OR 'ehealth*':ti,ab,kw OR 'e-health*':ti,ab,kw OR 'telecare':ti,ab,kw OR 'tele care':ti,ab,kw OR 'digital health':ti,ab,kw OR 'app':ti,ab,kw OR 'apps':ti,ab,kw OR 'smartphone*':ti,ab,kw OR 'phone application*':ti,ab,kw OR 'telephone application*':ti,ab,kw OR 'mobile application*':ti,ab,kw OR 'mobile technolog*':ti,ab,kw OR 'health technolog*':ti,ab,kw OR 'health application*':ti,ab,kw OR 'internet*':ti,ab,kw OR 'world wide web*':ti,ab,kw OR 'webportal*':ti,ab,kw OR 'web portal*':ti,ab,kw OR 'patient portal*':ti,ab,kw OR 'ipad':ti,ab,kw OR 'ipads':ti,ab,kw OR 'telecounsel*':ti,ab,kw OR 'tele counsel*':ti,ab,kw OR 'remote counsel*':ti,ab,kw OR 'distance consult*':ti,ab,kw OR 'distance counsel*':ti,ab,kw OR 'distant consult*':ti,ab,kw OR 'patient monitoring':ti,ab,kw OR 'interactive voice response*':ti,ab,kw OR 'multimedia':ti,ab,kw OR 'mhapps':ti,ab,kw OR 'e-coach*':ti,ab,kw OR 'wearable*':ti,ab,kw OR 'handheld':ti,ab,kw OR 'virtual patient*':ti,ab,kw OR 'computer':ti,ab,kw OR 'digital*':ti,ab,kw	889,047

For peer review only

Clarivate Analytics/Web of Science Core Collection Results (17 June 2022)

Search	Web of Science Query – June 17, 2022	Results
#5	#1 AND #2 AND #3 AND #4	2,693
#4	TS=(“review*” OR “meta-analys*”)	3,174,557
#3	TS=("organizational chang*" OR "organisational chang*" OR "organizational innovat*" OR "organisational innovat*" OR "implement*" OR "adopt*" OR “transform*”)	3,965,854
#2	TS=("Allergist*" OR "Anesthesiologist*" OR "Anaesthesiologist*" OR "Cardiologist*" OR "Dermatologist*" OR "Doctor*" OR "Endocrinologist*" OR "Gastroenterologist*" OR "Geriatrician*" OR "Geriatrist*" OR “Gerontologist*” OR "Gynaecologist*" OR "Gynecologist*" OR “Hematologist*” OR “Hepatologist*” OR "Hospitalist*" OR “Immunologist*” OR “Infectious disease specialist*” OR "Internist*" OR "Intensivist*" OR “Medical geneticist*” OR "Medical microbiologist*" OR "Medical specialist*" OR "Nephrologist*" OR "Neurologist*" OR "Obstetrician*" OR "Oncologist*" OR "Ophthalmologist*" OR “Orthopedic specialist*” OR "Otolaryngologist*" OR "Pathologist*" OR "Paediatrician*" OR "Pediatrician*" OR "Physician*" OR "Neonatologist*" OR "Neurosurgeon*" OR “Pathologist*” OR "Physiatrist*" OR "Psychiatrist*" OR "Physician*" OR "Pulmonologist*" OR "Radiologist*" OR "Radiotherapist*" OR "Rheumatologist*" OR "Surgeon*" OR "Neurosurgeon*" OR "Urologist*" OR "nurse practitioner*" OR "clinician*")	1,227,838
#1	TS=(“econsult*” OR “e-consult*” OR “ediagnos*” OR “e diagnos*” OR “mobile health*” OR “mhealth*” OR “m health*” OR “telehealth*” OR “tele health” OR “remote consult*” OR “teleconsult*” OR “tele consult*” OR “telediagnos*” OR “tele diagnos*” OR “telemedic*” OR “tele medic*” OR “telemonitor*” OR “tele monitor*” OR “ehealth*” OR “e-health*” OR “telecare” OR “tele care” OR “digital health” OR “app” OR “apps” OR “smartphone*” OR “phone application*” OR “telephone application*” OR “mobile application*” OR “mobile technolog*” OR “health technolog*” OR “health application*” OR “internet*” OR “world wide web*” OR “webportal*” OR “web portal*” OR “patient portal*” OR “ipad” OR “ipads” OR “telecounsel*” OR “tele counsel*” OR “remote counsel*” OR “distance consult*” OR “distance counsel*” OR “distant consult*” OR “patient monitoring” OR “interactive voice response*” OR “multimedia” OR “Mhapps” OR “e-coach*” OR “wearable*” OR “handheld” OR “virtual patient*” OR “computer” OR “digital*”)	1,564,093

For peer review only

Reporting checklist for systematic review (with or without a meta-analysis).

Based on the PRISMA guidelines.

Instructions to authors

Complete this checklist by entering the page numbers from your manuscript where readers will find each of the items listed below.

Your article may not currently address all the items on the checklist. Please modify your text to include the missing information. If you are certain that an item does not apply, please write "n/a" and provide a short explanation.

Upload your completed checklist as an extra file when you submit to a journal.

In your methods section, say that you used the PRISMAreporting guidelines, and cite them as:

Page MJ, McKenzie JE, Bossuyt PM, Boutron I, Hoffmann TC, Mulrow CD, Shamseer L, Tetzlaff JM, Akl EA, Brennan SE, Chou R, Glanville J, Grimshaw JM, Hróbjartsson A, Lalu MM, Li T, Loder EW, Mayo-Wilson E, McDonald S, McGuinness LA, Stewart LA, Thomas J, Tricco AC, Welch VA, Whiting P, Moher D. The PRISMA 2020 statement: An updated guideline for reporting systematic reviews

		Reporting Item	Page Number
**Title**			
Title	#1	Identify the report as a systematic review	1
**Abstract**			

For peer review only

Abstract	#2	Report an abstract addressing each item in the PRISMA 2020 for Abstracts checklist	2
**Introduction**			
Background/rationale	#3	Describe the rationale for the review in the context of existing knowledge	5
Objectives	#4	Provide an explicit statement of the objective(s) or question(s) the review addresses	5
**Methods**			
Eligibility criteria	#5	Specify the inclusion and exclusion criteria for the review and how studies were grouped for the syntheses	7
Information sources	#6	Specify all databases, registers, websites, organisations, reference lists, and other sources searched or consulted to identify studies. Specify the date when each source was last searched or consulted	8
Search strategy	#7	Present the full search strategies for all databases, registers, and websites, including any filters and limits used	8
Selection process	#8	Specify the methods used to decide whether a study met the inclusion criteria of the review, including how many reviewers screened each record and each report retrieved, whether they worked independently, and, if applicable, details of automation tools used in the process	9

For peer review only

Data collection process	#9	Specify the methods used to collect data from reports, including how many reviewers collected data from each report, whether they worked independently, any processes for obtaining or confirming data from study investigators, and, if applicable, details of automation tools used in the process	9
Data items	#10a	List and define all outcomes for which data were sought. Specify whether all results that were compatible with each outcome domain in each study were sought (for example, for all measures, time points, analyses), and, if not, the methods used to decide which results to collect	9
Study risk of bias assessment	#11	Specify the methods used to assess risk of bias in the included studies, including details of the tool(s) used, how many reviewers assessed each study and whether they worked independently, and, if applicable, details of automation tools used in the process	10
Effect measures	#12	Specify for each outcome the effect measure(s) (such as risk ratio, mean difference) used in the synthesis or presentation of results	NA
Synthesis methods	#13a	Describe the processes used to decide which studies were eligible for each synthesis (such as tabulating the study intervention characteristics and comparing against the planned groups for each synthesis (item #5))	9-10

For peer review only

Synthesis methods	#13b	Describe any methods required to prepare the data for presentation or synthesis, such as handling of missing summary statistics or data conversions	9-10
Synthesis methods	#13c	Describe any methods used to tabulate or visually display results of individual studies and syntheses	9-10
Synthesis methods	#13d	Describe any methods used to synthesise results and provide a rationale for the choice(s). If meta-analysis was performed, describe the model(s), method(s) to identify the presence and extent of statistical heterogeneity, and software package(s) used	9-10
Synthesis methods	#13e	Describe any methods used to explore possible causes of heterogeneity among study results (such as subgroup analysis, meta-regression)	NA
Synthesis methods	#13f	Describe any sensitivity analyses conducted to assess robustness of the synthesised results	NA
Reporting bias assessment	#14	Describe any methods used to assess risk of bias due to missing results in a synthesis (arising from reporting biases)	NA
Certainty assessment	#15	Describe any methods used to assess certainty (or confidence) in the body of evidence for an outcome	NA
Data items	#10b	List and define all other variables for which data were sought (such as participant and intervention characteristics, funding sources). Describe any	9

For peer review only

		assumptions made about any missing or unclear information	

Results

Study selection	#16a	Describe the results of the search and selection process, from the number of records identified in the search to the number of studies included in the review, ideally using a flow diagram (http://www.prisma-statement.org/PRISMAStatement/FlowDiagram)	11
Study selection	#16b	Cite studies that might appear to meet the inclusion criteria, but which were excluded, and explain why they were excluded	11
Study characteristics	#17	Cite each included study and present its characteristics	14
Risk of bias in studies	#18	Present assessments of risk of bias for each included study	NA
Results of individual studies	#19	For all outcomes, present for each study (a) summary statistics for each group (where appropriate) and (b) an effect estimate and its precision (such as confidence/credible interval), ideally using structured tables or plots	NA
Results of syntheses	#20a	For each synthesis, briefly summarise the characteristics and risk of bias among contributing studies	NA
Results of syntheses	#20b	Present results of all statistical syntheses conducted. If meta-analysis was done, present for each the summary	NA

For peer review only

		estimate and its precision (such as confidence/credible interval) and measures of statistical heterogeneity. If comparing groups, describe the direction of the effect	
Results of syntheses	#20c	Present results of all investigations of possible causes of heterogeneity among study results	NA
Results of syntheses	#20d	Present results of all sensitivity analyses conducted to assess the robustness of the synthesised results	NA
Risk of reporting biases in syntheses	#21	Present assessments of risk of bias due to missing results (arising from reporting biases) for each synthesis assessed	NA
Certainty of evidence	#22	Present assessments of certainty (or confidence) in the body of evidence for each outcome assessed	NA
**Discussion**			
Results in context	#23a	Provide a general interpretation of the results in the context of other evidence	20
Limitations of included studies	#23b	Discuss any limitations of the evidence included in the review	22
Limitations of the review methods	#23c	Discuss any limitations of the review processes used	22
Implications	#23d	Discuss implications of the results for practice, policy, and future research	23
**Other information**			

For peer review only

Registration and protocol	#24a	Provide registration information for the review, including register name and registration number, or state that the review was not registered	NA
Registration and protocol	#24b	Indicate where the review protocol can be accessed, or state that a protocol was not prepared	n/a
Registration and protocol	#24c	Describe and explain any amendments to information provided at registration or in the protocol	NA
Support	#25	Describe sources of financial or non-financial support for the review, and the role of the funders or sponsors in the review	24
Competing interests	#26	Declare any competing interests of review authors	24
Availability of data, code, and other materials	#27	Report which of the following are publicly available and where they can be found: template data collection forms; data extracted from included studies; data used for all analyses; analytic code; any other materials used in the review	24

The PRISMA checklist is distributed under the terms of the Creative Commons Attribution License CC-BY. This checklist was completed on 19. May 2023 using https://www.goodreports.org/, a tool made by the EQUATOR Network in collaboration with Penelope.ai

For peer review only

BMJ Open

Implementation barriers and facilitators of remote monitoring, remote consultation and digital care platforms through the eyes of healthcare professionals: a review of reviews

Journal:	*BMJ Open*
Manuscript ID	bmjopen-2023-075833.R1
Article Type:	Original research
Date Submitted by the Author:	14-Mar-2024
Complete List of Authors:	Oudbier, Susan; Amsterdam UMC Location VUmc, Outpatient Division, Amsterdam Public Health research institute, Location Vrije Universiteit Amsterdam, De Boelelaan 1117; Amsterdam UMC Location AMC, Department of Medical Psychology, Amsterdam Public Health research institute, Meibergdreef 9<br>Souget-Ruff, S.P.; Amsterdam UMC Location AMC, Department of Medical Psychology, Amsterdam Public Health research institute, Meibergdreef 9<br>Chen, B.S.J.; Amsterdam UMC Location AMC, Department of Medical Psychology, Amsterdam Public Health research institute, Meibergdreef 9<br>Ziesemer, K.A.; Vrije Universiteit Amsterdam, Medical Library<br>Meij, J.J.; Amsterdam UMC Location VUmc, Outpatient Division, Amsterdam Public Health research institute, Location Vrije Universiteit Amsterdam, De Boelelaan 1117; National University of Singapore Yong Loo Lin School of Medicine<br>Smets, Ellen M. A.; Amsterdam UMC Location AMC, Department of Medical Psychology, Amsterdam Public Health research institute, Meibergdreef 9
**Primary Subject Heading**:	Health informatics
Secondary Subject Heading:	Communication, Health informatics, Medical management, Public health
Keywords:	Health informatics < BIOTECHNOLOGY & BIOINFORMATICS, Information technology < BIOTECHNOLOGY & BIOINFORMATICS, Telemedicine < BIOTECHNOLOGY & BIOINFORMATICS

SCHOLARONE™
Manuscripts

I, the Submitting Author has the right to grant and does grant on behalf of all authors of the Work (as defined in the below author licence), an exclusive licence and/or a non-exclusive licence for contributions from authors who are: i) UK Crown employees; ii) where BMJ has agreed a CC-BY licence shall apply, and/or iii) in accordance with the terms applicable for US Federal Government officers or employees acting as part of their official duties; on a worldwide, perpetual, irrevocable, royalty-free basis to BMJ Publishing Group Ltd ("BMJ") its licensees and where the relevant Journal is co-owned by BMJ to the co-owners of the Journal, to publish the Work in this journal and any other BMJ products and to exploit all rights, as set out in our licence.

The Submitting Author accepts and understands that any supply made under these terms is made by BMJ to the Submitting Author unless you are acting as an employee on behalf of your employer or a postgraduate student of an affiliated institution which is paying any applicable article publishing charge ("APC") for Open Access articles. Where the Submitting Author wishes to make the Work available on an Open Access basis (and intends to pay the relevant APC), the terms of reuse of such Open Access shall be governed by a Creative Commons licence – details of these licences and which Creative Commons licence will apply to this Work are set out in our licence referred to above.

Other than as permitted in any relevant BMJ Author's Self Archiving Policies, I confirm this Work has not been accepted for publication elsewhere, is not being considered for publication elsewhere and does not duplicate material already published. I confirm all authors consent to publication of this Work and authorise the granting of this licence.

For peer review only

Implementation barriers and facilitators of remote monitoring, remote consultation and digital care platforms through the eyes of healthcare professionals: a review of reviews

Oudbier, S.J.[a,b], MD, Souget-Ruff, S.P.[b], Chen, B.S.J.[b], Bsc, Ziesemer, K.A[c], Msc., Meij, J.J., PhD[a,d], Smets, E.M.A., PhD[b]

[a] Amsterdam UMC location Vrije Universiteit Amsterdam, Outpatient Division, Amsterdam Public Health research institute, De Boelelaan 1117, Amsterdam, The Netherlands

[b] Amsterdam UMC location AMC Amsterdam, Department of Medical Psychology, Amsterdam Public Health research institute, Meibergdreef 9, Amsterdam, The Netherlands

[c] Medical Library, Vrije Universiteit, Amsterdam, The Netherlands

[d] Yong Loo Lin School of Medicine, National University of Singapore, Singapore.

Corresponding author

Susan Oudbier

Outpatient Division (VIII)

Location AMC | Tks0-136 Meibergdreef 9, 1105 AZ Amsterdam

T: 0613455752 | E: s.oudbier@amsterdamumc.nl

For peer review only - http://bmjopen.bmj.com/site/about/guidelines.xhtml

Abstract

Objectives

Digital transformation in healthcare is a necessity considering the steady increase in healthcare costs, the growing ageing population, and rising number of people living with chronic diseases. The implementation of digital health technologies in patient care is a potential solution to these issues, however, some challenges remain. In order to navigate such complexities, the perceptions of healthcare professionals (HCPs) must be considered. The objective of this umbrella review is to identify key barriers and facilitators involved in digital health technology implementation, from the perspective of HCPs.

Design

Systematic umbrella review following the Preferred Reporting Items for Systematic Reviews and Meta-Analyses (PRISMA) statement.

Data sources

Embase.com, PubMed, and Web of Science Core Collection were searched for existing reviews dated up to June 17, 2022. Search terms included digital health technology, combined with terms related to implementation, and variations in terms encompassing HCP, such as physician, doctor, and the medical discipline.

Eligibility criteria

Quantitative and qualitative reviews evaluating digital technologies that included patient interaction were considered eligible. Three reviewers independently synthesised and assessed eligible reviews and conducted a critical appraisal.

Data extraction and synthesis

For peer review only - http://bmjopen.bmj.com/site/about/guidelines.xhtml

Regarding the data collection, two reviewers independently synthesized and interpreted data on barriers and facilitators.

Results

Thirty-three reviews met the inclusion criteria. Barriers and facilitators were categorised into four levels; 1) the organisation, 2) the HCP, 3) the patient, and 4) technical aspects. The main barriers and facilitators identified were (lack of) training (n=22/33), (un)familiarity with technology (n=17/33), (loss of) communication (n=13/33), and security and confidentiality issues (n=17/33). Barriers of key importance included increased workload (n=16/33), the technology undermining aspects of professional identity (n=11/33), HCP uncertainty about patients' aptitude with the technology (n=9/33), and technical issues (n=12/33).

Conclusions

The implementation strategy should address the key barriers highlighted by HCPs, for instance, by providing adequate training to familiarise HCPs with the technology, adapting the technology to the patient preferences, and addressing technical issues. Barriers on both HCP-and patient-levels can be overcome by investigating the needs of the end-users. As we shift from traditional face-to-face care models towards new modes of care delivery, further research is needed to better understand the role of digital technology in the HCP-patient relationship.

Keywords digital transformation, health care professionals, barriers, facilitators, patient interaction, digital technology, implementation

For peer review only - http://bmjopen.bmj.com/site/about/guidelines.xhtml

Strengths and limitations of this study

- We conducted a review of reviews (umbrella review) on digital health implementation
- This systematic review encompasses digital health technologies which include patient interaction
- The guidelines of the Preferred Reporting Items for Systematic Reviews and Meta-Analyses (PRISMA) statement were followed
- The quality assessment of the reviews was assessed in duplicate, following the JBI approach for systematic reviews

Word count: 4200

For peer review only

For peer review only - http://bmjopen.bmj.com/site/about/guidelines.xhtml

Introduction

The introduction of the Electronic Health Record (EHR) was a major digital transformer. EHR improved overall quality of care by increasing healthcare accessibility, communication, and the exchange of data between patients and health care providers (HCPs)(1-3). The introduction of digital health, defined by the *World Health Organisation* as 'the use of information and communications technology in support of health and health-related fields', promises to be another innovation in healthcare (4). Telemedicine, smartphone apps, and wearables (i.e., small electronic devices that use sensors to measure biometric values and physical activity) (5) are examples of digital health technologies that have been developed to address shortcomings in the healthcare sector. Digital health is expected to lead to improved cost-efficiency (6), enhanced patient diagnosis (7), and improved adherence to treatment and patient outcomes (8).

The importance of digital health is growing for several reasons. Firstly, efficiency must be improved to address increasing healthcare costs, as well as demographic and epidemiological challenges such as increased life expectancy, increased rates of chronic diseases, and population growth (9). Secondly, there is huge societal demand for digital transformation in healthcare, as many processes in other sectors are now digitalized. For example, passengers in the airline industry use digital technology at every phase of their journey from booking a plane ticket, to choosing a seat and checking in for flights (10). In contrast, digitalization is lagging behind in the European healthcare system. This is due to difficulties in interoperating between systems, data security and privacy concerns, and the uptake of technologies taking longer than anticipated (11, 12). Inadequate IT infrastructure, technical limitations, and lack of a central authority for standardisation mean that the Netherlands face more challenges with the exchange of data between healthcare sectors, and international data exchanges, than other European countries (13). A final reason for the need of digital transformation in healthcare is related to the significant data storage requirements needed to

For peer review only - http://bmjopen.bmj.com/site/about/guidelines.xhtml

accelerate and increase the efficiency of health services via big data analytics (2, 14). In order to manage these data streams efficiently and effectively, digital transformation is a necessity.

Despite all these positive implications for healthcare, the implementation, adoption, and scaling of digital health remains a challenge (15). Many digital health technologies require some degree of patient interaction, such as the ability to communicate with the HCP within the EHR (i.e., to have a video-consultation or to participate in disease monitoring). Digital transformation therefore does not emerge from the enabling of technology alone; all stakeholders are required to successfully implement such technology within the healthcare system (9). This implies that in order to guarantee adoption, it is crucial to be attentive to the usability by including the end-users of the technology (e.g., the patients and HCPs), in the implementation process (16). Even though HCPs aim to deliver high-quality care, their resistance to change is a frequently mentioned barrier to the implementation of new technologies (17).

Thus, understanding the barriers and facilitators as perceived by HCPs is important to enable smooth introduction and efficient use of digital health technologies (17). Many studies and reviews have been executed on the subject of implementation in healthcare; however, research on factors that influence implementation of technology often focuses on a single technology (18, 19), or a specific disease (20, 21). Drawing from these reviews, the perspective of one of the key stakeholders within this implementation, that is the HCP, is often not the main focus of the study. To date, no study has systematically inventoried barriers and facilitators of implementation of digital health technology concentrating on the perspective of the HCP. Furthermore, no review investigated which barriers and facilitators are generic within technology implementation. That is, which barriers and facilitators apply regardless of the technology involved, and which vary per digital technology. Thus, the aim of this review is to systematically review the barriers and facilitators of implementations of digital health technologies that include patient interaction, specifically focusing on the HCP's perspective.

For peer review only

Methods

This review followed the guidelines of the Preferred Reporting Items for Systematic Reviews and Meta-Analyses (PRISMA) statement (22).

Eligibility criteria

For this review, we considered all eligible literature reviews (regardless of design) and meta-analyses studying the implementation of digital health technologies applying patient interaction and barriers and/or facilitators related to HCPs. Inclusion criteria were 1) the publication type being: literature review and/or meta-analysis, 2) describing barriers, facilitators, successes, inhibitions, and/or failures for the implementation, adoption and maintenance of the digital health technology 3) the technology described should encompass patient interaction, and 4) the barriers and/or facilitators have been described from the perspective of the HCP (i.e. medical specialists, nurse practitioners, and physician assistants) (Supplementary Table 1). A barrier is defined as any factor that hinders implementation of the technology. A facilitator is defined as something that enables the implementation of the technology. Implementation refers to the process of putting to use or integrating the digital health technology into the clinical workflow (23), including the actual adoption and maintenance of the technology. The adoption of a technology is defined as the commitment or uptake of the technology by the HCPs (24), whereas the maintenance of a technology is defined as the ability to consistently achieve the benefits of the digital technology after implementation (23). We operationalised the perspective of the HCPs by performing data extraction in three rounds. Firstly, we decided whether the HCP's perspective was the main focus of the included review by considering the title and research question. In that case, all barriers and facilitators were included in this review. Secondly, if the HCP was not a main focus of the research, we examined whether the perspective of the HCP was mentioned as a separate stakeholder in the review. If so, we included the barriers and facilitators as reported to represent their perspective. Thirdly, if healthcare professionals were part of a broader group of stakeholders, including e.g. patients or managers, we

For peer review only - http://bmjopen.bmj.com/site/about/guidelines.xhtml

discussed whether the perspective of the HCP was sufficiently exposed. In case of consensus, we included the barriers and facilitators mentioned by that review. Since there is a clear distinction between care provided outside the hospital (i.e., by the general practitioner, and care provided in the hospital, studies investigating the implementation/adoption of digital technologies in primary care have not been included). Exclusion criteria consisted of 1) article written in language other than Dutch, German, or English, 2) publication type such as research protocol, opinion paper, original empirical study, conference abstract, 3) no digital health technology described, 4) focus on phase other than adoption/implementation/maintenance phase of the technology, 5) no patient interaction with the technology, 6) different target user (managerial/primary care/nurse/patient), 7) article describes a framework related to technology implementation, and 8) no barriers/facilitators described.

Information sources and search

The PICO search strategy was executed to systematically review the literature (25). In collaboration with a medical information specialist (KAZ), reviews were identified from several bibliographic databases including PubMed, Embase.com and Clarivate Analytics/Web of Science Core Collection. The search was conducted on June 29, 2021 and updated on June 17, 2022. Search terms including synonyms, closely related words and keywords were used as index terms or free-text words: “digital*”, “transform*” and “physicians”. We used a methodological search filter to specify the results to reviews, meta-analyses and other summarising evidence. Duplicate articles were excluded using the R-package “ASYSD” an automated deduplication tool (26) followed by manual deduplication in Endnote (X20.0.3) by the medical information specialist (KAZ). The full search strategy used for each database is detailed in the supplementary material (Supplementary Table 2).

Study selection

Using the online Rayyan tool for systematic reviews, three authors independently reviewed the articles on title and abstract using the aforementioned criteria (27). The first author (SJO) reviewed

For peer review only - http://bmjopen.bmj.com/site/about/guidelines.xhtml

all articles, and the second and the third author (BSJC, SPR) both screened 50% of the articles. Disagreements in the assessment by the reviewers were discussed until consensus was reached. A third reviewer (EMAS/JJM) was consulted when consensus could not be reached. The updated search (on June 17, 2022) was carried out by two reviewers (SJO, SPR), both authors screened 50% of the articles on the updated search. Articles about which there was doubt were discussed until consensus was reached. In total, four articles could initially not be retrieved, one of which was eventually obtained via a librarian.

Data collection and synthesis

After duplicate removal, data from the selected reviews were independently extracted by two authors (SJO, BSJC, SPR). Disagreements regarding the extracted barriers and facilitators were addressed in a consensus meeting. The following information was extracted: 1) first author, 2) publication date, 3) type of review, 4) digital health technologies reviewed, and 5) facilitators and barriers related to implementation. A meta-analysis was not performed since pooling of data was impossible due to heterogeneity of the studies investigating digital technologies and outcomes. After extraction of the barriers and facilitators per review, three authors (SJO, BSJC, SPR) had a consensus meeting to classify the barriers and facilitators. Since there was overlap between the type of digital health technologies included in this review and our categorisation of the type of technologies, barriers and facilitators could be allocated to multiple categories.

We distinguished three types of digital health technologies, for which we used definitions derived from the literature. Firstly, remote consultation (RC) is referred to as a remote consultation between a HCP and a patient by video or phone (28). Remote monitoring (RM) is defined as "an ambulatory, non-invasive digital technology used to capture patient data in real time and transmit health information for assessment by a health professional or for self-management." (29, p.4). Lastly, we considered a digital care platform (DCP) as a facilitator of digital exchange between a patient and HCP, enabling interaction, coordination, or transaction (30). To ensure the best classification

For peer review only - http://bmjopen.bmj.com/site/about/guidelines.xhtml

possible, we assigned the digital technologies described in the reviews in duplicate (SJO, SPR) to the most fitting category, and in case of overlap, into multiple categories. Finally, one reviewer (SJO) independently categorised the extracted barriers and facilitators. A second reviewer (SPR) repeated this process of categorisation. Disagreements on barriers and facilitators were discussed until consensus was reached.

Quality assessment

The quality of the studies was independently assessed by three reviewers (SJO, BSJC, SPR). BSJC and SPR assessed 50% of the studies, using the Joanna Briggs Institute (JBI) approach for the quality for Systematic Reviews and Research Syntheses (31). A low-quality article is defined when less than five questions within the assessment have been answered with 'Yes'. There were no exclusions made on the basis of a minimum quality threshold. Disagreements in the assessment were resolved until consensus was reached. Some questions (Q5, Q6, and Q9) from this tool did not apply to reviews other than systematic reviews (e.g. scoping reviews); in these cases we reported these questions as not applicable. We interpreted question number 8 (Q8) broadly, as to whether or not synthesis of data was in line with the research question.

Patient and Public Involvement

No patient involved.

For peer review only - http://bmjopen.bmj.com/site/about/guidelines.xhtml

Results

The literature search generated a total of 10614 references: 4945 in PubMed, 2976 in Embase.com and 2693 in Web of Science Core Collection. After removing duplicates of references that were selected from more than one database, 6719 references remained. The search and screening process is illustrated in the PRISMA flow diagram in Figure 1.

Review characteristics

All reviews were published between 2012 and 2021, with more than half of the reviews (n=19) published in the last five years. Most reviews were either systematic (n=17) or scoping reviews (n=8). The majority of digital technologies examined in all reviews encompassed telehealth or remote monitoring of patients (Table 1). Most of the diseases examined were cardiovascular disease (n=9), mental health (n=7), diabetes mellitus (DM) (n=5), and chronic obstructive pulmonary disease (n=4). Three of the included reviews reported only barriers without facilitators. The full search quality assessment is detailed in the supplementary material (Supplementary Table 3) Two articles (Antoun (32) and Hassibian and Hassibian (33)) scored low on the quality assessment. The main reason for reduction in quality scores was that a number of questions (Q5, Q6, Q9) did not apply because they concerned another type of review than a systematic review.

Barriers and facilitators reported by the included reviews

Generic barriers and facilitators

Barriers and facilitators from the perspective of HCPs, as identified in the included studies, have been organised into the following levels: barriers and facilitators related to the organisation, the individual HCP, the patient, and the technology. Table 2 lists the generic barriers and facilitators for all digital technology types. Overall, the included reviews report less facilitators than barriers. Major overarching themes that some reviews reported as HCP related barriers and others as facilitators

For peer review only - http://bmjopen.bmj.com/site/about/guidelines.xhtml

include (lack of) training (n= 22/33), (un)familiarity with technology (n=17/33), (loss of) communication (n=13/33), and security and confidentiality issues (n=17/33).

Generic barriers on the organisational level that were reported to be experienced by HCPs include an increased workload (n=16/33), medico legal concerns (n=8/33), and lack of desire to change clinical paradigms (n=5/33). On the HCP level, barriers that have been reported encompass the technology possibly undermining aspects of professional identity (n=11/33), impeding the patient-clinician relationship (n=9/33), and undermining holistic surveillance (n=8/33). On the patient level, major barriers as reported by HCPs were questioning the ability of patients to use the technology (n=9/33), perceiving discomfort with engaging patients to use and to maintain to use the technology (n=4/33), and low health- or general literacy of patients (n=4/33). Lastly, generic barriers reported on the technological level include technical issues such as access to Wi-Fi or unreliability of the system (n=12/33), lack of harmonisation of systems (n=10/33), and negative perception of the technologies aesthetics or design (n=3/33).

Generic facilitators on the organisational level as reported by HCPs included increased efficiency (n=13/33), scheduling capability and flexibility (n=7/33), use of champions (n=7/33), and new roles and responsibilities when using the digital technology (n=5/33). On the HCP level, the barriers and facilitators reported could act both ways, for instance, (lack of) motivation and engagement (n=11/33), level of satisfaction of the HCP (n=6/33), and that the technology could help influencing the decision making process (n=5/33). On the patient level, patient empowerment (n=3/33) was reported by HCPs as a facilitator for implementation. Lastly, technological barriers as reported by HCPs were also reported as facilitators, and include (lack of) usefulness and HCPs perceiving no added value of the technology (n=12/33), and (lack of) usability (n=11/33).

Barriers and facilitators divided per type of technology

For peer review only - http://bmjopen.bmj.com/site/about/guidelines.xhtml

Table 3 lists the barriers and facilitators divided by type of technology: remote consultation (n=27/33), remote monitoring (n=23/33), and digital care platforms (n=16/33). Each type of technology is discussed below.

Remote consultation

HCP related barriers regarding RC include lack of feedback or evaluation of the technology, pressure from other departments or hospitals, HCPs perceiving a sense of isolation when using the technology, and concerns of patients satisfaction and dependency. Facilitators regarding RC include good communication in network with other stakeholders.

Remote monitoring

With regard to RM, barriers reported by HCPs involve loss of productivity during the implementation of the digital health technology, lack of feedback or evaluation of the digital technology, a culture that is resistant to change, difficulties establishing a trusting relationship with a patient because of distance, and dependency of patients on monitoring. Facilitators specific to RM include proof of utility of the RM through research.

Digital care platforms

Barriers reported by HCP for DCP include concerns about loss of productivity during implementation, and reduced patient satisfaction. Facilitators reported that were specific to DCP include a perceived competitive advantage due to the implementation of this technology by creating new opportunities, and proven utility of the platform, preferably through research.

For peer review only - http://bmjopen.bmj.com/site/about/guidelines.xhtml

Table 1. Review characteristics (n=33)

Author (year)	Type of technology[c]	Description from review article	Disease/Discipline
Almathami, Win and Vlahu-Gjorgievska (34)	RC	Home online health consultation systems	All health conditions (mainly cardiovascular, COPD, psychotherapy, DM)
Antoun (32)[a]	RC	Electronic mail communication	Not specified
Appleton, Williams (35)	RC, DCP	Telemental health	Mental health problems
Batterham, Sunderland (36)	RC, DCP	E-mental health services	Depression
Bokolo (37)[a]	RC, RM, DCP	Telemedicine and virtual software	For early diagnosis and follow-up
Brunton, Bower and Sanders (38)	RC, RM	Telehealth	COPD
Cowan, McKean (39)[a]	RC	Tele psychiatry (2-way video therapy)	Psychiatry
de Grood, Raissi (40)[b]	RM, DCP	Ehealth technology (EHR, EMR, telemedicine, health information technology, mHealth, e-Prescribing)	Not specified
De Guzman, Snoswell (41)	RC, RM	Telediabetes service	Paediatrics
Finkelstein, Knight (42)[a]	RC, RM, DCP	Health IT applications (telemedicine, telemonitoring, clinical decision aids, IT-guided disease management)	DM, heart disease, cancer, and other
Gagnon, Ngangue (43)	RC, RM, DCP	mHealth applications (smartphone, personal digital assistant, remote monitoring system, etc.)	Not specified
Greenhalgh, A'Court and Shaw (44)[a]	RC, RM	Telehealth (remote monitoring of symptoms, behaviour or events, biological data captured such as ECG, heart rate, blood pressure, and captured data via implantable devices)	Heart failure management
Hassibian and Hassibian (33)[a]	RC, RM	Telemedicine (advice and consultation)	Chronic diseases (such as DM, hypertension, heart diseases)
Hopstaken, Verweij (45)	DCP	Digital care platforms (general information, patient-specific information, communication possibilities with their HCP via chat, e-Consult or e-mail)*	Oncology
Jacob, Sanchez-Vazquez and Ivory (46)	RM	Mobile health	Mainly acute care, COPD or congestive heart failure, cardiovascular disease, DM
Lewinski, Rushton (47)	RC, RM	Telehealth to perform remote triage and monitoring	Adults and their families and caregivers
Li, Talaei-Khoei (48)	RC, RM, DCP	EHealth applications (EHR/EMR, Telemedicine/Telehealth, Intensive Care Information System, e-discharge, Anaesthesia Information Management System, electronic logistics information system)	Not specified
Liyanage-Don, Fung (49)[b]	RM	Home monitoring	Hypertension management
Maclure, Stewart and Strath (50)	RC, RM, DCP	Ehealth on shared care (telemedicine, generalised Ehealth implementation, EHR)	Not specified
Meng, McAiney (51)[b]	RC	Teleconsultation	Neurology
Metzger, Jatana (52)[b]	RC	Telemedicine	Paediatric surgery
Mileski, Kruse (53)	RC, RM, DCP	Telemedicine	Hypertension
Muir, de Boer (54)	RC	Online videoconferencing (psycho)therapy	Veteran mental healthcare
O'Cathail, Sivanandan (55)[b]	RC	Patient-facing teleconsultations	Accident/emergency, psychiatry/psychology, neurology
Palacholla, Fischer (56)[b]	RC, RM, DCP	Digital health technology	Hypertension
Slater, Campbell (57)	RM	Mobile health technologies	Non-communicable chronic diseases in young adults
Smith, Jacobsen (58)	RC, RM, DCP	Telehealth	Surgery
Varsi, Solberg Nes (59)	RC, RM, DCP	Ehealth programmes (mHealth, telehealth, and patient portals)	Chronic conditions (arthritis, chronic pain, COPD, obesity, DM, and mental disorder)
Vedel, Akhlaghpour (60)	RC, RM, DCP	Health information-/Telecare technologies (telecare, EHR, decision support systems, health information on the web)	Geriatrics, gerontology
Vyas, Hambrick (61)[a]	RC, RM, DCP	Telemedicine	Plastic and reconstructive surgery, dermatology
Whitelaw, Pellegrini (62)[b]	RC, RM	Remote monitoring, tele-visits/virtual visits	Cardiovascular care
Xyrichis, Iliopoulou (63)	RM	Telemedicine	Intensive care
Zaman, Khan (64)[b]	RC, RM, DCP	Information and communication technology	Older adults with chronic diseases

COPD: Chronic Obstructive Pulmonary Disease, DCP: Digital Care Platform, DM: Diabetes Mellitus, ECG: Electrocardiogram, EHR: Electronic Health Record, EMR: Electronic Medical Record, RC: Remote Consultation, RM: Remote Monitoring, RT: Remote Triage [a] Other reviews than systematic, [b] Scoping reviews, [c] Three types of technologies were distinguished: digital care platforms (DCP), remote consultation (RC), and remote monitoring (RM)

For peer review only - http://bmjopen.bmj.com/site/about/guidelines.xhtml

Table 2. Generic barriers and facilitators in digital technology implementation, from the perspective of the HCP (n=33)

Level of barrier or facilitator	Barrier (-) Facilitator (+)	# of reviews	% of Reviews	References
**Organisational**				
(Lack of) training	-/+	22/33		(34-37, 39-44, 46-51, 53, 54, 56, 59, 62, 63)
(Lack of) time and resources	-/+	17/33		(35, 40, 41, 43-45, 47-50, 53, 54, 57-59, 63, 64)
Increase in workload	-	16/33		(32, 35, 38, 40, 42-44, 46, 47, 49, 50, 53, 56, 57, 62, 63)
(Lack of) leadership and support	-/+	15/33		(35, 39-44, 46-48, 50, 51, 56, 62, 63)
(Lack of) reimbursement, financial incentives	-/+	13/33		(32, 36, 39, 40, 42, 43, 46, 48, 49, 53, 56, 57, 62)
(Lack of) integration in clinical workflow	-/+	13/33		(37, 39, 41-44, 46, 48, 49, 55, 56, 59, 63)
Increased efficiency	+	13/33		(34, 35, 39, 42, 43, 45, 46, 50, 52, 53, 55, 58, 62)
Impact on (health) outcomes	-/+	12/33		(33, 39, 43, 46, 47, 49, 50, 52, 53, 55, 56, 63)
Medico legal concerns	-	8/33		(32, 39, 40, 43, 46, 48, 50, 53)
Scheduling capability and flexibility	+	7/33		(34, 35, 39, 43, 46, 47, 55)
(Absence of) champions	+	7/33		(39-41, 44, 48, 54, 56)
Working relationships	-/+	7/33		(43, 44, 46, 47, 50, 53, 63)
Convenience (cost-efficient, travel-saving, time-saving)	-/+	6/33		(34, 44, 46, 51, 53, 58)
New roles and responsibilities	+	5/33		(46-48, 59, 63)
(Lack of) desire to change clinical paradigms	-	5/33		(36, 39, 40, 44, 64)
(Lack of) standardizations	-	4/33		(36, 42, 46, 64)
Socio-political tensions	-	3/33		(43, 47, 50)
Lack of purpose and implementation plan	-	2/33		(43, 63)
**HCP**				
(Un)familiarity with technology	-/+	17/33		(33, 36-38, 42-44, 46-49, 53-55, 60, 63, 64)
(Loss of) communication	-/+	13/33		(32, 34, 35, 43, 46, 47, 51-53, 57, 61-63)
Undermines aspects of professional identity	-	11/33		(35, 38, 40, 42-44, 46-48, 50, 53)
(Lack of) motivation and engagement	-/+	11/33		(33, 34, 43, 44, 46-48, 53, 54, 63, 64)
Impeding patient-clinician relationship	-	9/33		(36, 38, 45, 48, 51, 55, 58, 61, 62)
Undermining holistic surveillance	-	8/33		(32, 34, 35, 38, 51, 55, 58, 61)
Fear or concerns of technology	-	6/33		(32, 39, 40, 44-46)
Level of satisfaction	-/+	6/33		(35, 39, 42, 43, 52, 60)
Influence on decision making process	-/+	5/33		(40, 46, 47, 52, 57)
Attitude towards technology	-/+	5/33		(33, 39, 43, 44, 54)
Characteristics of HCP (IT literacy, age, years of practice)	-/+	4/33		(39, 42, 45, 48)
(Reduce/concerns of) overtreatment of patients	-/+	3/33		(38, 45, 61)
Threat to face-to-face services	-	3/33		(36, 39, 44)
Work-life balance	-/+	2/33		(35, 47)
**Patient**				
Questioning ability of patients	-	9/33		(34, 38, 44-46, 49, 51, 53, 64)
Patient tailored approach	-/+	4/33		(43, 46, 53, 56)
Difficult to engage/maintain use	-	4/33		(34, 35, 39, 53)
Low (health/IT) literacy	-	4/33		(46, 50, 51, 53)
Patient empowerment	+	3/33		(46, 53, 56)
Enabling access for certain groups	-/+	3/33		(35, 46, 57)

For peer review

For peer review only - http://bmjopen.bmj.com/site/about/guidelines.xhtml

Technical			
Security and confidentiality (issues)	-/+	17/33	(32, 40-43, 45, 46, 48, 50, 53, 55-57, 61-64)
Technical issues (access to Wi-Fi, system unreliability)	-	12/33	(42, 43, 46, 49-52, 55-58, 62)
(Lack of) usefulness	-/+	12/33	(42, 43, 46, 47, 50, 52, 53, 55-57, 60, 62)
(Lack of) usability	-/+	11/33	(34, 40, 42, 43, 45, 46, 49, 50, 53, 55, 56)
(Lack of) harmonisation of systems	-	10/33	(40, 43, 45, 46, 48-50, 53, 56, 62)
(lack of) technical support	-/+	7/33	(34, 46, 48, 51, 56, 57, 59)
Perception of aesthetics/design	-	4/33	(43, 46, 60, 62)
Availability of data	-/+	4/33	(40, 46, 49, 56)

+ indicates a facilitator, - indicates a barrier, -/+ reported as barrier as well as facilitator, HCP: Health Care Professional. The barrier or facilitator is outlined in this table when reported as barrier or facilitator in all three technologies.

For peer review only

Table 3. Barriers and facilitators of digital technology implementation from the perspective of the HCP, divided per type of technology.

Level of barrier or facilitator	Type of technology		
**Organisational**			
	**Remote consultation (n=27)**	**Remote monitoring (n=23)**	**Digital care platforms (n=16)**
Loss of productivity during implementation		(40)[a]	(40)
Lack of feedback or evaluation	(44, 47)	(44, 47)	
Competitive advantage			(48)
Pressure from other hospitals	(62)	(62)	
Good communication in network	(52)		
Culture that is resistant to change		(46)	
**HCP**			
Sense of isolation	(39, 47)	(47)	
Difficult to establish trust relationship		(63)	
**Patient**			
Concerns of dependency of patients	(38)	(38, 46, 49)	
Patient satisfaction	(35, 39)		(35)
**Technological**			
Proof of utility (by research)		(40)	(40)

*Green background means reported as facilitator. Orange means reported as both facilitator and barrier. [a] The numbers in parentheses denote the corresponding references. RC: Remote consultation, RM: Remote Monitoring, DCP: Digital Care Platform. The barrier or facilitator is reported when reported in less than three technologies, otherwise a barrier or facilitator is reported in the generic table (Table 2).

For peer review only

Discussion

This systematic umbrella review aimed to investigate the barriers and facilitators of digital health technology in patient care, as perceived by HCPs. Three types of digital health technologies were distinguished: 1) Remote consultation, 2) Remote monitoring, and 3) Digital care platforms. Barriers and facilitators from the perspective of HCP could be categorised as relating to 1) the organisation, 2) the HCPs themselves, 3) the patient, and 4) the technology. Overall, our review highlights that there are still major organisational requirements to be met in order to provide a solid foundation for digital health technology implementation. One way to overcome organisational barriers is by addressing them in the implementation strategy (see Figure 2). This includes adequate training and planning ahead for potential safety and confidentiality concerns. In addition, it also includes showing the HCP the eventual benefits such as improved health outcomes by the use of the digital health technology, and ensuring fully embedding within their clinical workflow. The results of this review indicate that the organisational facilitators as perceived by HCPs are comparable to the list of organisational barriers HCPs reported. Thus, these factors act as two sides of the same coin. Major facilitators to support a successful implementation strategy at the organisational level include deploying the right leadership and support, making use of change champions, and ensuring good working relationships. However, as healthcare will continue to face new challenges with regard to technology implementation, barriers and facilitators are not fixed entities that need to be resolved for successful implementation. Instead, the implementation of digital health technologies should be approached as a dynamic process which needs ongoing management and tailoring, taking social relations and context into account (65).

This umbrella review demonstrates that the majority of research in the past years on barriers and facilitators addressed common organisational barriers of technology implementation, such as lack of training, lack of time and resources, an increase in workload, and lack of leadership and support. This substantial attention to organisational issues can be interpreted in two ways. Firstly, that organisational barriers might remain prevalent for implementation, hence the substantial literature

For peer review only - http://bmjopen.bmj.com/site/about/guidelines.xhtml

on them. Alternatively, it suggests that organisational aspects could dominate discussions about implementation of technology, potentially overshadowing other levels on which barriers can take place. For instance, barriers on the level of the individual HCP, such as loss of communication, impeding the patient-physician relationship, undermining holistic surveillance or undermining aspects of professional identity, have been less frequently researched. For instance, a major barrier on the HCP level is loss of communication, specifically pertaining to remote consultation. As a result, HCPs experienced the possibility of misunderstanding the patient, and perceived a decreased personal connection with the patient (51, 55). In addition, HCPs perceived a difference in whether one sees the patient digitally for the first time, or whether one has already established a relationship with the patient. This distinction was particularly reported in reviews implementing digital health technologies during the Coronavirus pandemic, where it became more common to have an initial digital interaction instead of face-to-face consultation (51). In addition, technology could undermine aspects of holistic surveillance, as physical examinations are not possible and it is more difficult to pick up nonverbal cues (51, 58). Despite the HCP perceiving these barriers, patients were found to be satisfied with teleconsultation and remote monitoring and did not experience this loss in communication (51, 55, 58). In this regard, there is a discrepancy between the needs and perspectives of HCPs and patients. The most used explanation on differences in satisfaction levels with regard to video consultation is the inability of HCPs to physically examine the patient (66). While this review primarily focuses on the perspective of HCP, patients are also significant end users of technology. Therefore, it is essential to consider and identify the varying needs of patients in addition to those of HCPs, by conducting more research on the patients' perspectives on the influence of digital technology on their care, and the HCP-patient relationship specifically. Such research may yield more discrepancies, which need to be solved to provide patients with best care without unduly burdening HCP.

This review also indicates the importance of training of professionals at the start of an implementation trajectory. For transformative innovations such as the introduction of a new digital

For peer review only - http://bmjopen.bmj.com/site/about/guidelines.xhtml

health technology, adequate training to facilitate the user's adoption and use of the new technology is essential (67). However, the training needs of HCPs and ideal frequency of training are yet to be defined. Ongoing training, rather than a one-time instruction at the start of the implementation, may be more sustainable when considering potential staff changes (68). The training itself could vary, from practical instruction to hands-on learning, as reported in the study by Dugstad, Eide (69). At the onset, a theoretical approach to training of professionals was used by these authors, but this was soon replaced by practical instruction and training sessions which emphasised capacity building to acquire the necessary skills; such as using the app, handling swipe hand movements, and using software commands (69).

On the HCP level, major barriers included a loss of communication with patients, and the possibility of technology impeding the patient-HCP relationship. Patient connection is at the essence of being a HCP, and has become increasingly difficult in contemporary practice (70). A study conducted in fifty medical departments in the Netherlands showed that healthcare professionals with positive patient relationships were less likely to suffer from patient-related burnout even with high job demands (70). In addition, patient-related burnout was more prevalent among HCPs who had less positive patient relationships (71). In light of these findings, and with the increasing prevalence of burnout among HCPs (72), further research is necessary to investigate whether the use of digital health technology in healthcare actually results in a loss of gratifying interaction with patients.

Patient related barriers, as perceived by HCPs, related to questioning the ability of patients to use digital technology, patient anxiety for using the technology, and low literacy of patients. One of the actions of the *World Health Organisation* on their global strategy is that digital technologies should strengthen health equity by promoting an inclusive digital society (73). As digital health literacy requires skills complementary to health literacy, it is important to ensure that digital health technologies are available for all patients (74). Therefore, understanding and incorporating intended users' needs and context appears to be a prerequisite for effective use of digital health technology

For peer review only

(75), apparently also from the perspective of HCP. This could be achieved by co-creation, which is described as an interaction in which participants work together to create an outcome that is appreciated by both parties (76). By including HCPs in this process, the possibility that the digital health technologies will meet the needs and requirements of the end user in terms of content and usability will increase; this, in turn, may enhance technology implementation.

Strengths and limitations

The strengths of this study include its comprehensive search on digital health technologies, narrowed down to technologies that include patient interaction, its duplicate approach, and its focus on the end-user. However, the current review was limited by digital technology and policies regulating these being in a state of constant flux. Therefore, barriers and facilitators reported in older studies might be less relevant over time. However, given that half of the reviews included are less than five years old, we consider the publication date to be of minor influence. Another limitation is that some of the included reviews provided little context, making it difficult to determine whether findings were considered facilitators or barriers to technology implementation. In such instances, the reviewers either reached a consensus-based decision or reported a finding both as a barrier and facilitator. Finally, the included reviews differ in terms of the type of technology studied and the disease or condition in which the technology was used. The intention of this review was to provide as complete of an overview as possible of common barriers and facilitators as perceived by HCPs. All technologies in this review include a form of patient interaction, however, barriers or facilitators for a specific technology or implementation may exist.

Conclusions and implications for practice

The implementation of digital health technology in healthcare is influenced by several major barriers from the perspective of the HCP that need to be overcome. These include security and confidentiality issues, adequate training, HCPs unfamiliarity with technology, and HCPs questioning the ability of patients to use technology. In order to successfully implement digital health

For peer review only - http://bmjopen.bmj.com/site/about/guidelines.xhtml

technology, generic barriers and facilitators as well as barriers and facilitators per type of technology must be considered. Firstly, organisational barriers have been frequently reported and remain problematic. However, such barriers may act as facilitators when appropriately addressed in the implementation strategy. Secondly, barriers on both the HCP and patient levels are related to the patient-HCP relationship, the loss of communication, the undermining of holistic surveillance, and questioning the ability of patients. These barriers can be addressed by investigating the needs of the stakeholders involved and by including them as co-creators in the development of digital health technology. By involving HCPs in this process, the digital tool will be more likely to meet end-user requirements in terms of content and usability. In turn, this bolsters the chance for successful implementation. Thirdly, successful implementation of digital health technology demands time and resources. HCP training should be a continuous process to protect against issues related to staff turnover. Lastly, further research is necessary on the other end-user of digital health technology, the patient, in order to understand the role digital health technology occupies in the patient-HCP relationship, and to better understand the needs of this end-user.

Contributorship statement

SJO, KAZ, JJM, EMAS were involved with the design of this review. SJO, SPR, BSJC performed the screening of titles and abstracts of the included studies. With regard to data extraction and synthesis, this was performed by SJO, SPR, BSJC. SJO and SPR prepared the original draft of this review. JJM and EMAS contributed to the refinement. All authors have read and approved the final version.

Competing interests

No competing interest.

Funding statement

For peer review only - http://bmjopen.bmj.com/site/about/guidelines.xhtml

This research received no specific grant from any funding agency in the public, commercial or not-for-profit sectors.

Data sharing statement

All data relevant to the study are included in the article or uploaded as supplementary information

Ethics approval

Not applicable.

Patient consent for publication

Not applicable.

Acknowledgments

The authors wish to thank Dr. L.W.P. Peute, director from the eHealth living and learning lab at the Amsterdam University Medical Center, for her support regarding the tables and figures. Furthermore, the authors would like to express their gratitude to Rebecca Kerstens, from the University of South Australia, for critically assessing the manuscript.

For peer review only

References

1. Garavand A, Mohseni M, Asadi H, Etemadi M, Moradi-Joo M, Moosavi A. Factors influencing the adoption of health information technologies: a systematic review. Electronic physician. 2016;8(8):2713.

2. Kraus S, Schiavone F, Pluzhnikova A, Invernizzi AC. Digital transformation in healthcare: Analyzing the current state-of-research. Journal of Business Research. 2021;123:557-67.

3. Tsai CH, Eghdam A, Davoody N, Wright G, Flowerday S, Koch S. Effects of electronic health record implementation and barriers to adoption and use: a scoping review and qualitative analysis of the content. Life. 2020;10(12):327.

4. WHO. WHO guideline: recommendations on digital interventions for health system strengthening. Geneva: World Health Organization; 2019.

5. Smith C. What is wearable tech? Everything you need to know explained: Chris Smith; 2019 [Available from: https://www.wareable.com/wearable-tech/what-is-wearable-tech-753.

6. Tortorella GL, Fogliatto FS, Espôsto KF, Mac Cawley AF, Vassolo R, Tlapa D, Narayanamurthy G. Healthcare costs' reduction through the integration of Healthcare 4.0 technologies in developing economies. Total Quality Management & Business Excellence. 2020:1-21.

7. Shimbo D, Artinian NT, Basile JN, Krakoff LR, Margolis KL, Rakotz MK, et al. Self-measured blood pressure monitoring at home: a joint policy statement from the American Heart Association and American Medical Association. Circulation. 2020;142(4):e42-e63.

8. Hamine S, Gerth-Guyette E, Faulx D, Green BB, Ginsburg AS. Impact of mHealth chronic disease management on treatment adherence and patient outcomes: a systematic review. Journal of medical Internet research. 2015;17(2):e52.

9. Seoane F, Traver V, Hazelzet J. Value-Driven Digital Transformation in Health and Medical Care. Interactive Process Mining in Healthcare: Springer; 2021. p. 13-26.

10. Crespo-Almendros E, Del Barrio-García S. Online airline ticket purchasing: Influence of online sales promotion type and Internet experience. Journal of Air Transport Management. 2016;53:23-34.

11. Olesch A. Digialisation: a missing connectior or health systems in Europe? Eurohealth. 2022;28(3):24-8.

12. Gopal G, Suter-Crazzolara C, Toldo L, Eberhardt W. Digital transformation in healthcare–architectures of present and future information technologies. Clinical Chemistry and Laboratory Medicine (CCLM). 2019;57(3):328-35.

13. van Gemert-Pijnen LJ, editor Digitalisering in de gezondheidszorg nader beschouwd: eindrapport VWS MEVA. Digitalisering nader beschouwd, opdracht van VWS MEVA; 2022.

For peer review only - http://bmjopen.bmj.com/site/about/guidelines.xhtml

14. Manogaran G, Lopez D, Thota C, Abbas KM, Pyne S, Sundarasekar R. Big data analytics in healthcare Internet of Things. Innovative healthcare systems for the 21st century: Springer; 2017. p. 263-84.

15. Smuck M, Odonkor CA, Wilt JK, Schmidt N, Swiernik MA. The emerging clinical role of wearables: factors for successful implementation in healthcare. NPJ Digital Medicine. 2021;4(1):1-8.

16. Yen P-Y, Bakken S. Review of health information technology usability study methodologies. Journal of the American Medical Informatics Association. 2012;19(3):413-22.

17. Shahbaz M, Gao C, Zhai L, Shahzad F, Hu Y. Investigating the adoption of big data analytics in healthcare: the moderating role of resistance to change. Journal of Big Data. 2019;6(1):1-20.

18. Smoll NR, Walker J, Khandaker G. The barriers and enablers to downloading the COVIDSafe app–a topic modelling analysis. Australian and New Zealand Journal of Public Health. 2021;45(4):344-7.

19. Sangers T, Wakkee M, Kramer-Noels E, Nijsten T, Lugtenberg M. Views on mobile health apps for skin cancer screening in the general population: an in-depth qualitative exploration of perceived barriers and facilitators. British Journal of Dermatology. 2021;185(5):961-9.

20. Slevin P, Kessie T, Cullen J, Butler M, Donnelly S, Caulfield B. Exploring the barriers and facilitators for the use of digital health technologies for the management of COPD: a qualitative study of clinician perceptions. QJM: An International Journal of Medicine. 2020;113(3):163-72.

21. Foong HF, Kyaw BM, Upton Z, Tudor Car L. Facilitators and barriers of using digital technology for the management of diabetic foot ulcers: A qualitative systematic review. International wound journal. 2020;17(5):1266-81.

22. Moher D, Liberati A, Tetzlaff J, Altman DG, Group* P. Preferred reporting items for systematic reviews and meta-analyses: the PRISMA statement. Annals of internal medicine. 2009;151(4):264-9.

23. Rabin BA, Brownson RC. Terminology for dissemination and implementation research2017. 19-45 p.

24. Nilsen P. Making sense of implementation theories, models, and frameworks. Implementation Science 30: Springer; 2020. p. 53-79.

25. Liberati A, Altman DG, Tetzlaff J, Mulrow C, Gøtzsche PC, Ioannidis JP, et al. The PRISMA statement for reporting systematic reviews and meta-analyses of studies that evaluate health care interventions: explanation and elaboration. Journal of clinical epidemiology. 2009;62(10):e1-e34.

26. Hair K, Bahor Z, Macleod M, Liao J, Sena ES. The automated systematic search Deduplicator (ASySD): a rapid, open-source, interoperable tool to remove duplicate citations in biomedical systematic reviews. Biorxiv. 2021.

For peer review only - http://bmjopen.bmj.com/site/about/guidelines.xhtml

27. Ouzzani M, Hammady H, Fedorowicz Z, Elmagarmid A. Rayyan-a web and mobile app for systematic reviews. Systematic Reviews, 5, 210. 2016.

28. Richardson E, Aissat D, Williams GA, Fahy N. Keeping what works: remote consultations during the COVID-19 pandemic. Eurohealth. 2020;26(2):73-6.

29. Vegesna A, Tran M, Angelaccio M, Arcona S. Remote patient monitoring via non-invasive digital technologies: a systematic review. Telemedicine and e-Health. 2017;23(1):3-17.

30. Fürstenau D, Auschra C, Klein S, Gersch M. A process perspective on platform design and management: evidence from a digital platform in health care. Electronic Markets. 2019;29(4):581-96.

31. Aromataris E, Fernandez R, Godfrey CM, Holly C, Khalil H, Tungpunkom P. Summarizing systematic reviews: methodological development, conduct and reporting of an umbrella review approach. JBI Evidence Implementation. 2015;13(3):132-40.

32. Antoun J. Electronic mail communication between physicians and patients: a review of challenges and opportunities. Fam Pract. 2016;33(2):121-6.

33. Hassibian MR, Hassibian S. Telemedicine acceptance and implementation in developing countries: Benefits, categories, and barriers. Razavi International Journal of Medicine. 2016;4(3).

34. Almathami HKY, Win KT, Vlahu-Gjorgievska E. Barriers and Facilitators That Influence Telemedicine-Based, Real-Time, Online Consultation at Patients' Homes: Systematic Literature Review. J Med Internet Res. 2020;22(2):e16407.

35. Appleton R, Williams J, San Juan NV, Needle JJ, Schlief M, Jordan H, et al. Implementation, adoption, and perceptions of telemental health during the COVID-19 pandemic: systematic review. Journal of medical Internet research. 2021;23(12):e31746.

36. Batterham PJ, Sunderland M, Calear AL, Davey CG, Christensen H, Teesson M, et al. Developing a roadmap for the translation of e-mental health services for depression. Aust N Z J Psychiatry. 2015;49(9):776-84.

37. Bokolo AJ. Exploring the adoption of telemedicine and virtual software for care of outpatients during and after COVID-19 pandemic. Ir J Med Sci. 2021;190(1):1-10.

38. Brunton L, Bower P, Sanders C. The Contradictions of Telehealth User Experience in Chronic Obstructive Pulmonary Disease (COPD): A Qualitative Meta-Synthesis. PLoS One. 2015;10(10):e0139561.

39. Cowan KE, McKean AJ, Gentry MT, Hilty DM. Barriers to Use of Telepsychiatry: Clinicians as Gatekeepers. Mayo Clinic Proceedings. 2019;94(12):2510-23.

40. de Grood C, Raissi A, Kwon Y, Santana MJ. Adoption of e-health technology by physicians: a scoping review. J Multidiscip Healthc. 2016;9:335-44.

For peer review only

For peer review only - http://bmjopen.bmj.com/site/about/guidelines.xhtml

41. De Guzman KR, Snoswell CL, Taylor ML, Senanayake B, Haydon HM, Batch JA, et al. A Systematic Review of Pediatric Telediabetes Service Models. Diabetes Technol Ther. 2020;22(8):623-38.

42. Finkelstein J, Knight A, Marinopoulos S, Gibbons MC, Berger Z, Aboumatar H, et al. Enabling patient-centered care through health information technology. Evid Rep Technol Assess (Full Rep). 2012(206):1-1531.

43. Gagnon MP, Ngangue P, Payne-Gagnon J, Desmartis M. m-Health adoption by healthcare professionals: a systematic review. Journal of the American Medical Informatics Association. 2016;23(1):212-20.

44. Greenhalgh T, A'Court C, Shaw S. Understanding heart failure; explaining telehealth - a hermeneutic systematic review. BMC Cardiovasc Disord. 2017;17(1):156.

45. Hopstaken JS, Verweij L, van Laarhoven CJ, Blijlevens NM, Stommel MW, Hermens RP. Effect of digital care platforms on quality of care for oncological patients and barriers and facilitators for their implementation: Systematic review. Journal of Medical Internet Research. 2021;23(9):e28869.

46. Jacob C, Sanchez-Vazquez A, Ivory C. Social, Organizational, and Technological Factors Impacting Clinicians' Adoption of Mobile Health Tools: Systematic Literature Review. Jmir Mhealth and Uhealth. 2020;8(2).

47. Lewinski AA, Rushton S, Van Voorhees E, Boggan JC, Whited JD, Shoup JP, et al. Implementing remote triage in large health systems: A qualitative evidence synthesis. Res Nurs Health. 2021;44(1):138-54.

48. Li J, Talaei-Khoei A, Seale H, Ray P, Macintyre CR. Health Care Provider Adoption of eHealth: Systematic Literature Review. Interact J Med Res. 2013;2(1):e7.

49. Liyanage-Don N, Fung D, Phillips E, Kronish IM. Implementing Home Blood Pressure Monitoring into Clinical Practice. Current Hypertension Reports. 2019;21(2).

50. Maclure K, Stewart D, Strath A. A systematic review of medical and non-medical practitioners' views of the impact of ehealth on shared care. European Journal of Hospital Pharmacy. 2014;21(1):54-62.

51. Meng G, McAiney C, Perlman CM, McKillop I, Tisseverasinghe T, Chen HH. Service process factors affecting patients' and clinicians' experiences on rapid teleconsultation implementation in out-patient neurology services during COVID-19 pandemic: a scoping review. BMC health services research. 2022;22(1):1-17.

52. Metzger G, Jatana K, Apfeld J, Deans KJ, Minneci PC, Halaweish I. State of telemedicine use in pediatric surgery in the USA-where we stand and what we can gain from the COVID-19 pandemic: A scoping review. World Journal of Pediatric Surgery. 2021;4(1).

For peer review only - http://bmjopen.bmj.com/site/about/guidelines.xhtml

53. Mileski M, Kruse CS, Catalani J, Haderer T. Adopting Telemedicine for the Self-Management of Hypertension: Systematic Review. JMIR Med Inform. 2017;5(4):e41.

54. Muir SD, de Boer K, Nedeljkovic M, Meyer D. Barriers and facilitators of videoconferencing psychotherapy implementation in veteran mental health care environments: a systematic review. BMC Health Serv Res. 2020;20(1):999.

55. O'Cathail M, Sivanandan MA, Diver C, Patel P, Christian J. The Use of Patient-Facing Teleconsultations in the National Health Service: Scoping Review. JMIR Med Inform. 2020;8(3):e15380.

56. Palacholla RS, Fischer N, Coleman A, Agboola S, Kirley K, Felsted J, et al. Provider- and Patient-Related Barriers to and Facilitators of Digital Health Technology Adoption for Hypertension Management: Scoping Review. JMIR Cardio. 2019;3(1):e11951.

57. Slater H, Campbell JM, Stinson JN, Burley MM, Briggs AM. End User and Implementer Experiences of mHealth Technologies for Noncommunicable Chronic Disease Management in Young Adults: Systematic Review. J Med Internet Res. 2017;19(12):e406.

58. Smith SM, Jacobsen JHW, Atlas AP, Khoja A, Kovoor JG, Tivey DR, et al. Telehealth in surgery: an umbrella review. ANZ journal of surgery. 2021;91(11):2360-75.

59. Varsi C, Solberg Nes L, Kristjansdottir OB, Kelders SM, Stenberg U, Zangi HA, et al. Implementation Strategies to Enhance the Implementation of eHealth Programs for Patients With Chronic Illnesses: Realist Systematic Review. J Med Internet Res. 2019;21(9):e14255.

60. Vedel I, Akhlaghpour S, Vaghefi I, Bergman H, Lapointe L. Health information technologies in geriatrics and gerontology: a mixed systematic review. Journal of the American Medical Informatics Association. 2013;20(6):1109-19.

61. Vyas KS, Hambrick HR, Shakir A, Morrison SD, Tran DC, Pearson K, et al. A Systematic Review of the Use of Telemedicine in Plastic and Reconstructive Surgery and Dermatology. Ann Plast Surg. 2017;78(6):736-68.

62. Whitelaw S, Pellegrini DM, Mamas MA, Cowie M, Van Spall HGC. Barriers and facilitators of the uptake of digital health technology in cardiovascular care: a systematic scoping review. Eur Heart J Digit Health. 2021;2(1):62-74.

63. Xyrichis A, Iliopoulou K, Mackintosh NJ, Bench S, Terblanche M, Philippou J, Sandall J. Healthcare stakeholders' perceptions and experiences of factors affecting the implementation of critical care telemedicine (CCT): qualitative evidence synthesis. Cochrane Database Syst Rev. 2021;2(2):Cd012876.

For peer review only

For peer review only - http://bmjopen.bmj.com/site/about/guidelines.xhtml

64. Zaman SB, Khan RK, Evans RG, Thrift AG, Maddison R, Islam SMS. Exploring Barriers to and Enablers of the Adoption of Information and Communication Technology for the Care of Older Adults With Chronic Diseases: Scoping Review. JMIR aging. 2022;5(1):e25251.

65. Checkland K, Harrison S, Marshall M. Is the metaphor of'barriers to change'useful in understanding implementation? Evidence from general medical practice. Journal of health services research & policy. 2007;12(2):95-100.

66. Zandbelt LC, de Kanter FEC, Ubbink DT. E-consulting in a medical specialist setting: Medicine of the future? Patient Education and Counseling. 2016;99(5):689-705.

67. Gallouj F, Weinstein O. Innovation in services. Research policy. 1997;26(4-5):537-56.

68. Versluis A, van Luenen S, Meijer E, Honkoop PJ, Pinnock H, Mohr DC, et al. SERIES: eHealth in primary care. Part 4: addressing the challenges of implementation. European Journal of General Practice. 2020;26(1):140-5.

69. Dugstad J, Eide T, Nilsen ER, Eide H. Towards successful digital transformation through co-creation: a longitudinal study of a four-year implementation of digital monitoring technology in residential care for persons with dementia. BMC Health Serv Res. 2019;19(1):366.

70. Scheepers R, Silkens M, van den Berg J, Lombarts K. Associations between job demands, job resources and patient-related burnout among physicians: results from a multicentre observational study. BMJ Open. 2020;10(9):e038466.

71. Shanafelt TD. Enhancing meaning in work: a prescription for preventing physician burnout and promoting patient-centered care. Jama. 2009;302(12):1338-40.

72. Hiver C, Villa A, Bellagamba G, Lehucher-Michel MP. Burnout prevalence among European physicians: a systematic review and meta-analysis. Int Arch Occup Environ Health. 2022;95(1):259-73.

73. Organization WH. Global strategy on digital health 2020-2025. 2021.

74. Smith B, Magnani JW. New technologies, new disparities: The intersection of electronic health and digital health literacy. International Journal of Cardiology. 2019;292:280-2.

75. Thies K, Anderson D, Cramer B. Lack of Adoption of a Mobile App to Support Patient Self-Management of Diabetes and Hypertension in a Federally Qualified Health Center: Interview Analysis of Staff and Patients in a Failed Randomized Trial. JMIR Hum Factors. 2017;4(4):e24.

76. Prahalad CK, Ramaswamy V. Co-creation experiences: The next practice in value creation. Journal of interactive marketing. 2004;18(3):5-14.

For peer review only - http://bmjopen.bmj.com/site/about/guidelines.xhtml

Figure Legend

Figure 1 - Flowchart of the article selection.

Figure 2 - Top three barriers or facilitators on each level of digital health implementation, and practical implications or solutions.

For peer review only

For peer review only - http://bmjopen.bmj.com/site/about/guidelines.xhtml

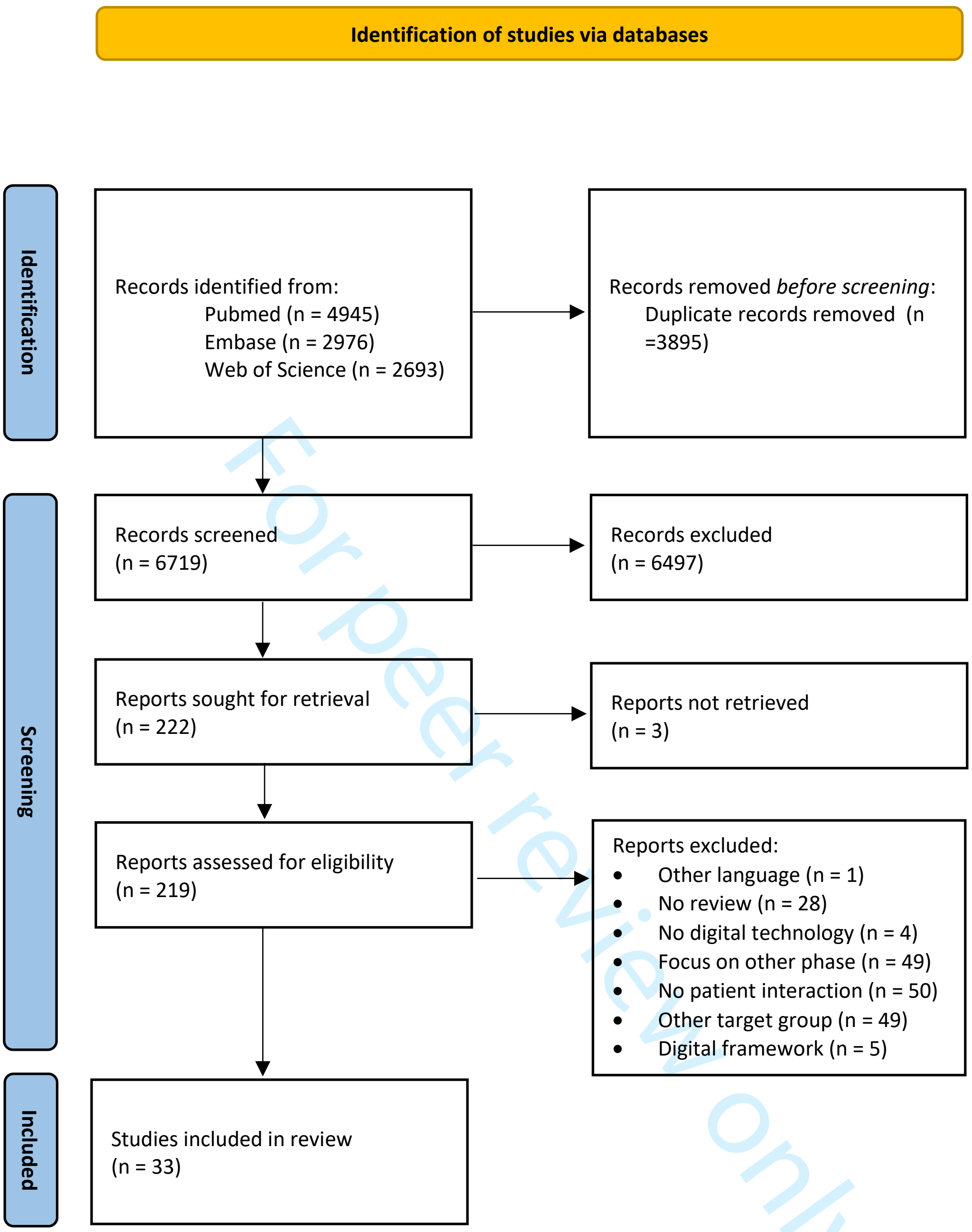

Figure 1. Flowchart of the article selection

For peer review only

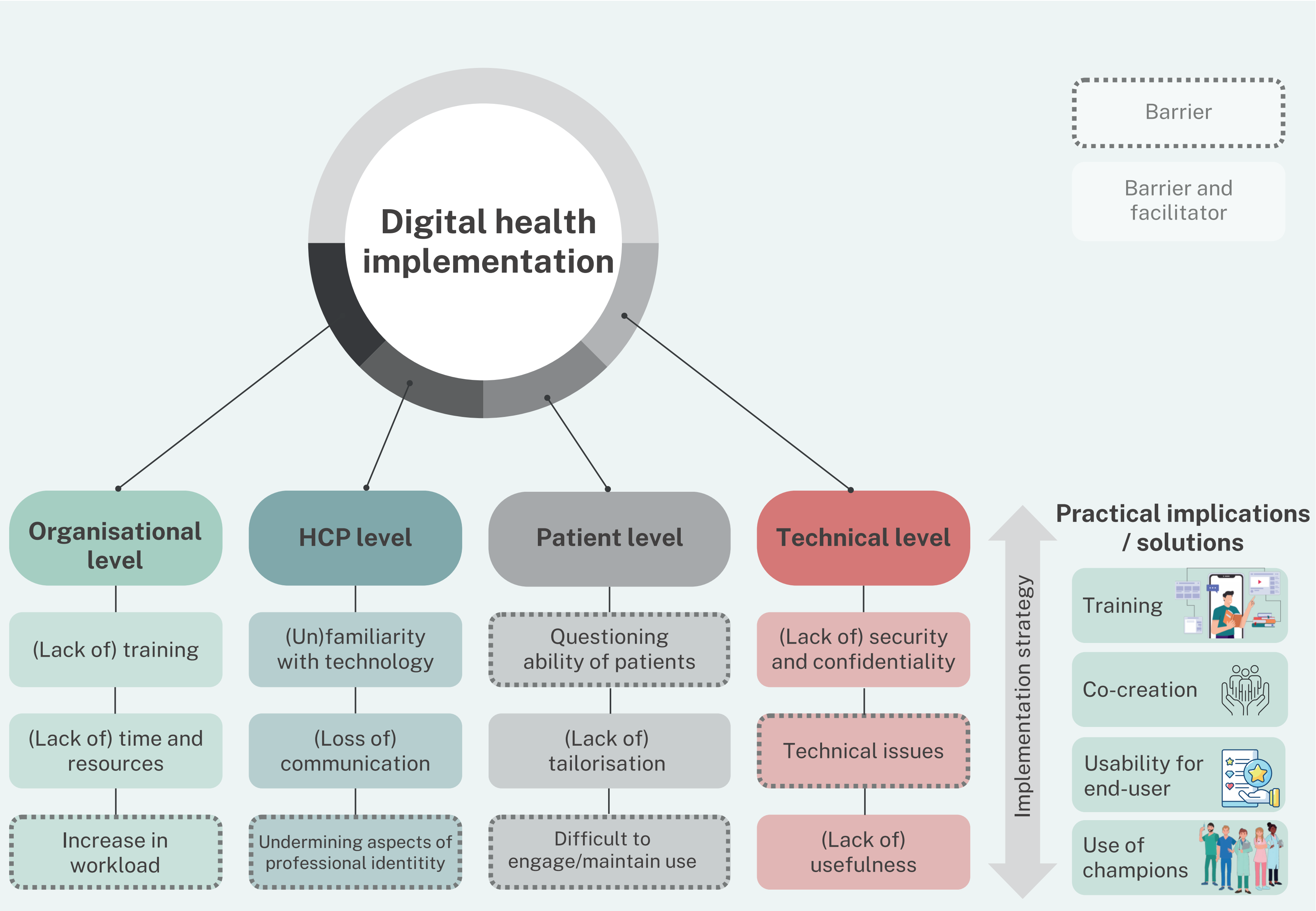
Digital health implementation
Barrier
Barrier and facilitator
Organisational level
HCP level
Patient level
Technical level
(Lack of) training
(Lack of) time and resources
Increase in workload
(Un)familiarity with technology
(Loss of) communication
Undermining aspects of professional identitity
Questioning ability of patients
(Lack of) tailorisation
Difficult to engage/maintain use
(Lack of) security and confidentiality
Technical issues
(Lack of) usefulness
Implementation strategy
Practical implications / solutions
Training
Co-creation
Usability for end-user
Use of champions

Supplementary material

Supplementary Table 1. In- and exclusion criteria

Inclusion criteria	Exclusion criteria
• Publication type: systematic review, meta-analysis, literature review. At least when systematic review was mentioned in title or abstract.<br>• Describing barriers, facilitators, successes, inhibitions, failures for the adoption, implementation, maintenance of technology in practice.<br>• From the healthcare professional perspective: medical specialists, nurse practitioners, and physician assistants.	• Articles written in other language than Dutch, German, or English<br>• Publication type: research protocol, empirical studies, opinion papers, conference abstract.<br>• No digital technology described<br>• Focus on other phase than implementation, adoption, maintenance phase of digital technology<br>• No patient interaction with the technology<br>• Different target group (managerial, patient, primary care, nurse).<br>• Article describes a framework related to technology implementation<br>• No barriers or facilitators described

For peer review only

For peer review only - http://bmjopen.bmj.com/site/about/guidelines.xhtml

Supplementary Table 2. PICO with original PubMed search and results (and translated to Embase and Web of Science)

PICO*	Participants	Intervention	Context	Outcome
Research question	Medical specialists, nurse practitioners, physician assistants involved with digital technology	Digital technology based on patient interaction	Implementation, adoption, maintenance of technology in healthcare	Barriers and facilitators
PubMed	"Physicians"[Mesh] OR "Physician Assistants"[Mesh] OR "Nurse Practitioners"[Mesh] "Attitude of health personnel"[Mesh] OR "Allergist*"[tiab] OR "Anesthesiologist*"[tiab] OR "Anaesthesiologist*"[tiab] OR "Cardiologist*"[tiab] OR "Dermatologist*"[tiab] OR "Doctor*"[tiab] OR "Endocrinologist*"[tiab] OR "Gastroenterologist*"[tiab] OR "Geriatrician*"[tiab] OR "Geriatrist*"[tiab] OR "Gerontologist*"[tiab] OR "Gynaecologist*" OR "Gynecologist*"[tiab] OR "Hematologist*"[tiab] OR "Hepatologist*"[tiab] OR "Hospitalist*"[tiab] OR "Immunologist*"[tiab] OR "Infectious disease specialist*"[tiab] OR "Internist*"[tiab] OR "Intensivist*"[tiab] OR "Medical geneticist*"[tiab] OR "Medical microbiologist*"[tiab] OR "Medical specialist*"[tiab] OR "Nephrologist*"[tiab] OR "Neurologist*"[tiab] OR "Obstetrician*"[tiab] OR "Oncologist*"[tiab] OR "Ophthalmologist*"[tiab] OR "Orthopedic specialist*"[tiab] OR "Otolaryngologist*"[tiab] OR "Pathologist*"[tiab] OR "Paediatrician*"[tiab] OR "Pediatrician*"[tiab] OR "Physician*"[tiab] OR "Neonatologist*"[tiab] OR "Neurosurgeon*"[tiab] OR "Pathologist*"[tiab] OR "Physiatrist*"[tiab] OR "Psychiatrist*"[tiab] OR "Physician*"[tiab] OR "Pulmonologist*"[tiab] OR "Radiologist*"[tiab] OR "Radiotherapist*"[tiab] OR "Rheumatologist*"[tiab] OR "Surgeon*"[tiab] OR "Neurosurgeon*"[tiab] OR "Urologist*"[tiab] OR "nurse practitioner*"[tiab]	"Telemedicine"[Mesh] OR "User-Computer Interface"[Mesh] OR "Multimedia"[Mesh] OR "Cell phones"[Mesh] OR "Public health informatics"[Mesh] OR "Medical informatics"[Mesh] OR "Computers, handheld"[Mesh] OR "Mobile Applications"[Mesh] OR "Internet"[Mesh] OR "Patient Portals"[Mesh] OR "econsult*"[tiab] OR "e-consult*"[tiab] OR "ediagnos*"[tiab] OR "e diagnos*"[tiab] OR "mobile health*"[tiab] OR "mhealth*"[tiab] OR "m health*"[tiab] OR "telehealth*"[tiab] OR "tele health"[tiab] OR "remote consult*"[tiab] OR "teleconsult*"[tiab] OR "tele consult*"[tiab] OR "telediagnos*"[tiab] OR "tele diagnos*"[tiab] OR "telemedic*"[tiab] OR "tele medic*"[tiab] OR "telemonitor*"[tiab] OR "tele monitor*"[tiab] OR "ehealth*"[tiab] OR "e-health*"[tiab] OR "telecare"[tiab] OR "tele care"[tiab] OR "digital health"[tiab] OR "app"[tiab] OR "apps"[tiab] OR "smartphone*"[tiab] OR "phone application*"[tiab] OR "telephone application*"[tiab] OR "mobile application*"[tiab] OR "mobile technolog*"[tiab] OR "health technolog*"[tiab] OR "health application*"[tiab] OR "internet*"[tiab] OR "world wide web*"[tiab] OR "webportal*"[tiab] OR "web portal*"[tiab] OR "patient portal*"[tiab] OR "ipad"[tiab] OR "ipads"[tiab] OR "telecounsel*"[tiab] OR "tele counsel*"[tiab] OR "remote counsel*"[tiab] OR "distance consult*"[tiab] OR "distance counsel*"[tiab] OR "distant consult*"[tiab] OR "patient monitoring"[tiab] OR "interactive voice response*"[tiab] OR "multimedia"[tiab] OR "Mhapps"[tiab] OR "e-coach*"[tiab] OR "wearable*"[tiab] OR "handheld"[tiab] OR "virtual patient*"[tiab] OR "computer"[tiab] OR "digital*"[tiab]	"Organisational Innovation"[Mesh] OR "organisational chang*"[tiab] OR "organisational chang*"[tiab] OR "organisational innovat*"[tiab] OR "organisational innovat*"[tiab] OR "implement*"[tiab] OR "adopt*"[tiab] OR "transform*"[tiab]	No search terms included

* The C of Comparison is replaced to Context, since we did not look for studies with controlled conditions (placebo/no digital technology). Search terms between columns were combined with AND; the search is limited to publication type [systematic reviews, literature reviews and meta-analysis].

For peer review only - http://bmjopen.bmj.com/site/about/guidelines.xhtml

Supplementary Table 2. PubMed Results (17 June 2022)

Search	PubMed Query – June 17, 2022	Results
#5	#1 AND #2 AND #3 AND #4	4,945
#4	(("Review Literature as Topic"[Mesh] OR "Review"[Publication Type] OR "Meta-Analysis as Topic"[Mesh] OR "review*"[tiab] OR "meta-analys*"[tiab] OR "Meta-Analysis "[Publication Type] OR systematic[sb]) NOT ("Letter"[Publication Type] OR "Editorial"[Publication Type] OR "Comment"[Publication Type]))	4,149,865
#3	"Organizational Innovation"[Mesh] OR "organizational chang*"[tiab] OR "organisational chang*"[tiab] OR "organizational innovat*"[tiab] OR "organisational innovat*"[tiab] OR "implement*"[tiab] OR "adopt*"[tiab] OR "transform*"[tiab]	1,458,582
#2	"Physicians"[Mesh] OR "Physician Assistants"[Mesh] OR "Nurse Practitioners"[Mesh] "Attitude of health personnel"[Mesh] OR "Allergist*"[tiab] OR "Anesthesiologist*"[tiab] OR "Anaesthesiologist*"[tiab] OR "Cardiologist*"[tiab] OR "Dermatologist*"[tiab] OR "Doctor*"[tiab] OR "Endocrinologist*"[tiab] OR "Gastroenterologist*"[tiab] OR "Geriatrician*"[tiab] OR "Geriatrist*"[tiab] OR "Gerontologist*"[tiab] OR "Gynaecologist*" OR "Gynecologist*"[tiab] OR "Hematologist*"[tiab] OR "Hepatologist*"[tiab] OR "Hospitalist*"[tiab] OR "Immunologist*"[tiab] OR "Infectious disease specialist*"[tiab] OR "Internist*"[tiab] OR "Intensivist*"[tiab] OR "Medical geneticist*"[tiab] OR "Medical microbiologist*"[tiab] OR "Medical specialist*"[tiab] OR "Nephrologist*"[tiab] OR "Neurologist*"[tiab] OR "Obstetrician*"[tiab] OR "Oncologist*"[tiab] OR "Ophthalmologist*"[tiab] OR "Orthopedic specialist*"[tiab] OR "Otolaryngologist*"[tiab] OR "Pathologist*"[tiab] OR "Paediatrician*"[tiab] OR "Pediatrician*"[tiab] OR "Physician*"[tiab] OR "Neonatologist*"[tiab] OR "Neurosurgeon*"[tiab] OR "Pathologist*"[tiab] OR "Physiatrist*"[tiab] OR "Psychiatrist*"[tiab] OR "Physician*"[tiab] OR "Pulmonologist*"[tiab] OR "Radiologist*"[tiab] OR "Radiotherapist*"[tiab] OR "Rheumatologist*"[tiab] OR "Surgeon*"[tiab] OR "Neurosurgeon*"[tiab] OR "Urologist*"[tiab] OR "nurse practitioner*"[tiab] OR "clinician*"[tiab]	1,297,435
#1	"Telemedicine"[Mesh] OR "User-Computer Interface"[Mesh] OR "Multimedia"[Mesh] OR "Cell phones"[Mesh] OR "Public health informatics"[Mesh] OR "Medical informatics"[Mesh] OR "Nursing informatics"[Mesh] OR "Computers, handheld"[Mesh] OR "Mobile Applications"[Mesh] OR "Internet"[Mesh] OR "Patient Portals"[Mesh] OR "econsult*"[tiab] OR "e-consult*"[tiab] OR "ediagnos*"[tiab] OR "e diagnos*"[tiab] OR "mobile health*"[tiab] OR "mhealth*"[tiab] OR "m health*"[tiab] OR "telehealth*"[tiab] OR "tele health"[tiab] OR "remote consult*"[tiab] OR "teleconsult*"[tiab] OR "tele consult*"[tiab] OR "telediagnos*"[tiab] OR "tele diagnos*"[tiab] OR "telemedic*"[tiab] OR "tele medic*"[tiab] OR "telemonitor*"[tiab] OR "tele monitor*"[tiab] OR "ehealth*"[tiab] OR "e-health*"[tiab] OR "telecare"[tiab] OR "tele care"[tiab] OR "digital health"[tiab] OR "app"[tiab] OR "apps"[tiab] OR "smartphone*"[tiab] OR "phone application*"[tiab] OR "telephone application*"[tiab] OR "mobile application*"[tiab] OR "mobile technolog*"[tiab] OR "health technolog*"[tiab] OR "health application*"[tiab] OR "internet*"[tiab] OR "world wide web*"[tiab] OR "webportal*"[tiab] OR "web portal*"[tiab] OR "patient portal*"[tiab] OR "ipad"[tiab] OR "ipads"[tiab] OR "telecounsel*"[tiab] OR "tele counsel*"[tiab] OR "remote counsel*"[tiab] OR "distance consult*"[tiab] OR "distance counsel*"[tiab] OR "distant consult*"[tiab] OR "patient monitoring"[tiab] OR "interactive voice response*"[tiab] OR "multimedia"[tiab] OR "Mhapps"[tiab] OR "e-coach*"[tiab] OR "wearable*"[tiab] OR "handheld"[tiab] OR "virtual patient*"[tiab] OR "computer"[tiab] OR "digital*"[tiab]	1,098,834

For peer review only - http://bmjopen.bmj.com/site/about/guidelines.xhtml

Supplementary Table 2. Embase.com Results (17 June 2022)

Search	Embase Query – June 17, 2022	Results
#5	#1 AND #2 AND #3 AND #4	2,976
#4	('systematic review'/exp OR 'meta analysis'/exp OR 'review*':ab,ti,kw OR 'meta-analys*':ab,ti,kw) NOT ('conference abstract'/it OR 'conference review'/it OR 'editorial'/it OR 'erratum'/it OR 'letter'/it OR 'note'/it OR 'short survey'/it)	2,739,615
#3	'organizational chang*':ti,ab,kw OR 'organisational chang*':ti,ab,kw OR 'organizational innovat*':ti,ab,kw OR 'organisational innovat*':ti,ab,kw OR 'implement*':ti,ab,kw OR 'adopt*':ti,ab,kw OR 'transform*':ti,ab,kw	1,776,391
#2	'physician'/exp OR 'physician assistant'/exp OR 'nurse practitioner'/exp OR 'allergist*':ti,ab,kw OR 'anesthesiologist*':ti,ab,kw OR 'anaesthesiologist*':ti,ab,kw OR 'cardiologist*':ti,ab,kw OR 'dermatologist*':ti,ab,kw OR 'doctor*':ti,ab,kw OR 'endocrinologist*':ti,ab,kw OR 'gastroenterologist*':ti,ab,kw OR 'geriatrician*':ti,ab,kw OR 'geriatrist*':ti,ab,kw OR 'gerontologist*':ti,ab,kw OR 'gynaecologist*':ti,ab,kw OR 'gynecologist*':ti,ab,kw OR 'hematologist*':ti,ab,kw OR 'hepatologist*':ti,ab,kw OR 'hospitalist*':ti,ab,kw OR 'immunologist*':ti,ab,kw OR 'infectious disease specialist*':ti,ab,kw OR 'internist*':ti,ab,kw OR 'intensivist*':ti,ab,kw OR 'medical geneticist*':ti,ab,kw OR 'medical microbiologist*':ti,ab,kw OR 'medical specialist*':ti,ab,kw OR 'nephrologist*':ti,ab,kw OR 'neurologist*':ti,ab,kw OR 'obstetrician*':ti,ab,kw OR 'oncologist*':ti,ab,kw OR 'ophthalmologist*':ti,ab,kw OR 'orthopedic specialist*':ti,ab,kw OR 'otolaryngologist*':ti,ab,kw OR 'paediatrician*':ti,ab,kw OR 'pediatrician*':ti,ab,kw OR 'neonatologist*':ti,ab,kw OR 'pathologist*':ti,ab,kw OR 'physiatrist*':ti,ab,kw OR 'psychiatrist*':ti,ab,kw OR 'physician*':ti,ab,kw OR 'pulmonologist*':ti,ab,kw OR 'radiologist*':ti,ab,kw OR 'radiotherapist*':ti,ab,kw OR 'rheumatologist*':ti,ab,kw OR 'surgeon*':ti,ab,kw OR 'neurosurgeon*':ti,ab,kw OR 'urologist*':ti,ab,kw OR 'nurse practitioner*':ti,ab,kw OR 'clinician*':ti,ab,kw	2,201,667
#1	'telehealth'/de OR 'telemedicine'/de OR 'telemonitoring'/exp OR 'internet'/exp OR 'computer interface'/exp OR 'multimedia'/exp OR 'mobile phone'/exp OR 'microcomputer'/exp OR 'mobile application'/exp OR 'medical informatics'/exp OR 'econsult*':ti,ab,kw OR 'e-consult*':ti,ab,kw OR 'ediagnos*':ti,ab,kw OR 'e diagnos*':ti,ab,kw OR 'mobile health*':ti,ab,kw OR 'mhealth*':ti,ab,kw OR 'm health*':ti,ab,kw OR 'telehealth*':ti,ab,kw OR 'tele health':ti,ab,kw OR 'remote consult*':ti,ab,kw OR 'teleconsult*':ti,ab,kw OR 'tele consult*':ti,ab,kw OR 'telediagnos*':ti,ab,kw OR 'tele diagnos*':ti,ab,kw OR 'telemedic*':ti,ab,kw OR 'tele medic*':ti,ab,kw OR 'telemonitor*':ti,ab,kw OR 'tele monitor*':ti,ab,kw OR 'ehealth*':ti,ab,kw OR 'e-health*':ti,ab,kw OR 'telecare':ti,ab,kw OR 'tele care':ti,ab,kw OR 'digital health':ti,ab,kw OR 'app':ti,ab,kw OR 'apps':ti,ab,kw OR 'smartphone*':ti,ab,kw OR 'phone application*':ti,ab,kw OR 'telephone application*':ti,ab,kw OR 'mobile application*':ti,ab,kw OR 'mobile technolog*':ti,ab,kw OR 'health technolog*':ti,ab,kw OR 'health application*':ti,ab,kw OR 'internet*':ti,ab,kw OR 'world wide web*':ti,ab,kw OR 'webportal*':ti,ab,kw OR 'web portal*':ti,ab,kw OR 'patient portal*':ti,ab,kw OR 'ipad':ti,ab,kw OR 'ipads':ti,ab,kw OR 'telecounsel*':ti,ab,kw OR 'tele counsel*':ti,ab,kw OR 'remote counsel*':ti,ab,kw OR 'distance consult*':ti,ab,kw OR 'distance counsel*':ti,ab,kw OR 'distant consult*':ti,ab,kw OR 'patient monitoring':ti,ab,kw OR 'interactive voice response*':ti,ab,kw OR 'multimedia':ti,ab,kw OR 'mhapps':ti,ab,kw OR 'e-coach*':ti,ab,kw OR 'wearable*':ti,ab,kw OR 'handheld':ti,ab,kw OR 'virtual patient*':ti,ab,kw OR 'computer':ti,ab,kw OR 'digital*':ti,ab,kw	889,047

For peer review only

Supplementary Table 2. Clarivate Analytics/Web of Science Core Collection Results (17 June 2022)

Search	Web of Science Query – June 17, 2022	Results
#5	#1 AND #2 AND #3 AND #4	2,693
#4	TS=("review*" OR "meta-analys*")	3,174,557
#3	TS=("organizational chang*" OR "organisational chang*" OR "organizational innovat*" OR "organisational innovat*" OR "implement*" OR "adopt*" OR "transform*")	3,965,854
#2	TS=("Allergist*" OR "Anesthesiologist*" OR "Anaesthesiologist*" OR "Cardiologist*" OR "Dermatologist*" OR "Doctor*" OR "Endocrinologist*" OR "Gastroenterologist*" OR "Geriatrician*" OR "Geriatrist*" OR "Gerontologist*" OR "Gynaecologist*" OR "Gynecologist*" OR "Hematologist*" OR "Hepatologist*" OR "Hospitalist*" OR "Immunologist*" OR "Infectious disease specialist*" OR "Internist*" OR "Intensivist*" OR "Medical geneticist*" OR "Medical microbiologist*" OR "Medical specialist*" OR "Nephrologist*" OR "Neurologist*" OR "Obstetrician*" OR "Oncologist*" OR "Ophthalmologist*" OR "Orthopedic specialist*" OR "Otolaryngologist*" OR "Pathologist*" OR "Paediatrician*" OR "Pediatrician*" OR "Physician*" OR "Neonatologist*" OR "Neurosurgeon*" OR "Pathologist*" OR "Physiatrist*" OR "Psychiatrist*" OR "Physician*" OR "Pulmonologist*" OR "Radiologist*" OR "Radiotherapist*" OR "Rheumatologist*" OR "Surgeon*" OR "Neurosurgeon*" OR "Urologist*" OR "nurse practitioner*" OR "clinician*")	1,227,838
#1	TS=("econsult*" OR "e-consult*" OR "ediagnos*" OR "e diagnos*" OR "mobile health*" OR "mhealth*" OR "m health*" OR "telehealth*" OR "tele health" OR "remote consult*" OR "teleconsult*" OR "tele consult*" OR "telediagnos*" OR "tele diagnos*" OR "telemedic*" OR "tele medic*" OR "telemonitor*" OR "tele monitor*" OR "ehealth*" OR "e-health*" OR "telecare" OR "tele care" OR "digital health" OR "app" OR "apps" OR "smartphone*" OR "phone application*" OR "telephone application*" OR "mobile application*" OR "mobile technolog*" OR "health technolog*" OR "health application*" OR "internet*" OR "world wide web*" OR "webportal*" OR "web portal*" OR "patient portal*" OR "ipad" OR "ipads" OR "telecounsel*" OR "tele counsel*" OR "remote counsel*" OR "distance consult*" OR "distance counsel*" OR "distant consult*" OR "patient monitoring" OR "interactive voice response*" OR "multimedia" OR "Mhapps" OR "e-coach*" OR "wearable*" OR "handheld" OR "virtual patient*" OR "computer" OR "digital*")	1,564,093

For peer review only - http://bmjopen.bmj.com/site/about/guidelines.xhtml

Supplementary Table 3. Quality assessment of included studies (n=33)

**Author (year)**	**Q1** Is the review question clearly and explicitly stated?	**Q2** Were the inclusion criteria appropriate for the review question?	**Q3** Was the search strategy appropriate?	**Q4** Were the sources and resources used to search for studies adequate?	**Q5** Were the criteria for appraising the studies appropriate?[1]	**Q6** Was the critical appraisal conducted by two or more reviewers independently?[1]	**Q7** Were methods implemented to minimise errors in data extraction?	**Q8** Were the methods used to combine studies appropriate?[1,2]	**Q9** Was the likelihood of publication bias assessed?[1]	**Q10** Were recommendations for policy and/or practice supported by the reported data?	**Q11** Were the specific drivers for new research appropriate?
Almathami, Win and Vlahu-Gjorgievska (1)	Yes	Yes	Yes	Yes	Yes	**Unclear**	Yes	Yes	No	Yes	**Unclear**
Antoun (2)	Yes	**Unclear**	**Unclear**	**Unclear**	NA*	NA	**Unclear**	NA	NA	Yes	Yes
Appleton, Williams (3)	Yes	Yes	Yes	Yes	Yes	Yes	Yes	Yes	NA	Yes	Yes
Batterham, Sunderland (4)	Yes	Yes	**Unclear**	Yes	**Unclear**	**Unclear**	**Unclear**	Yes	NA	Yes	Yes
Bokolo (5)	Yes	Yes	Yes	Yes	NA	NA	**Unclear**	Yes	NA	Yes	Yes
Brunton, Bower and Sanders (6)	Yes	Yes	Yes	Yes	Yes	Yes	Yes	Yes	NA	Yes	Yes
Cowan, McKean (7)	Yes	**Unclear**	Yes	No	NA	NA	**Unclear**	Yes	NA	Yes	Yes
de Grood, Raissi (8)	Yes	Yes	Yes	Yes	NA	NA	Yes	Yes	NA	Yes	Yes
De Guzman, Snoswell (9)	Yes	Yes	Yes	Yes	Yes	Yes	Yes	Yes	NA	Yes	Yes
Finkelstein, Knight (10)	Yes	Yes	Yes	Yes	NA	NA	Yes	Yes	NA	Yes	Yes
Gagnon, Ngangue (11)	Yes	Yes	Yes	Yes	NA	NA	Yes	Yes	No	Yes	**Unclear**
Greenhalgh, A'Court and Shaw (12)	Yes	**Unclear**	No	Yes	NA	NA	No	Yes	NA	Yes	Yes
Hassibian and Hassibian (13)	Yes	**Unclear**	No	Yes	NA	NA	**Unclear**	NA	NA	Yes	**Unclear**
Hopstaken, Verweij (14)	Yes	Yes	Yes	Yes	Yes	Yes	Yes	Yes	NA	Yes	Yes
Jacob, Sanchez-Vazquez and Ivory (15)	Yes	Yes	Yes	Yes	Yes	**Unclear**	Yes	Yes	NA	Yes	Yes

For peer review only - http://bmjopen.bmj.com/site/about/guidelines.xhtml

Lewinski, Rushton (16)	Yes	Yes	Yes	Yes	Yes	**Unclear**	Yes	Yes	NA	Yes	Yes
Li, Talaei-Khoei (17)	Yes	Yes	Yes	Yes	**Unclear**	**Unclear**	Yes	Yes	NA	Yes	Yes
Liyanage-Don, Fung (18)	Yes	Yes	Yes	Yes	NA	NA	**Unclear**	Yes	NA	Yes	Yes
Maclure, Stewart and Strath (19)	Yes	Yes	Yes	Yes	Yes	Yes	Yes	Yes	NA	Yes	Yes
Meng, McAiney (20)	Yes	Yes	Yes	Yes	NA	NA	Yes	Yes	NA	Yes	Yes
Metzger, Jatana (21)	Yes	Yes	Yes	Yes	NA	NA	Yes	Yes	NA	Yes	Yes
Mileski, Kruse (22)	Yes	Yes	Yes	Yes	NA	NA	Yes	Yes	NA	Yes	No
Muir, de Boer (23)	Yes	Yes	Yes	Yes	Yes	Yes	No	Yes	NA	Yes	Yes
O'Cathail, Sivanandan (24)	Yes	Yes	Yes	Yes	NA	NA	Yes	Yes	NA	Yes	Yes
Palacholla, Fischer (25)	Yes	Yes	Yes	Yes	NA	NA	Yes	Yes	NA	Yes	Yes
Slater, Campbell (26)	Yes	Yes	Yes	Yes	Yes	Yes	Yes	Yes	NA	Yes	Yes
Smith, Jacobsen (27)	Yes	Yes	Yes	Yes	Yes	Yes	Yes	Yes	NA	Yes	Yes
Varsi, Solberg Nes (28)	Yes	Yes	Yes	Yes	Yes	No	Yes	Yes	NA	Yes	Yes
Vedel, Akhlaghpour (29)	Yes	Yes	Yes	Yes	Yes	Yes	Yes	Yes	**Unclear**	Yes	Yes
Vyas, Hambrick (30)	Yes	Yes	Yes	No	NA	NA	Yes	NA	NA	Yes	Yes
Whitelaw, Pellegrini (31)	Yes	Yes	Yes	Yes	NA	NA	Yes	Yes	NA	Yes	Yes
Xyrichis, Iliopoulou (32)	Yes	Yes	Yes	Yes	Yes	Yes	Yes	Yes	NA	Yes	Yes
Zaman, Khan (33)	Yes	Yes	Yes	Yes	NA	NA	Yes	Yes	NA	Yes	Yes

[1] Questions not applicable for other review types. [2] Question interpreted more broadly in the assessment of other review types: is the synthesis appropriate for the review question? [*] *Not applicable*

For peer review only

References

1. Almathami HKY, Win KT, Vlahu-Gjorgievska E. Barriers and Facilitators That Influence Telemedicine-Based, Real-Time, Online Consultation at Patients' Homes: Systematic Literature Review. J Med Internet Res. 2020;22(2):e16407.
2. Antoun J. Electronic mail communication between physicians and patients: a review of challenges and opportunities. Fam Pract. 2016;33(2):121-6.
3. Appleton R, Williams J, San Juan NV, Needle JJ, Schlief M, Jordan H, et al. Implementation, adoption, and perceptions of telemental health during the COVID-19 pandemic: systematic review. Journal of medical Internet research. 2021;23(12):e31746.
4. Batterham PJ, Sunderland M, Calear AL, Davey CG, Christensen H, Teesson M, et al. Developing a roadmap for the translation of e-mental health services for depression. Aust N Z J Psychiatry. 2015;49(9):776-84.
5. Bokolo AJ. Exploring the adoption of telemedicine and virtual software for care of outpatients during and after COVID-19 pandemic. Ir J Med Sci. 2021;190(1):1-10.
6. Brunton L, Bower P, Sanders C. The Contradictions of Telehealth User Experience in Chronic Obstructive Pulmonary Disease (COPD): A Qualitative Meta-Synthesis. PLoS One. 2015;10(10):e0139561.
7. Cowan KE, McKean AJ, Gentry MT, Hilty DM. Barriers to Use of Telepsychiatry: Clinicians as Gatekeepers. Mayo Clinic Proceedings. 2019;94(12):2510-23.
8. de Grood C, Raissi A, Kwon Y, Santana MJ. Adoption of e-health technology by physicians: a scoping review. J Multidiscip Healthc. 2016;9:335-44.
9. De Guzman KR, Snoswell CL, Taylor ML, Senanayake B, Haydon HM, Batch JA, et al. A Systematic Review of Pediatric Telediabetes Service Models. Diabetes Technol Ther. 2020;22(8):623-38.
10. Finkelstein J, Knight A, Marinopoulos S, Gibbons MC, Berger Z, Aboumatar H, et al. Enabling patient-centered care through health information technology. Evid Rep Technol Assess (Full Rep). 2012(206):1-1531.
11. Gagnon MP, Ngangue P, Payne-Gagnon J, Desmartis M. m-Health adoption by healthcare professionals: a systematic review. Journal of the American Medical Informatics Association. 2016;23(1):212-20.
12. Greenhalgh T, A'Court C, Shaw S. Understanding heart failure; explaining telehealth - a hermeneutic systematic review. BMC Cardiovasc Disord. 2017;17(1):156.
13. Hassibian MR, Hassibian S. Telemedicine acceptance and implementation in developing countries: Benefits, categories, and barriers. Razavi International Journal of Medicine. 2016;4(3).
14. Hopstaken JS, Verweij L, van Laarhoven CJ, Blijlevens NM, Stommel MW, Hermens RP. Effect of digital care platforms on quality of care for oncological patients and barriers and facilitators for their implementation: Systematic review. Journal of Medical Internet Research. 2021;23(9):e28869.
15. Jacob C, Sanchez-Vazquez A, Ivory C. Social, Organizational, and Technological Factors Impacting Clinicians' Adoption of Mobile Health Tools: Systematic Literature Review. Jmir Mhealth and Uhealth. 2020;8(2).
16. Lewinski AA, Rushton S, Van Voorhees E, Boggan JC, Whited JD, Shoup JP, et al. Implementing remote triage in large health systems: A qualitative evidence synthesis. Res Nurs Health. 2021;44(1):138-54.
17. Li J, Talaei-Khoei A, Seale H, Ray P, Macintyre CR. Health Care Provider Adoption of eHealth: Systematic Literature Review. Interact J Med Res. 2013;2(1):e7.
18. Liyanage-Don N, Fung D, Phillips E, Kronish IM. Implementing Home Blood Pressure Monitoring into Clinical Practice. Current Hypertension Reports. 2019;21(2).
19. Maclure K, Stewart D, Strath A. A systematic review of medical and non-medical practitioners' views of the impact of ehealth on shared care. European Journal of Hospital Pharmacy. 2014;21(1):54-62.

For peer review only - http://bmjopen.bmj.com/site/about/guidelines.xhtml

20. Meng G, McAiney C, Perlman CM, McKillop I, Tisseverasinghe T, Chen HH. Service process factors affecting patients' and clinicians' experiences on rapid teleconsultation implementation in out-patient neurology services during COVID-19 pandemic: a scoping review. BMC health services research. 2022;22(1):1-17.
21. Metzger G, Jatana K, Apfeld J, Deans KJ, Minneci PC, Halaweish I. State of telemedicine use in pediatric surgery in the USA-where we stand and what we can gain from the COVID-19 pandemic: A scoping review. World Journal of Pediatric Surgery. 2021;4(1).
22. Mileski M, Kruse CS, Catalani J, Haderer T. Adopting Telemedicine for the Self-Management of Hypertension: Systematic Review. JMIR Med Inform. 2017;5(4):e41.
23. Muir SD, de Boer K, Nedeljkovic M, Meyer D. Barriers and facilitators of videoconferencing psychotherapy implementation in veteran mental health care environments: a systematic review. BMC Health Serv Res. 2020;20(1):999.
24. O'Cathail M, Sivanandan MA, Diver C, Patel P, Christian J. The Use of Patient-Facing Teleconsultations in the National Health Service: Scoping Review. JMIR Med Inform. 2020;8(3):e15380.
25. Palacholla RS, Fischer N, Coleman A, Agboola S, Kirley K, Felsted J, et al. Provider- and Patient-Related Barriers to and Facilitators of Digital Health Technology Adoption for Hypertension Management: Scoping Review. JMIR Cardio. 2019;3(1):e11951.
26. Slater H, Campbell JM, Stinson JN, Burley MM, Briggs AM. End User and Implementer Experiences of mHealth Technologies for Noncommunicable Chronic Disease Management in Young Adults: Systematic Review. J Med Internet Res. 2017;19(12):e406.
27. Smith SM, Jacobsen JHW, Atlas AP, Khoja A, Kovoor JG, Tivey DR, et al. Telehealth in surgery: an umbrella review. ANZ journal of surgery. 2021;91(11):2360-75.
28. Varsi C, Solberg Nes L, Kristjansdottir OB, Kelders SM, Stenberg U, Zangi HA, et al. Implementation Strategies to Enhance the Implementation of eHealth Programs for Patients With Chronic Illnesses: Realist Systematic Review. J Med Internet Res. 2019;21(9):e14255.
29. Vedel I, Akhlaghpour S, Vaghefi I, Bergman H, Lapointe L. Health information technologies in geriatrics and gerontology: a mixed systematic review. Journal of the American Medical Informatics Association. 2013;20(6):1109-19.
30. Vyas KS, Hambrick HR, Shakir A, Morrison SD, Tran DC, Pearson K, et al. A Systematic Review of the Use of Telemedicine in Plastic and Reconstructive Surgery and Dermatology. Ann Plast Surg. 2017;78(6):736-68.
31. Whitelaw S, Pellegrini DM, Mamas MA, Cowie M, Van Spall HGC. Barriers and facilitators of the uptake of digital health technology in cardiovascular care: a systematic scoping review. Eur Heart J Digit Health. 2021;2(1):62-74.
32. Xyrichis A, Iliopoulou K, Mackintosh NJ, Bench S, Terblanche M, Philippou J, Sandall J. Healthcare stakeholders' perceptions and experiences of factors affecting the implementation of critical care telemedicine (CCT): qualitative evidence synthesis. Cochrane Database Syst Rev. 2021;2(2):Cd012876.
33. Zaman SB, Khan RK, Evans RG, Thrift AG, Maddison R, Islam SMS. Exploring Barriers to and Enablers of the Adoption of Information and Communication Technology for the Care of Older Adults With Chronic Diseases: Scoping Review. JMIR aging. 2022;5(1):e25251.

For peer review only - http://bmjopen.bmj.com/site/about/guidelines.xhtml

Reporting checklist for systematic review (with or without a meta-analysis).

Based on the PRISMA guidelines.

Instructions to authors

Complete this checklist by entering the page numbers from your manuscript where readers will find each of the items listed below.

Your article may not currently address all the items on the checklist. Please modify your text to include the missing information. If you are certain that an item does not apply, please write "n/a" and provide a short explanation.

Upload your completed checklist as an extra file when you submit to a journal.

In your methods section, say that you used the PRISMAreporting guidelines, and cite them as:

Page MJ, McKenzie JE, Bossuyt PM, Boutron I, Hoffmann TC, Mulrow CD, Shamseer L, Tetzlaff JM, Akl EA, Brennan SE, Chou R, Glanville J, Grimshaw JM, Hróbjartsson A, Lalu MM, Li T, Loder EW, Mayo-Wilson E, McDonald S, McGuinness LA, Stewart LA, Thomas J, Tricco AC, Welch VA, Whiting P, Moher D. The PRISMA 2020 statement: An updated guideline for reporting systematic reviews

		Reporting Item	Page Number
**Title**			
Title	#1	Identify the report as a systematic review	1
**Abstract**			

For peer review only

Abstract	#2	Report an abstract addressing each item in the PRISMA 2020 for Abstracts checklist	2
**Introduction**			
Background/rationale	#3	Describe the rationale for the review in the context of existing knowledge	5
Objectives	#4	Provide an explicit statement of the objective(s) or question(s) the review addresses	5
**Methods**			
Eligibility criteria	#5	Specify the inclusion and exclusion criteria for the review and how studies were grouped for the syntheses	7
Information sources	#6	Specify all databases, registers, websites, organisations, reference lists, and other sources searched or consulted to identify studies. Specify the date when each source was last searched or consulted	8
Search strategy	#7	Present the full search strategies for all databases, registers, and websites, including any filters and limits used	8
Selection process	#8	Specify the methods used to decide whether a study met the inclusion criteria of the review, including how many reviewers screened each record and each report retrieved, whether they worked independently, and, if applicable, details of automation tools used in the process	9

For peer review only

Data collection process	#9	Specify the methods used to collect data from reports, including how many reviewers collected data from each report, whether they worked independently, any processes for obtaining or confirming data from study investigators, and, if applicable, details of automation tools used in the process	9
Data items	#10a	List and define all outcomes for which data were sought. Specify whether all results that were compatible with each outcome domain in each study were sought (for example, for all measures, time points, analyses), and, if not, the methods used to decide which results to collect	9
Study risk of bias assessment	#11	Specify the methods used to assess risk of bias in the included studies, including details of the tool(s) used, how many reviewers assessed each study and whether they worked independently, and, if applicable, details of automation tools used in the process	10
Effect measures	#12	Specify for each outcome the effect measure(s) (such as risk ratio, mean difference) used in the synthesis or presentation of results	NA
Synthesis methods	#13a	Describe the processes used to decide which studies were eligible for each synthesis (such as tabulating the study intervention characteristics and comparing against the planned groups for each synthesis (item #5))	9-10

For peer review only

Synthesis methods	#13b	Describe any methods required to prepare the data for presentation or synthesis, such as handling of missing summary statistics or data conversions	9-10
Synthesis methods	#13c	Describe any methods used to tabulate or visually display results of individual studies and syntheses	9-10
Synthesis methods	#13d	Describe any methods used to synthesise results and provide a rationale for the choice(s). If meta-analysis was performed, describe the model(s), method(s) to identify the presence and extent of statistical heterogeneity, and software package(s) used	9-10
Synthesis methods	#13e	Describe any methods used to explore possible causes of heterogeneity among study results (such as subgroup analysis, meta-regression)	NA
Synthesis methods	#13f	Describe any sensitivity analyses conducted to assess robustness of the synthesised results	NA
Reporting bias assessment	#14	Describe any methods used to assess risk of bias due to missing results in a synthesis (arising from reporting biases)	NA
Certainty assessment	#15	Describe any methods used to assess certainty (or confidence) in the body of evidence for an outcome	NA
Data items	#10b	List and define all other variables for which data were sought (such as participant and intervention characteristics, funding sources). Describe any	9

For peer review only

		assumptions made about any missing or unclear information	
**Results**			
Study selection	#16a	Describe the results of the search and selection process, from the number of records identified in the search to the number of studies included in the review, ideally using a flow diagram (http://www.prisma-statement.org/PRISMAStatement/FlowDiagram)	11
Study selection	#16b	Cite studies that might appear to meet the inclusion criteria, but which were excluded, and explain why they were excluded	11
Study characteristics	#17	Cite each included study and present its characteristics	14
Risk of bias in studies	#18	Present assessments of risk of bias for each included study	NA
Results of individual studies	#19	For all outcomes, present for each study (a) summary statistics for each group (where appropriate) and (b) an effect estimate and its precision (such as confidence/credible interval), ideally using structured tables or plots	NA
Results of syntheses	#20a	For each synthesis, briefly summarise the characteristics and risk of bias among contributing studies	NA
Results of syntheses	#20b	Present results of all statistical syntheses conducted. If meta-analysis was done, present for each the summary	NA

For peer review only

		estimate and its precision (such as confidence/credible interval) and measures of statistical heterogeneity. If comparing groups, describe the direction of the effect	
Results of syntheses	#20c	Present results of all investigations of possible causes of heterogeneity among study results	NA
Results of syntheses	#20d	Present results of all sensitivity analyses conducted to assess the robustness of the synthesised results	NA
Risk of reporting biases in syntheses	#21	Present assessments of risk of bias due to missing results (arising from reporting biases) for each synthesis assessed	NA
Certainty of evidence	#22	Present assessments of certainty (or confidence) in the body of evidence for each outcome assessed	NA
**Discussion**			
Results in context	#23a	Provide a general interpretation of the results in the context of other evidence	20
Limitations of included studies	#23b	Discuss any limitations of the evidence included in the review	22
Limitations of the review methods	#23c	Discuss any limitations of the review processes used	22
Implications	#23d	Discuss implications of the results for practice, policy, and future research	23
**Other information**			

For peer review only

Registration and protocol	#24a	Provide registration information for the review, including register name and registration number, or state that the review was not registered	NA
Registration and protocol	#24b	Indicate where the review protocol can be accessed, or state that a protocol was not prepared	n/a
Registration and protocol	#24c	Describe and explain any amendments to information provided at registration or in the protocol	NA
Support	#25	Describe sources of financial or non-financial support for the review, and the role of the funders or sponsors in the review	24
Competing interests	#26	Declare any competing interests of review authors	24
Availability of data, code, and other materials	#27	Report which of the following are publicly available and where they can be found: template data collection forms; data extracted from included studies; data used for all analyses; analytic code; any other materials used in the review	24

The PRISMA checklist is distributed under the terms of the Creative Commons Attribution License CC-BY. This checklist was completed on 19. May 2023 using https://www.goodreports.org/, a tool made by the EQUATOR Network in collaboration with Penelope.ai

For peer review only

BMJ Open

Implementation barriers and facilitators of remote monitoring, remote consultation and digital care platforms through the eyes of healthcare professionals: a review of reviews

Journal:	*BMJ Open*
Manuscript ID	bmjopen-2023-075833.R2
Article Type:	Original research
Date Submitted by the Author:	30-Apr-2024

Complete List of Authors:	Oudbier, Susan; Amsterdam UMC Location VUmc, Outpatient Division, Amsterdam Public Health research institute, Location Vrije Universiteit Amsterdam, De Boelelaan 1117; Amsterdam UMC Location AMC, Department of Medical Psychology, Amsterdam Public Health research institute, Meibergdreef 9<br>Souget-Ruff, S.P.; Amsterdam UMC Location AMC, Department of Medical Psychology, Amsterdam Public Health research institute, Meibergdreef 9<br>Chen, B.S.J.; Amsterdam UMC Location AMC, Department of Medical Psychology, Amsterdam Public Health research institute, Meibergdreef 9<br>Ziesemer, K.A.; Vrije Universiteit Amsterdam, Medical Library<br>Meij, J.J.; Amsterdam UMC Location VUmc, Outpatient Division, Amsterdam Public Health research institute, Location Vrije Universiteit Amsterdam, De Boelelaan 1117; National University of Singapore Yong Loo Lin School of Medicine<br>Smets, Ellen M. A.; Amsterdam UMC Location AMC, Department of Medical Psychology, Amsterdam Public Health research institute, Meibergdreef 9

**Primary Subject Heading**:	Health informatics
Secondary Subject Heading:	Communication, Health informatics, Medical management, Public health
Keywords:	Health informatics < BIOTECHNOLOGY & BIOINFORMATICS, Information technology < BIOTECHNOLOGY & BIOINFORMATICS, Telemedicine < BIOTECHNOLOGY & BIOINFORMATICS

SCHOLARONE™
Manuscripts

I, the Submitting Author has the right to grant and does grant on behalf of all authors of the Work (as defined in the below author licence), an exclusive licence and/or a non-exclusive licence for contributions from authors who are: i) UK Crown employees; ii) where BMJ has agreed a CC-BY licence shall apply, and/or iii) in accordance with the terms applicable for US Federal Government officers or employees acting as part of their official duties; on a worldwide, perpetual, irrevocable, royalty-free basis to BMJ Publishing Group Ltd ("BMJ") its licensees and where the relevant Journal is co-owned by BMJ to the co-owners of the Journal, to publish the Work in this journal and any other BMJ products and to exploit all rights, as set out in our licence.

The Submitting Author accepts and understands that any supply made under these terms is made by BMJ to the Submitting Author unless you are acting as an employee on behalf of your employer or a postgraduate student of an affiliated institution which is paying any applicable article publishing charge ("APC") for Open Access articles. Where the Submitting Author wishes to make the Work available on an Open Access basis (and intends to pay the relevant APC), the terms of reuse of such Open Access shall be governed by a Creative Commons licence – details of these licences and which Creative Commons licence will apply to this Work are set out in our licence referred to above.

Other than as permitted in any relevant BMJ Author's Self Archiving Policies, I confirm this Work has not been accepted for publication elsewhere, is not being considered for publication elsewhere and does not duplicate material already published. I confirm all authors consent to publication of this Work and authorise the granting of this licence.

For peer review only

Implementation barriers and facilitators of remote monitoring, remote consultation and digital care platforms through the eyes of healthcare professionals: a review of reviews

Oudbier, S.J.[a,b], MD, Souget-Ruff, S.P.[b], Chen, B.S.J.[b], Bsc, Ziesemer, K.A[c], Msc., Meij, J.J., PhD[a,d], Smets, E.M.A., PhD[b]

[a] Amsterdam UMC location Vrije Universiteit Amsterdam, Outpatient Division, Amsterdam Public Health research institute, De Boelelaan 1117, Amsterdam, The Netherlands

[b] Amsterdam UMC location AMC Amsterdam, Department of Medical Psychology, Amsterdam Public Health research institute, Meibergdreef 9, Amsterdam, The Netherlands

[c] Medical Library, Vrije Universiteit, Amsterdam, The Netherlands

[d] Yong Loo Lin School of Medicine, National University of Singapore, Singapore.

Corresponding author

Susan Oudbier

Outpatient Division (VIII)

Location AMC | Tks0-136 Meibergdreef 9, 1105 AZ Amsterdam

T: 0613455752 | E: s.oudbier@amsterdamumc.nl

For peer review only - http://bmjopen.bmj.com/site/about/guidelines.xhtml

Abstract

Objectives

Digital transformation in healthcare is a necessity considering the steady increase in healthcare costs, the growing ageing population, and rising number of people living with chronic diseases. The implementation of digital health technologies in patient care is a potential solution to these issues, however, some challenges remain. In order to navigate such complexities, the perceptions of healthcare professionals (HCPs) must be considered. The objective of this umbrella review is to identify key barriers and facilitators involved in digital health technology implementation, from the perspective of HCPs.

Design

Systematic umbrella review following the Preferred Reporting Items for Systematic Reviews and Meta-Analyses (PRISMA) statement.

Data sources

Embase.com, PubMed, and Web of Science Core Collection were searched for existing reviews dated up to June 17, 2022. Search terms included digital health technology, combined with terms related to implementation, and variations in terms encompassing HCP, such as physician, doctor, and the medical discipline.

Eligibility criteria

Quantitative and qualitative reviews evaluating digital technologies that included patient interaction were considered eligible. Three reviewers independently synthesised and assessed eligible reviews and conducted a critical appraisal.

Data extraction and synthesis

For peer review only - http://bmjopen.bmj.com/site/about/guidelines.xhtml

Regarding the data collection, two reviewers independently synthesized and interpreted data on barriers and facilitators.

Results

Thirty-three reviews met the inclusion criteria. Barriers and facilitators were categorised into four levels; 1) the organisation, 2) the HCP, 3) the patient, and 4) technical aspects. The main barriers and facilitators identified were (lack of) training (n=22/33), (un)familiarity with technology (n=17/33), (loss of) communication (n=13/33), and security and confidentiality issues (n=17/33). Barriers of key importance included increased workload (n=16/33), the technology undermining aspects of professional identity (n=11/33), HCP uncertainty about patients' aptitude with the technology (n=9/33), and technical issues (n=12/33).

Conclusions

The implementation strategy should address the key barriers highlighted by HCPs, for instance, by providing adequate training to familiarise HCPs with the technology, adapting the technology to the patient preferences, and addressing technical issues. Barriers on both HCP-and patient-levels can be overcome by investigating the needs of the end-users. As we shift from traditional face-to-face care models towards new modes of care delivery, further research is needed to better understand the role of digital technology in the HCP-patient relationship.

Keywords digital transformation, health care professionals, barriers, facilitators, patient interaction, digital technology, implementation

For peer review only - http://bmjopen.bmj.com/site/about/guidelines.xhtml

Strengths and limitations of this study

- We conducted a review of reviews (umbrella review) on digital health implementation where we followed the Preferred Reporting Items for Systematic Review and Meta-Analysis-guidelines.
- This review encompasses digital health technologies which include patient interaction.
- The screening and quality assessment process of this systematic review were conducted in duplicate.
- A meta-analysis was not conducted since pooling of data was impossible due to the heterogeneity among studies investigating digital technologies and outcomes.

For peer review only

Word count: 4200

For peer review only - http://bmjopen.bmj.com/site/about/guidelines.xhtml

Introduction

Globally, healthcare faces huge challenges due to an increase in individuals living with chronic diseases, an aging population, and increasing healthcare expenditures (1). Therefore, a so called digital transformation is advocated considering the opportunities the implementation of technology has in terms of reducing costs (2), improving quality of care (3), and reducing the burden on healthcare professionals (HCPs) (4, 5). The introduction of the Electronic Health Record (EHR) was a major digital transformer. EHR improved overall quality of care by increasing healthcare accessibility, communication, and the exchange of data between patients and HCPs (6-8). The introduction of digital health, defined by the *World Health Organisation* as 'the use of information and communications technology in support of health and health-related fields', promises to be another innovation in healthcare (9). Telemedicine, smartphone apps, and wearables (i.e., small electronic devices that use sensors to measure biometric values and physical activity) (10) are examples of digital health technologies that have been developed to address shortcomings in the healthcare sector. Digital health is expected to lead to improved cost-efficiency (11), enhanced patient diagnosis (12), and improved adherence to treatment and patient outcomes (13).

The importance of digital health is growing for several reasons. Firstly, efficiency must be improved to address increasing healthcare costs, as well as demographic and epidemiological challenges such as increased life expectancy, increased rates of chronic diseases, and population growth (14). Secondly, there is huge societal demand for digital transformation in healthcare, as many processes in other sectors are now digitalized. For example, passengers in the airline industry use digital technology at every phase of their journey from booking a plane ticket, to choosing a seat and checking in for flights (15). In contrast, digitalization is lagging behind in the European healthcare system. This is due to difficulties in interoperating between systems, data security and privacy concerns, and the uptake of technologies taking longer than anticipated (16, 17). Inadequate IT infrastructure, technical limitations, and lack of a central authority for standardisation mean that the

For peer review only - http://bmjopen.bmj.com/site/about/guidelines.xhtml

Netherlands face more challenges with the exchange of data between healthcare sectors, and international data exchanges, than other European countries (18). A final reason for the need of digital transformation in healthcare is related to the significant data storage requirements needed to accelerate and increase the efficiency of health services via big data analytics (7, 19). In order to manage these data streams efficiently and effectively, digital transformation is a necessity.

Despite all these positive implications for healthcare, the implementation, adoption, and scaling of digital health remains a challenge (20). Many digital health technologies require some degree of patient interaction, such as the ability to communicate with the HCP within the EHR (i.e., to have a video-consultation or to participate in disease monitoring). Digital transformation therefore does not emerge from the enabling of technology alone; all stakeholders are required to successfully implement such technology within the healthcare system (14). This implies that in order to guarantee adoption, it is crucial to be attentive to the usability by including the end-users of the technology (e.g., the patients and HCPs), in the implementation process (21). Even though HCPs aim to deliver high-quality care, their resistance to change is a frequently mentioned barrier to the implementation of new technologies (22).

Thus, understanding the barriers and facilitators as perceived by HCPs is important to enable smooth introduction and efficient use of digital health technologies (22). Many studies and reviews have been executed on the subject of implementation in healthcare; however, research on factors that influence implementation of technology often focuses on a single technology (23, 24), or a specific disease (25, 26). Drawing from these reviews, the perspective of one of the key stakeholders within this implementation, that is the HCP, is often not the main focus of the study. To date, no study has systematically inventoried barriers and facilitators of implementation of digital health technology concentrating on the perspective of the HCP. Furthermore, no review investigated which barriers and facilitators are generic within technology implementation. That is, which barriers and facilitators apply regardless of the technology involved, and which vary per digital technology. Thus, the aim of

For peer review only - http://bmjopen.bmj.com/site/about/guidelines.xhtml

this review is to systematically review the barriers and facilitators of implementations of digital health technologies that include patient interaction, specifically focusing on the HCP's perspective.

For peer review only - http://bmjopen.bmj.com/site/about/guidelines.xhtml

Methods

This review followed the guidelines of the Preferred Reporting Items for Systematic Reviews and Meta-Analyses (PRISMA) statement (27).

Eligibility criteria

For this review, we considered all eligible literature reviews (regardless of design) and meta-analyses studying the implementation of digital health technologies applying patient interaction and barriers and/or facilitators related to HCPs. Inclusion criteria were 1) the publication type being: literature review and/or meta-analysis, 2) describing barriers, facilitators, successes, inhibitions, and/or failures for the implementation, adoption and maintenance of the digital health technology 3) the technology described should encompass patient interaction, and 4) the barriers and/or facilitators have been described from the perspective of the HCP (i.e. medical specialists, nurse practitioners, and physician assistants) (Supplementary Table 1). A barrier is defined as any factor that hinders implementation of the technology. A facilitator is defined as something that enables the implementation of the technology. Implementation refers to the process of putting to use or integrating the digital health technology into the clinical workflow (28), including the actual adoption and maintenance of the technology. The adoption of a technology is defined as the commitment or uptake of the technology by the HCPs (29), whereas the maintenance of a technology is defined as the ability to consistently achieve the benefits of the digital technology after implementation (28). We operationalised the perspective of the HCPs by performing data extraction in three rounds. Firstly, we decided whether the HCP's perspective was the main focus of the included review by considering the title and research question. In that case, all barriers and facilitators were included in this review. Secondly, if the HCP was not a main focus of the research, we examined whether the perspective of the HCP was mentioned as a separate stakeholder in the review. If so, we included the barriers and facilitators as reported to represent their perspective. Thirdly, if healthcare professionals were part of a broader group of stakeholders, including e.g. patients or managers, we

For peer review only - http://bmjopen.bmj.com/site/about/guidelines.xhtml

discussed whether the perspective of the HCP was sufficiently exposed. In case of consensus, we included the barriers and facilitators mentioned by that review. Since there is a clear distinction between care provided outside the hospital (i.e., by the general practitioner, and care provided in the hospital, studies investigating the implementation/adoption of digital technologies in primary care have not been included). Exclusion criteria consisted of 1) article written in language other than Dutch, German, or English, 2) publication type such as research protocol, opinion paper, original empirical study, conference abstract, 3) no digital health technology described, 4) focus on phase other than adoption/implementation/maintenance phase of the technology, 5) no patient interaction with the technology, 6) different target user (managerial/primary care/nurse/patient), 7) article describes a framework related to technology implementation, and 8) no barriers/facilitators described.

Information sources and search

The PICO search strategy was executed to systematically review the literature (30). In collaboration with a medical information specialist (KAZ), reviews were identified from several bibliographic databases including PubMed, Embase.com and Clarivate Analytics/Web of Science Core Collection. The search was conducted on June 29, 2021 and updated on June 17, 2022. Search terms including synonyms, closely related words and keywords were used as index terms or free-text words: "digital*", "transform*" and "physicians". We used a methodological search filter to specify the results to reviews, meta-analyses and other summarising evidence. Duplicate articles were excluded using the R-package "ASYSD" an automated deduplication tool (31) followed by manual deduplication in Endnote (X20.0.3) by the medical information specialist (KAZ). The full search strategy used for each database is detailed in the supplementary material (Supplementary Table 2).

Study selection

Using the online Rayyan tool for systematic reviews, three authors independently reviewed the articles on title and abstract using the aforementioned criteria (32). The first author (SJO) reviewed

For peer review only - http://bmjopen.bmj.com/site/about/guidelines.xhtml

all articles, and the second and the third author (BSJC, SPR) both screened 50% of the articles. Disagreements in the assessment by the reviewers were discussed until consensus was reached. A third reviewer (EMAS/JJM) was consulted when consensus could not be reached. The updated search (on June 17, 2022) was carried out by two reviewers (SJO, SPR), both authors screened 50% of the articles on the updated search. Articles about which there was doubt were discussed until consensus was reached. In total, four articles could initially not be retrieved, one of which was eventually obtained via a librarian.

Data collection and synthesis

After duplicate removal, data from the selected reviews were independently extracted by two authors (SJO, BSJC, SPR). Disagreements regarding the extracted barriers and facilitators were addressed in a consensus meeting. The following information was extracted: 1) first author, 2) publication date, 3) type of review, 4) digital health technologies reviewed, and 5) facilitators and barriers related to implementation. A meta-analysis was not performed since pooling of data was impossible due to heterogeneity of the studies investigating digital technologies and outcomes. After extraction of the barriers and facilitators per review, three authors (SJO, BSJC, SPR) had a consensus meeting to classify the barriers and facilitators. Since there was overlap between the type of digital health technologies included in this review and our categorisation of the type of technologies, barriers and facilitators could be allocated to multiple categories.

We distinguished three types of digital health technologies, for which we used definitions derived from the literature. Firstly, remote consultation (RC) is referred to as a remote consultation between a HCP and a patient by video or phone (33). Remote monitoring (RM) is defined as “an ambulatory, non-invasive digital technology used to capture patient data in real time and transmit health information for assessment by a health professional or for self-management.” (34,p4). Lastly, we considered a digital care platform (DCP) as a facilitator of digital exchange between a patient and HCP, enabling interaction, coordination, or transaction (35). To ensure the best classification

For peer review only - http://bmjopen.bmj.com/site/about/guidelines.xhtml

possible, we assigned the digital technologies described in the reviews in duplicate (SJO, SPR) to the most fitting category, and in case of overlap, into multiple categories. Finally, one reviewer (SJO) independently categorised the extracted barriers and facilitators. A second reviewer (SPR) repeated this process of categorisation. Disagreements on barriers and facilitators were discussed until consensus was reached.

Quality assessment

The quality of the studies was independently assessed by three reviewers (SJO, BSJC, SPR). BSJC and SPR assessed 50% of the studies, using the Joanna Briggs Institute (JBI) approach for the quality for Systematic Reviews and Research Syntheses (36). A low-quality article is defined when less than five questions within the assessment have been answered with 'Yes'. There were no exclusions made on the basis of a minimum quality threshold. Disagreements in the assessment were resolved until consensus was reached. Some questions (Q5, Q6, and Q9) from this tool did not apply to reviews other than systematic reviews (e.g. scoping reviews); in these cases we reported these questions as not applicable. We interpreted question number 8 (Q8) broadly, as to whether or not synthesis of data was in line with the research question.

Patient and Public Involvement

No patient involved.

For peer review only

For peer review only - http://bmjopen.bmj.com/site/about/guidelines.xhtml

Results

The literature search generated a total of 10614 references: 4945 in PubMed, 2976 in Embase.com and 2693 in Web of Science Core Collection. After removing duplicates of references that were selected from more than one database, 6719 references remained. The search and screening process is illustrated in the PRISMA flow diagram in Figure 1.

Review characteristics

All reviews were published between 2012 and 2021, with more than half of the reviews (n=19) published in the last five years. Most reviews were either systematic (n=17) or scoping reviews (n=8). The majority of digital technologies examined in all reviews encompassed telehealth or remote monitoring of patients (Table 1). Most of the diseases examined were cardiovascular disease (n=9), mental health (n=7), diabetes mellitus (DM) (n=5), and chronic obstructive pulmonary disease (n=4). Three of the included reviews reported only barriers without facilitators. The full search quality assessment is detailed in the supplementary material (Supplementary Table 3) Two articles (Antoun (37) and Hassibian and Hassibian (38)) scored low on the quality assessment. The main reason for reduction in quality scores was that a number of questions (Q5, Q6, Q9) did not apply because they concerned another type of review than a systematic review.

Barriers and facilitators reported by the included reviews

Generic barriers and facilitators

Barriers and facilitators from the perspective of HCPs, as identified in the included studies, have been organised into the following levels: barriers and facilitators related to the organisation, the individual HCP, the patient, and the technology. Table 2 lists the generic barriers and facilitators for all digital technology types. Overall, the included reviews report less facilitators than barriers. Major overarching themes that some reviews reported as HCP related barriers and others as facilitators

For peer review only - http://bmjopen.bmj.com/site/about/guidelines.xhtml

For peer review only

include (lack of) training (n= 22/33), (un)familiarity with technology (n=17/33), (loss of) communication (n=13/33), and security and confidentiality issues (n=17/33).

Generic barriers on the organisational level that were reported to be experienced by HCPs include an increased workload (n=16/33), medico legal concerns (n=8/33), and lack of desire to change clinical paradigms (n=5/33). On the HCP level, barriers that have been reported encompass the technology possibly undermining aspects of professional identity (n=11/33), impeding the patient-clinician relationship (n=9/33), and undermining holistic surveillance (n=8/33). On the patient level, major barriers as reported by HCPs were questioning the ability of patients to use the technology (n=9/33), perceiving discomfort with engaging patients to use and to maintain to use the technology (n=4/33), and low health- or general literacy of patients (n=4/33). Lastly, generic barriers reported on the technological level include technical issues such as access to Wi-Fi or unreliability of the system (n=12/33), lack of harmonisation of systems (n=10/33), and negative perception of the technologies aesthetics or design (n=3/33).

Generic facilitators on the organisational level as reported by HCPs included increased efficiency (n=13/33), scheduling capability and flexibility (n=7/33), use of champions (n=7/33), and new roles and responsibilities when using the digital technology (n=5/33). On the HCP level, the barriers and facilitators reported could act both ways, for instance, (lack of) motivation and engagement (n=11/33), level of satisfaction of the HCP (n=6/33), and that the technology could help influencing the decision making process (n=5/33). On the patient level, patient empowerment (n=3/33) was reported by HCPs as a facilitator for implementation. Lastly, technological barriers as reported by HCPs were also reported as facilitators, and include (lack of) usefulness and HCPs perceiving no added value of the technology (n=12/33), and (lack of) usability (n=11/33).

Barriers and facilitators divided per type of technology

For peer review only

Table 3 lists the barriers and facilitators divided by type of technology: remote consultation (n=27/33), remote monitoring (n=23/33), and digital care platforms (n=16/33). Each type of technology is discussed below.

Remote consultation

HCP related barriers regarding RC include lack of feedback or evaluation of the technology, pressure from other departments or hospitals, HCPs perceiving a sense of isolation when using the technology, and concerns of patients satisfaction and dependency. Facilitators regarding RC include good communication in network with other stakeholders.

Remote monitoring

With regard to RM, barriers reported by HCPs involve loss of productivity during the implementation of the digital health technology, lack of feedback or evaluation of the digital technology, a culture that is resistant to change, difficulties establishing a trusting relationship with a patient because of distance, and dependency of patients on monitoring. Facilitators specific to RM include proof of utility of the RM through research.

Digital care platforms

Barriers reported by HCP for DCP include concerns about loss of productivity during implementation, and reduced patient satisfaction. Facilitators reported that were specific to DCP include a perceived competitive advantage due to the implementation of this technology by creating new opportunities, and proven utility of the platform, preferably through research.

For peer review only - http://bmjopen.bmj.com/site/about/guidelines.xhtml

Table 1. Review characteristics (n=33)

**Author (year)**	**Type of technology[c]**	**Description from review article**	**Disease/Discipline**
Almathami, Win and Vlahu-Gjorgievska (39)	RC	Home online health consultation systems	All health conditions (mainly cardiovascular, COPD, psychotherapy, DM)
Antoun (37)[a]	RC	Electronic mail communication	Not specified
Appleton, Williams (40)	RC, DCP	Telemental health	Mental health problems
Batterham, Sunderland (41)	RC, DCP	E-mental health services	Depression
Bokolo (42)[a]	RC, RM, DCP	Telemedicine and virtual software	For early diagnosis and follow-up
Brunton, Bower and Sanders (43)	RC, RM	Telehealth	COPD
Cowan, McKean (44)[a]	RC	Tele psychiatry (2-way video therapy)	Psychiatry
de Grood, Raissi (45)[b]	RM, DCP	Ehealth technology (EHR, EMR, telemedicine, health information technology, mHealth, e-Prescribing)	Not specified
De Guzman, Snoswell (46)	RC, RM	Telediabetes service	Paediatrics
Finkelstein, Knight (47)[a]	RC, RM, DCP	Health IT applications (telemedicine, telemonitoring, clinical decision aids, IT-guided disease management)	DM, heart disease, cancer, and other
Gagnon, Ngangue (48)	RC, RM, DCP	mHealth applications (smartphone, personal digital assistant, remote monitoring system, etc.)	Not specified
Greenhalgh, A'Court and Shaw (49)[a]	RC, RM	Telehealth (remote monitoring of symptoms, behaviour or events, biological data captured such as ECG, heart rate, blood pressure, and captured data via implantable devices)	Heart failure management
Hassibian and Hassibian (38)[a]	RC, RM	Telemedicine (advice and consultation)	Chronic diseases (such as DM, hypertension, heart diseases)
Hopstaken, Verweij (50)	DCP	Digital care platforms (general information, patient-specific information, communication possibilities with their HCP via chat, e-Consult or e-mail)*	Oncology
Jacob, Sanchez-Vazquez and Ivory (51)	RM	Mobile health	Mainly acute care, COPD or congestive heart failure, cardiovascular disease, DM
Lewinski, Rushton (52)	RC, RM	Telehealth to perform remote triage and monitoring	Adults and their families and caregivers
Li, Talaei-Khoei (53)	RC, RM, DCP	EHealth applications (EHR/EMR, Telemedicine/Telehealth, Intensive Care Information System, e-discharge, Anaesthesia Information Management System, electronic logistics information system)	Not specified
Liyanage-Don, Fung (54)[b]	RM	Home monitoring	Hypertension management
Maclure, Stewart and Strath (55)	RC, RM, DCP	Ehealth on shared care (telemedicine, generalised Ehealth implementation, EHR)	Not specified
Meng, McAiney (56)[b]	RC	Teleconsultation	Neurology
Metzger, Jatana (57)[b]	RC	Telemedicine	Paediatric surgery
Mileski, Kruse (58)	RC, RM, DCP	Telemedicine	Hypertension
Muir, de Boer (59)	RC	Online videoconferencing (psycho)therapy	Veteran mental healthcare
O'Cathail, Sivanandan (60)[b]	RC	Patient-facing teleconsultations	Accident/emergency, psychiatry/psychology, neurology
Palacholla, Fischer (61)[b]	RC, RM, DCP	Digital health technology	Hypertension
Slater, Campbell (62)	RM	Mobile health technologies	Non-communicable chronic diseases in young adults
Smith, Jacobsen (63)	RC, RM, DCP	Telehealth	Surgery
Varsi, Solberg Nes (64)	RC, RM, DCP	Ehealth programmes (mHealth, telehealth, and patient portals)	Chronic conditions (arthritis, chronic pain, COPD, obesity, DM, and mental disorder)
Vedel, Akhlaghpour (65)	RC, RM, DCP	Health information-/Telecare technologies (telecare, EHR, decision support systems, health information on the web)	Geriatrics, gerontology
Vyas, Hambrick (66)[a]	RC, RM, DCP	Telemedicine	Plastic and reconstructive surgery, dermatology
Whitelaw, Pellegrini (67)[b]	RC, RM	Remote monitoring, tele-visits/virtual visits	Cardiovascular care
Xyrichis, Iliopoulou (68)	RM	Telemedicine	Intensive care
Zaman, Khan (69)[b]	RC, RM, DCP	Information and communication technology	Older adults with chronic diseases

COPD: Chronic Obstructive Pulmonary Disease, DCP: Digital Care Platform, DM: Diabetes Mellitus, ECG: Electrocardiogram, EHR: Electronic Health Record, EMR: Electronic Medical Record, RC: Remote Consultation, RM: Remote Monitoring, RT: Remote Triage [a] Other reviews than systematic, [b] Scoping reviews, [c] Three types of technologies were distinguished: digital care platforms (DCP), remote consultation (RC), and remote monitoring (RM)

For peer review only - http://bmjopen.bmj.com/site/about/guidelines.xhtml

Table 2. Generic barriers and facilitators in digital technology implementation, from the perspective of the HCP (n=33)

Level of barrier or facilitator	Barrier (-) Facilitator (+)	# of reviews	% of Reviews	References
**Organisational**				
(Lack of) training	-/+	22/33		(39-42, 44-49, 51-56, 58, 59, 61, 64, 67, 68)
(Lack of) time and resources	-/+	17/33		(40, 45, 46, 48-50, 52-55, 58, 59, 62-64, 68, 69)
Increase in workload	-	16/33		(37, 40, 43, 45, 47-49, 51, 52, 54, 55, 58, 61, 62, 67, 68)
(Lack of) leadership and support	-/+	15/33		(40, 44-49, 51-53, 55, 56, 61, 67, 68)
(Lack of) reimbursement, financial incentives	-/+	13/33		(37, 41, 44, 45, 47, 48, 51, 53, 54, 58, 61, 62, 67)
(Lack of) integration in clinical workflow	-/+	13/33		(42, 44, 46-49, 51, 53, 54, 60, 61, 64, 68)
Increased efficiency	+	13/33		(39, 40, 44, 47, 48, 50, 51, 55, 57, 58, 60, 63, 67)
Impact on (health) outcomes	-/+	12/33		(38, 44, 48, 51, 52, 54, 55, 57, 58, 60, 61, 68)
Medico legal concerns	-	8/33		(37, 44, 45, 48, 51, 53, 55, 58)
Scheduling capability and flexibility	+	7/33		(39, 40, 44, 48, 51, 52, 60)
(Absence of) champions	+	7/33		(44-46, 49, 53, 59, 61)
Working relationships	-/+	7/33		(48, 49, 51, 52, 55, 58, 68)
Convenience (cost-efficient, travel-saving, time-saving)	-/+	6/33		(39, 49, 51, 56, 58, 63)
New roles and responsibilities	+	5/33		(51-53, 64, 68)
(Lack of) desire to change clinical paradigms	-	5/33		(41, 44, 45, 49, 69)
(Lack of) standardizations	-	4/33		(41, 47, 51, 69)
Socio-political tensions	-	3/33		(48, 52, 55)
Lack of purpose and implementation plan	-	2/33		(48, 68)
**HCP**				
(Un)familiarity with technology	-/+	17/33		(38, 41-43, 47-49, 51-54, 58-60, 65, 68, 69)
(Loss of) communication	-/+	13/33		(37, 39, 40, 48, 51, 52, 56-58, 62, 66-68)
Undermines aspects of professional identity	-	11/33		(40, 43, 45, 47-49, 51-53, 55, 58)
(Lack of) motivation and engagement	-/+	11/33		(38, 39, 48, 49, 51-53, 58, 59, 68, 69)
Impeding patient-clinician relationship	-	9/33		(41, 43, 50, 53, 56, 60, 63, 66, 67)
Undermining holistic surveillance	-	8/33		(37, 39, 40, 43, 56, 60, 63, 66)
Fear or concerns of technology	-	6/33		(37, 44, 45, 49-51)
Level of satisfaction	-/+	6/33		(40, 44, 47, 48, 57, 65)
Influence on decision making process	-/+	5/33		(45, 51, 52, 57, 62)
Attitude towards technology	-/+	5/33		(38, 44, 48, 49, 59)
Characteristics of HCP (IT literacy, age, years of practice)	-/+	4/33		(44, 47, 50, 53)
(Reduce/concerns of) overtreatment of patients	-/+	3/33		(43, 50, 66)
Threat to face-to-face services	-	3/33		(41, 44, 49)
Work-life balance	-/+	2/33		(40, 52)
**Patient**				
Questioning ability of patients	-	9/33		(39, 43, 49-51, 54, 56, 58, 69)
Patient tailored approach	-/+	4/33		(48, 51, 58, 61)
Difficult to engage/maintain use	-	4/33		(39, 40, 44, 58)
Low (health/IT) literacy	-	4/33		(51, 55, 56, 58)
Patient empowerment	+	3/33		(51, 58, 61)
Enabling access for certain groups	-/+	3/33		(40, 51, 62)

For peer review

**Technical**			
Security and confidentiality (issues)	**-/+**	17/33	(37, 45-48, 50, 51, 53, 55, 58, 60-62, 66-69)
Technical issues (access to Wi-Fi, system unreliability)	**-**	12/33	(47, 48, 51, 54-57, 60-63, 67)
(Lack of) usefulness	**-/+**	12/33	(47, 48, 51, 52, 55, 57, 58, 60-62, 65, 67)
(Lack of) usability	**-/+**	11/33	(39, 45, 47, 48, 50, 51, 54, 55, 58, 60, 61)
(Lack of) harmonisation of systems	**-**	10/33	(45, 48, 50, 51, 53-55, 58, 61, 67)
(lack of) technical support	**-/+**	7/33	(39, 51, 53, 56, 61, 62, 64)
Perception of aesthetics/design	**-**	4/33	(48, 51, 65, 67)
Availability of data	**-/+**	4/33	(45, 51, 54, 61)

+ indicates a facilitator, - indicates a barrier, -/+ reported as barrier as well as facilitator, HCP: Health Care Professional. The barrier or facilitator is outlined in this table when reported as barrier or facilitator in all three technologies.

For peer review only

Table 3. Barriers and facilitators of digital technology implementation from the perspective of the HCP, divided per type of technology.

Level of barrier or facilitator	Type of technology		
**Organisational**			
	**Remote consultation (n=27)**	**Remote monitoring (n=23)**	**Digital care platforms (n=16)**
Loss of productivity during implementation		(45)[a]	(45)
Lack of feedback or evaluation	(49, 52)	(49, 52)	
Competitive advantage			(53)
Pressure from other hospitals	(67)	(67)	
Good communication in network	(57)		
Culture that is resistant to change		(51)	
**HCP**			
Sense of isolation	(44, 52)	(52)	
Difficult to establish trust relationship		(68)	
**Patient**			
Concerns of dependency of patients	(43)	(43, 51, 54)	
Patient satisfaction	(40, 44)		(40)
**Technological**			
Proof of utility (by research)		(45)	(45)

*Green background means reported as facilitator. Orange means reported as both facilitator and barrier. [a] The numbers in parentheses denote the corresponding references. RC: Remote consultation, RM: Remote Monitoring, DCP: Digital Care Platform. The barrier or facilitator is reported when reported in less than three technologies, otherwise a barrier or facilitator is reported in the generic table (Table 2).

For peer review only - http://bmjopen.bmj.com/site/about/guidelines.xhtml

Discussion

This systematic umbrella review aimed to investigate the barriers and facilitators of digital health technology in patient care, as perceived by HCPs. Three types of digital health technologies were distinguished: 1) Remote consultation, 2) Remote monitoring, and 3) Digital care platforms. Barriers and facilitators from the perspective of HCP could be categorised as relating to 1) the organisation, 2) the HCPs themselves, 3) the patient, and 4) the technology. Overall, our review highlights that there are still major organisational requirements to be met in order to provide a solid foundation for digital health technology implementation. One way to overcome organisational barriers is by addressing them in the implementation strategy (Figure 2). This includes adequate training and planning ahead for potential safety and confidentiality concerns. In addition, it also includes showing the HCP the eventual benefits such as improved health outcomes by the use of the digital health technology, and ensuring fully embedding within their clinical workflow. The results of this review indicate that the organisational facilitators as perceived by HCPs are comparable to the list of organisational barriers HCPs reported. Thus, these factors act as two sides of the same coin. Major facilitators to support a successful implementation strategy at the organisational level include deploying the right leadership and support, making use of change champions, and ensuring good working relationships. However, as healthcare will continue to face new challenges with regard to technology implementation, barriers and facilitators are not fixed entities that need to be resolved for successful implementation. Instead, the implementation of digital health technologies should be approached as a dynamic process which needs ongoing management and tailoring, taking social relations and context into account (70).

This umbrella review demonstrates that the majority of research in the past years on barriers and facilitators addressed common organisational barriers of technology implementation, such as lack of training, lack of time and resources, an increase in workload, and lack of leadership and support. This substantial attention to organisational issues can be interpreted in two ways. Firstly, that organisational barriers might remain prevalent for implementation, hence the substantial literature

For peer review only - http://bmjopen.bmj.com/site/about/guidelines.xhtml

on them. Alternatively, it suggests that organisational aspects could dominate discussions about implementation of technology, potentially overshadowing other levels on which barriers can take place. For instance, barriers on the level of the individual HCP, such as loss of communication, impeding the patient-physician relationship, undermining holistic surveillance or undermining aspects of professional identity, have been less frequently researched. For instance, a major barrier on the HCP level is loss of communication, specifically pertaining to remote consultation. As a result, HCPs experienced the possibility of misunderstanding the patient, and perceived a decreased personal connection with the patient (56, 60). In addition, HCPs perceived a difference in whether one sees the patient digitally for the first time, or whether one has already established a relationship with the patient. This distinction was particularly reported in reviews implementing digital health technologies during the Coronavirus pandemic, where it became more common to have an initial digital interaction instead of face-to-face consultation (56). In addition, technology could undermine aspects of holistic surveillance, as physical examinations are not possible and it is more difficult to pick up nonverbal cues (56, 63). Despite the HCP perceiving these barriers, patients were found to be satisfied with teleconsultation and remote monitoring and did not experience this loss in communication (56, 60, 63). In this regard, there is a discrepancy between the needs and perspectives of HCPs and patients. The most used explanation on differences in satisfaction levels with regard to video consultation is the inability of HCPs to physically examine the patient (71). While this review primarily focuses on the perspective of HCP, patients are also significant end users of technology. Therefore, it is essential to consider and identify the varying needs of patients in addition to those of HCPs, by conducting more research on the patients' perspectives on the influence of digital technology on their care, and the HCP-patient relationship specifically. Such research may yield more discrepancies, which need to be solved to provide patients with best care without unduly burdening HCP.

This review also indicates the importance of training of professionals at the start of an implementation trajectory. For transformative innovations such as the introduction of a new digital

For peer review only - http://bmjopen.bmj.com/site/about/guidelines.xhtml

health technology, adequate training to facilitate the user's adoption and use of the new technology is essential (72). However, the training needs of HCPs and ideal frequency of training are yet to be defined. Ongoing training, rather than a one-time instruction at the start of the implementation, may be more sustainable when considering potential staff changes (73). The training itself could vary, from practical instruction to hands-on learning, as reported in the study by Dugstad, Eide (74). At the onset, a theoretical approach to training of professionals was used by these authors, but this was soon replaced by practical instruction and training sessions which emphasised capacity building to acquire the necessary skills; such as using the app, handling swipe hand movements, and using software commands (74).

On the HCP level, major barriers included a loss of communication with patients, and the possibility of technology impeding the patient-HCP relationship. Patient connection is at the essence of being a HCP, and has become increasingly difficult in contemporary practice (75). A study conducted in fifty medical departments in the Netherlands showed that healthcare professionals with positive patient relationships were less likely to suffer from patient-related burnout even with high job demands (75). In addition, patient-related burnout was more prevalent among HCPs who had less positive patient relationships (76). In light of these findings, and with the increasing prevalence of burnout among HCPs (77), further research is necessary to investigate whether the use of digital health technology in healthcare actually results in a loss of gratifying interaction with patients.

Patient related barriers, as perceived by HCPs, related to questioning the ability of patients to use digital technology, patient anxiety for using the technology, and low literacy of patients. One of the actions of the *World Health Organisation* on their global strategy is that digital technologies should strengthen health equity by promoting an inclusive digital society (78). As digital health literacy requires skills complementary to health literacy, it is important to ensure that digital health technologies are available for all patients (79). Therefore, understanding and incorporating intended users' needs and context appears to be a prerequisite for effective use of digital health technology

For peer review only - http://bmjopen.bmj.com/site/about/guidelines.xhtml

(80), apparently also from the perspective of HCP. This could be achieved by co-creation, which is described as an interaction in which participants work together to create an outcome that is appreciated by both parties (81). By including HCPs in this process, the possibility that the digital health technologies will meet the needs and requirements of the end user in terms of content and usability will increase; this, in turn, may enhance technology implementation.

Strengths and limitations

The strengths of this study include its comprehensive search on digital health technologies, narrowed down to technologies that include patient interaction, its duplicate approach, and its focus on the end-user. However, the current review was limited by digital technology and policies regulating these being in a state of constant flux. Therefore, barriers and facilitators reported in older studies might be less relevant over time. Presently, we acknowledge that the current state of affairs is undergoing rapid evolution, and consequently, the data of our review may not be entirely up-to-date. However, given that half of the reviews included are less than five years old, we do not expect more recent studies to have major impact on our findings. Another limitation is that some of the included reviews provided little context, making it difficult to determine whether findings were considered facilitators or barriers to technology implementation. In such instances, the reviewers either reached a consensus-based decision or reported a finding both as a barrier and facilitator. Finally, the included reviews differ in terms of the type of technology studied and the disease or condition in which the technology was used. The intention of this review was to provide as complete of an overview as possible of common barriers and facilitators as perceived by HCPs. All technologies in this review include a form of patient interaction, however, barriers or facilitators for a specific technology or implementation may exist.

Conclusions and implications for practice

The implementation of digital health technology in healthcare is influenced by several major barriers from the perspective of the HCP that need to be overcome. These include security and

For peer review only - http://bmjopen.bmj.com/site/about/guidelines.xhtml

confidentiality issues, adequate training, HCPs unfamiliarity with technology, and HCPs questioning the ability of patients to use technology. In order to successfully implement digital health technology, generic barriers and facilitators as well as barriers and facilitators per type of technology must be considered. Firstly, organisational barriers have been frequently reported and remain problematic. However, such barriers may act as facilitators when appropriately addressed in the implementation strategy. Secondly, barriers on both the HCP and patient levels are related to the patient-HCP relationship, the loss of communication, the undermining of holistic surveillance, and questioning the ability of patients. These barriers can be addressed by investigating the needs of the stakeholders involved and by including them as co-creators in the development of digital health technology. By involving HCPs in this process, the digital tool will be more likely to meet end-user requirements in terms of content and usability. In turn, this bolsters the chance for successful implementation. Thirdly, successful implementation of digital health technology demands time and resources. HCP training should be a continuous process to protect against issues related to staff turnover. Lastly, further research is necessary on the other end-user of digital health technology, the patient, in order to understand the role digital health technology occupies in the patient-HCP relationship, and to better understand the needs of this end-user.

Contributorship statement

SJO, KAZ, JJM, EMAS were involved with the design of this review. SJO, SPR, BSJC performed the screening of titles and abstracts of the included studies. With regard to data extraction and synthesis, this was performed by SJO, SPR, BSJC. SJO and SPR prepared the original draft of this review. JJM and EMAS contributed to the refinement. All authors have read and approved the final version.

Competing interests

No competing interest.

For peer review only - http://bmjopen.bmj.com/site/about/guidelines.xhtml

Funding statement

This research received no specific grant from any funding agency in the public, commercial or not-for-profit sectors.

Data sharing statement

All data relevant to the study are included in the article or uploaded as supplementary information

Ethics approval

Not applicable.

Patient consent for publication

Not applicable.

Acknowledgments

The authors wish to thank Dr. L.W.P. Peute, director from the eHealth living and learning lab at the Amsterdam University Medical Center, for her support regarding the tables and figures. Furthermore, the authors would like to express their gratitude to Rebecca Kerstens, from the University of South Australia, for critically assessing the manuscript.

For peer review only

For peer review only - http://bmjopen.bmj.com/site/about/guidelines.xhtml

References

1. Kollerup A, Kjellberg J, Ibsen R. Ageing and health care expenditures: the importance of age per se, steepening of the individual-level expenditure curve, and the role of morbidity. The European Journal of Health Economics. 2022;23(7):1121-49.

2. Gentili A, Failla G, Melnyk A, Puleo V, Tanna GLD, Ricciardi W, Cascini F. The cost-effectiveness of digital health interventions: A systematic review of the literature. Front Public Health. 2022;10:787135.

3. van de Vijver S, Tensen P, Asiki G, Requena-Méndez A, Heidenrijk M, Stronks K, et al. Digital health for all: How digital health could reduce inequality and increase universal health coverage. DIGITAL HEALTH. 2023;9:20552076231185434.

4. Senbekov M, Saliev T, Bukeyeva Z, Almabayeva A, Zhanaliyeva M, Aitenova N, et al. The Recent Progress and Applications of Digital Technologies in Healthcare: A Review. International Journal of Telemedicine and Applications. 2020;2020:8830200.

5. Zorgakkoord I. Samenwerken aan gezonde zorg. Netherlands: Rijksoverheid; 2022.

6. Garavand A, Mohseni M, Asadi H, Etemadi M, Moradi-Joo M, Moosavi A. Factors influencing the adoption of health information technologies: a systematic review. Electronic physician. 2016;8(8):2713.

7. Kraus S, Schiavone F, Pluzhnikova A, Invernizzi AC. Digital transformation in healthcare: Analyzing the current state-of-research. Journal of Business Research. 2021;123:557-67.

8. Tsai CH, Eghdam A, Davoody N, Wright G, Flowerday S, Koch S. Effects of electronic health record implementation and barriers to adoption and use: a scoping review and qualitative analysis of the content. Life. 2020;10(12):327.

9. WHO. WHO guideline: recommendations on digital interventions for health system strengthening. Geneva: World Health Organization; 2019.

10. Smith C. What is wearable tech? Everything you need to know explained: Chris Smith; 2019 [Available from: https://www.wareable.com/wearable-tech/what-is-wearable-tech-753.

11. Tortorella GL, Fogliatto FS, Espôsto KF, Mac Cawley AF, Vassolo R, Tlapa D, Narayanamurthy G. Healthcare costs' reduction through the integration of Healthcare 4.0 technologies in developing economies. Total Quality Management & Business Excellence. 2020:1-21.

12. Shimbo D, Artinian NT, Basile JN, Krakoff LR, Margolis KL, Rakotz MK, et al. Self-measured blood pressure monitoring at home: a joint policy statement from the American Heart Association and American Medical Association. Circulation. 2020;142(4):e42-e63.

For peer review only

For peer review only - http://bmjopen.bmj.com/site/about/guidelines.xhtml

13. Hamine S, Gerth-Guyette E, Faulx D, Green BB, Ginsburg AS. Impact of mHealth chronic disease management on treatment adherence and patient outcomes: a systematic review. Journal of medical Internet research. 2015;17(2):e52.
14. Seoane F, Traver V, Hazelzet J. Value-Driven Digital Transformation in Health and Medical Care. Interactive Process Mining in Healthcare: Springer; 2021. p. 13-26.
15. Crespo-Almendros E, Del Barrio-García S. Online airline ticket purchasing: Influence of online sales promotion type and Internet experience. Journal of Air Transport Management. 2016;53:23-34.
16. Olesch A. Digialisation: a missing connectior or health systems in Europe? Eurohealth. 2022;28(3):24-8.
17. Gopal G, Suter-Crazzolara C, Toldo L, Eberhardt W. Digital transformation in healthcare–architectures of present and future information technologies. Clinical Chemistry and Laboratory Medicine (CCLM). 2019;57(3):328-35.
18. van Gemert-Pijnen LJ, editor Digitalisering in de gezondheidszorg nader beschouwd: eindrapport VWS MEVA. Digitalisering nader beschouwd, opdracht van VWS MEVA; 2022.
19. Manogaran G, Lopez D, Thota C, Abbas KM, Pyne S, Sundarasekar R. Big data analytics in healthcare Internet of Things. Innovative healthcare systems for the 21st century: Springer; 2017. p. 263-84.
20. Smuck M, Odonkor CA, Wilt JK, Schmidt N, Swiernik MA. The emerging clinical role of wearables: factors for successful implementation in healthcare. NPJ Digital Medicine. 2021;4(1):1-8.
21. Yen P-Y, Bakken S. Review of health information technology usability study methodologies. Journal of the American Medical Informatics Association. 2012;19(3):413-22.
22. Shahbaz M, Gao C, Zhai L, Shahzad F, Hu Y. Investigating the adoption of big data analytics in healthcare: the moderating role of resistance to change. Journal of Big Data. 2019;6(1):1-20.
23. Smoll NR, Walker J, Khandaker G. The barriers and enablers to downloading the COVIDSafe app–a topic modelling analysis. Australian and New Zealand Journal of Public Health. 2021;45(4):344-7.
24. Sangers T, Wakkee M, Kramer-Noels E, Nijsten T, Lugtenberg M. Views on mobile health apps for skin cancer screening in the general population: an in-depth qualitative exploration of perceived barriers and facilitators. British Journal of Dermatology. 2021;185(5):961-9.
25. Slevin P, Kessie T, Cullen J, Butler M, Donnelly S, Caulfield B. Exploring the barriers and facilitators for the use of digital health technologies for the management of COPD: a qualitative study of clinician perceptions. QJM: An International Journal of Medicine. 2020;113(3):163-72.

For peer review only - http://bmjopen.bmj.com/site/about/guidelines.xhtml

For peer review only

26. Foong HF, Kyaw BM, Upton Z, Tudor Car L. Facilitators and barriers of using digital technology for the management of diabetic foot ulcers: A qualitative systematic review. International wound journal. 2020;17(5):1266-81.

27. Moher D, Liberati A, Tetzlaff J, Altman DG, Group* P. Preferred reporting items for systematic reviews and meta-analyses: the PRISMA statement. Annals of internal medicine. 2009;151(4):264-9.

28. Rabin BA, Brownson RC. Terminology for dissemination and implementation research2017. 19-45 p.

29. Nilsen P. Making sense of implementation theories, models, and frameworks. Implementation Science 30: Springer; 2020. p. 53-79.

30. Liberati A, Altman DG, Tetzlaff J, Mulrow C, Gøtzsche PC, Ioannidis JP, et al. The PRISMA statement for reporting systematic reviews and meta-analyses of studies that evaluate health care interventions: explanation and elaboration. Journal of clinical epidemiology. 2009;62(10):e1-e34.

31. Hair K, Bahor Z, Macleod M, Liao J, Sena ES. The automated systematic search Deduplicator (ASySD): a rapid, open-source, interoperable tool to remove duplicate citations in biomedical systematic reviews. Biorxiv. 2021.

32. Ouzzani M, Hammady H, Fedorowicz Z, Elmagarmid A. Rayyan-a web and mobile app for systematic reviews. Systematic Reviews, 5, 210. 2016.

33. Richardson E, Aissat D, Williams GA, Fahy N. Keeping what works: remote consultations during the COVID-19 pandemic. Eurohealth. 2020;26(2):73-6.

34. Vegesna A, Tran M, Angelaccio M, Arcona S. Remote patient monitoring via non-invasive digital technologies: a systematic review. Telemedicine and e-Health. 2017;23(1):3-17.

35. Fürstenau D, Auschra C, Klein S, Gersch M. A process perspective on platform design and management: evidence from a digital platform in health care. Electronic Markets. 2019;29(4):581-96.

36. Aromataris E, Fernandez R, Godfrey CM, Holly C, Khalil H, Tungpunkom P. Summarizing systematic reviews: methodological development, conduct and reporting of an umbrella review approach. JBI Evidence Implementation. 2015;13(3):132-40.

37. Antoun J. Electronic mail communication between physicians and patients: a review of challenges and opportunities. Fam Pract. 2016;33(2):121-6.

38. Hassibian MR, Hassibian S. Telemedicine acceptance and implementation in developing countries: Benefits, categories, and barriers. Razavi International Journal of Medicine. 2016;4(3).

For peer review only - http://bmjopen.bmj.com/site/about/guidelines.xhtml

39. Almathami HKY, Win KT, Vlahu-Gjorgievska E. Barriers and Facilitators That Influence Telemedicine-Based, Real-Time, Online Consultation at Patients' Homes: Systematic Literature Review. J Med Internet Res. 2020;22(2):e16407.

40. Appleton R, Williams J, San Juan NV, Needle JJ, Schlief M, Jordan H, et al. Implementation, adoption, and perceptions of telemental health during the COVID-19 pandemic: systematic review. Journal of medical Internet research. 2021;23(12):e31746.

41. Batterham PJ, Sunderland M, Calear AL, Davey CG, Christensen H, Teesson M, et al. Developing a roadmap for the translation of e-mental health services for depression. Aust N Z J Psychiatry. 2015;49(9):776-84.

42. Bokolo AJ. Exploring the adoption of telemedicine and virtual software for care of outpatients during and after COVID-19 pandemic. Ir J Med Sci. 2021;190(1):1-10.

43. Brunton L, Bower P, Sanders C. The Contradictions of Telehealth User Experience in Chronic Obstructive Pulmonary Disease (COPD): A Qualitative Meta-Synthesis. PLoS One. 2015;10(10):e0139561.

44. Cowan KE, McKean AJ, Gentry MT, Hilty DM. Barriers to Use of Telepsychiatry: Clinicians as Gatekeepers. Mayo Clinic Proceedings. 2019;94(12):2510-23.

45. de Grood C, Raissi A, Kwon Y, Santana MJ. Adoption of e-health technology by physicians: a scoping review. J Multidiscip Healthc. 2016;9:335-44.

46. De Guzman KR, Snoswell CL, Taylor ML, Senanayake B, Haydon HM, Batch JA, et al. A Systematic Review of Pediatric Telediabetes Service Models. Diabetes Technol Ther. 2020;22(8):623-38.

47. Finkelstein J, Knight A, Marinopoulos S, Gibbons MC, Berger Z, Aboumatar H, et al. Enabling patient-centered care through health information technology. Evid Rep Technol Assess (Full Rep). 2012(206):1-1531.

48. Gagnon MP, Ngangue P, Payne-Gagnon J, Desmartis M. m-Health adoption by healthcare professionals: a systematic review. Journal of the American Medical Informatics Association. 2016;23(1):212-20.

49. Greenhalgh T, A'Court C, Shaw S. Understanding heart failure; explaining telehealth - a hermeneutic systematic review. BMC Cardiovasc Disord. 2017;17(1):156.

50. Hopstaken JS, Verweij L, van Laarhoven CJ, Blijlevens NM, Stommel MW, Hermens RP. Effect of digital care platforms on quality of care for oncological patients and barriers and facilitators for their implementation: Systematic review. Journal of Medical Internet Research. 2021;23(9):e28869.

For peer review only

51. Jacob C, Sanchez-Vazquez A, Ivory C. Social, Organizational, and Technological Factors Impacting Clinicians' Adoption of Mobile Health Tools: Systematic Literature Review. Jmir Mhealth and Uhealth. 2020;8(2).

52. Lewinski AA, Rushton S, Van Voorhees E, Boggan JC, Whited JD, Shoup JP, et al. Implementing remote triage in large health systems: A qualitative evidence synthesis. Res Nurs Health. 2021;44(1):138-54.

53. Li J, Talaei-Khoei A, Seale H, Ray P, Macintyre CR. Health Care Provider Adoption of eHealth: Systematic Literature Review. Interact J Med Res. 2013;2(1):e7.

54. Liyanage-Don N, Fung D, Phillips E, Kronish IM. Implementing Home Blood Pressure Monitoring into Clinical Practice. Current Hypertension Reports. 2019;21(2).

55. Maclure K, Stewart D, Strath A. A systematic review of medical and non-medical practitioners' views of the impact of ehealth on shared care. European Journal of Hospital Pharmacy. 2014;21(1):54-62.

56. Meng G, McAiney C, Perlman CM, McKillop I, Tisseverasinghe T, Chen HH. Service process factors affecting patients' and clinicians' experiences on rapid teleconsultation implementation in out-patient neurology services during COVID-19 pandemic: a scoping review. BMC health services research. 2022;22(1):1-17.

57. Metzger G, Jatana K, Apfeld J, Deans KJ, Minneci PC, Halaweish I. State of telemedicine use in pediatric surgery in the USA-where we stand and what we can gain from the COVID-19 pandemic: A scoping review. World Journal of Pediatric Surgery. 2021;4(1).

58. Mileski M, Kruse CS, Catalani J, Haderer T. Adopting Telemedicine for the Self-Management of Hypertension: Systematic Review. JMIR Med Inform. 2017;5(4):e41.

59. Muir SD, de Boer K, Nedeljkovic M, Meyer D. Barriers and facilitators of videoconferencing psychotherapy implementation in veteran mental health care environments: a systematic review. BMC Health Serv Res. 2020;20(1):999.

60. O'Cathail M, Sivanandan MA, Diver C, Patel P, Christian J. The Use of Patient-Facing Teleconsultations in the National Health Service: Scoping Review. JMIR Med Inform. 2020;8(3):e15380.

61. Palacholla RS, Fischer N, Coleman A, Agboola S, Kirley K, Felsted J, et al. Provider- and Patient-Related Barriers to and Facilitators of Digital Health Technology Adoption for Hypertension Management: Scoping Review. JMIR Cardio. 2019;3(1):e11951.

62. Slater H, Campbell JM, Stinson JN, Burley MM, Briggs AM. End User and Implementer Experiences of mHealth Technologies for Noncommunicable Chronic Disease Management in Young Adults: Systematic Review. J Med Internet Res. 2017;19(12):e406.

For peer review only - http://bmjopen.bmj.com/site/about/guidelines.xhtml

63. Smith SM, Jacobsen JHW, Atlas AP, Khoja A, Kovoor JG, Tivey DR, et al. Telehealth in surgery: an umbrella review. ANZ journal of surgery. 2021;91(11):2360-75.

64. Varsi C, Solberg Nes L, Kristjansdottir OB, Kelders SM, Stenberg U, Zangi HA, et al. Implementation Strategies to Enhance the Implementation of eHealth Programs for Patients With Chronic Illnesses: Realist Systematic Review. J Med Internet Res. 2019;21(9):e14255.

65. Vedel I, Akhlaghpour S, Vaghefi I, Bergman H, Lapointe L. Health information technologies in geriatrics and gerontology: a mixed systematic review. Journal of the American Medical Informatics Association. 2013;20(6):1109-19.

66. Vyas KS, Hambrick HR, Shakir A, Morrison SD, Tran DC, Pearson K, et al. A Systematic Review of the Use of Telemedicine in Plastic and Reconstructive Surgery and Dermatology. Ann Plast Surg. 2017;78(6):736-68.

67. Whitelaw S, Pellegrini DM, Mamas MA, Cowie M, Van Spall HGC. Barriers and facilitators of the uptake of digital health technology in cardiovascular care: a systematic scoping review. Eur Heart J Digit Health. 2021;2(1):62-74.

68. Xyrichis A, Iliopoulou K, Mackintosh NJ, Bench S, Terblanche M, Philippou J, Sandall J. Healthcare stakeholders' perceptions and experiences of factors affecting the implementation of critical care telemedicine (CCT): qualitative evidence synthesis. Cochrane Database Syst Rev. 2021;2(2):Cd012876.

69. Zaman SB, Khan RK, Evans RG, Thrift AG, Maddison R, Islam SMS. Exploring Barriers to and Enablers of the Adoption of Information and Communication Technology for the Care of Older Adults With Chronic Diseases: Scoping Review. JMIR aging. 2022;5(1):e25251.

70. Checkland K, Harrison S, Marshall M. Is the metaphor of'barriers to change'useful in understanding implementation? Evidence from general medical practice. Journal of health services research & policy. 2007;12(2):95-100.

71. Zandbelt LC, de Kanter FEC, Ubbink DT. E-consulting in a medical specialist setting: Medicine of the future? Patient Education and Counseling. 2016;99(5):689-705.

72. Gallouj F, Weinstein O. Innovation in services. Research policy. 1997;26(4-5):537-56.

73. Versluis A, van Luenen S, Meijer E, Honkoop PJ, Pinnock H, Mohr DC, et al. SERIES: eHealth in primary care. Part 4: addressing the challenges of implementation. European Journal of General Practice. 2020;26(1):140-5.

74. Dugstad J, Eide T, Nilsen ER, Eide H. Towards successful digital transformation through co-creation: a longitudinal study of a four-year implementation of digital monitoring technology in residential care for persons with dementia. BMC Health Serv Res. 2019;19(1):366.

For peer review only

For peer review only - http://bmjopen.bmj.com/site/about/guidelines.xhtml

75. Scheepers R, Silkens M, van den Berg J, Lombarts K. Associations between job demands, job resources and patient-related burnout among physicians: results from a multicentre observational study. BMJ Open. 2020;10(9):e038466.

76. Shanafelt TD. Enhancing meaning in work: a prescription for preventing physician burnout and promoting patient-centered care. Jama. 2009;302(12):1338-40.

77. Hiver C, Villa A, Bellagamba G, Lehucher-Michel MP. Burnout prevalence among European physicians: a systematic review and meta-analysis. Int Arch Occup Environ Health. 2022;95(1):259-73.

78. Organization WH. Global strategy on digital health 2020-2025. 2021.

79. Smith B, Magnani JW. New technologies, new disparities: The intersection of electronic health and digital health literacy. International Journal of Cardiology. 2019;292:280-2.

80. Thies K, Anderson D, Cramer B. Lack of Adoption of a Mobile App to Support Patient Self-Management of Diabetes and Hypertension in a Federally Qualified Health Center: Interview Analysis of Staff and Patients in a Failed Randomized Trial. JMIR Hum Factors. 2017;4(4):e24.

81. Prahalad CK, Ramaswamy V. Co-creation experiences: The next practice in value creation. Journal of interactive marketing. 2004;18(3):5-14.

For peer review only

For peer review only - http://bmjopen.bmj.com/site/about/guidelines.xhtml

Figure Legend

Figure 1 - Flowchart of the article selection.

Figure 2 - Top three barriers or facilitators on each level of digital health implementation, and practical implications or solutions.

For peer review only

For peer review only - http://bmjopen.bmj.com/site/about/guidelines.xhtml

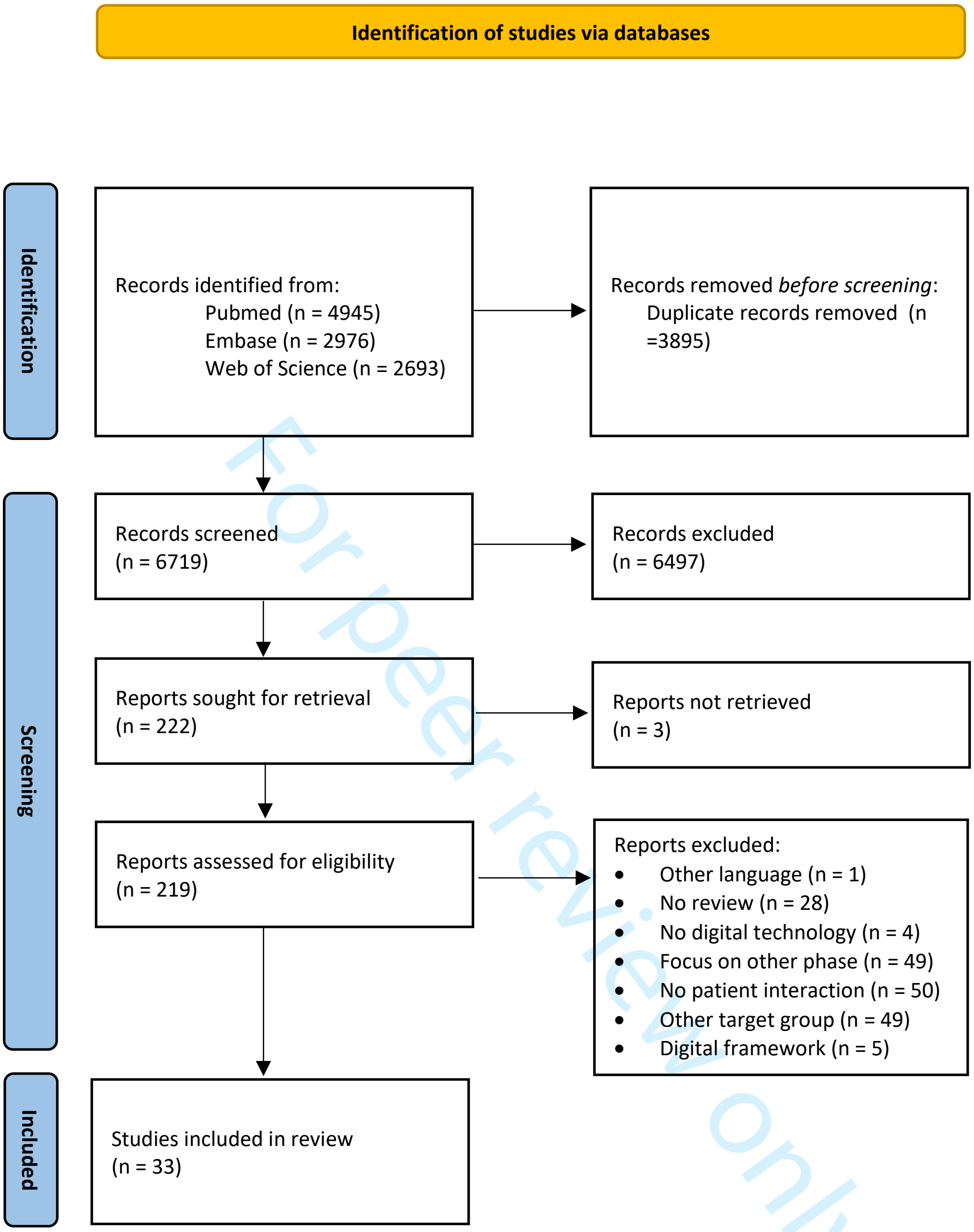

Figure 1. Flowchart of the article selection

For peer review only

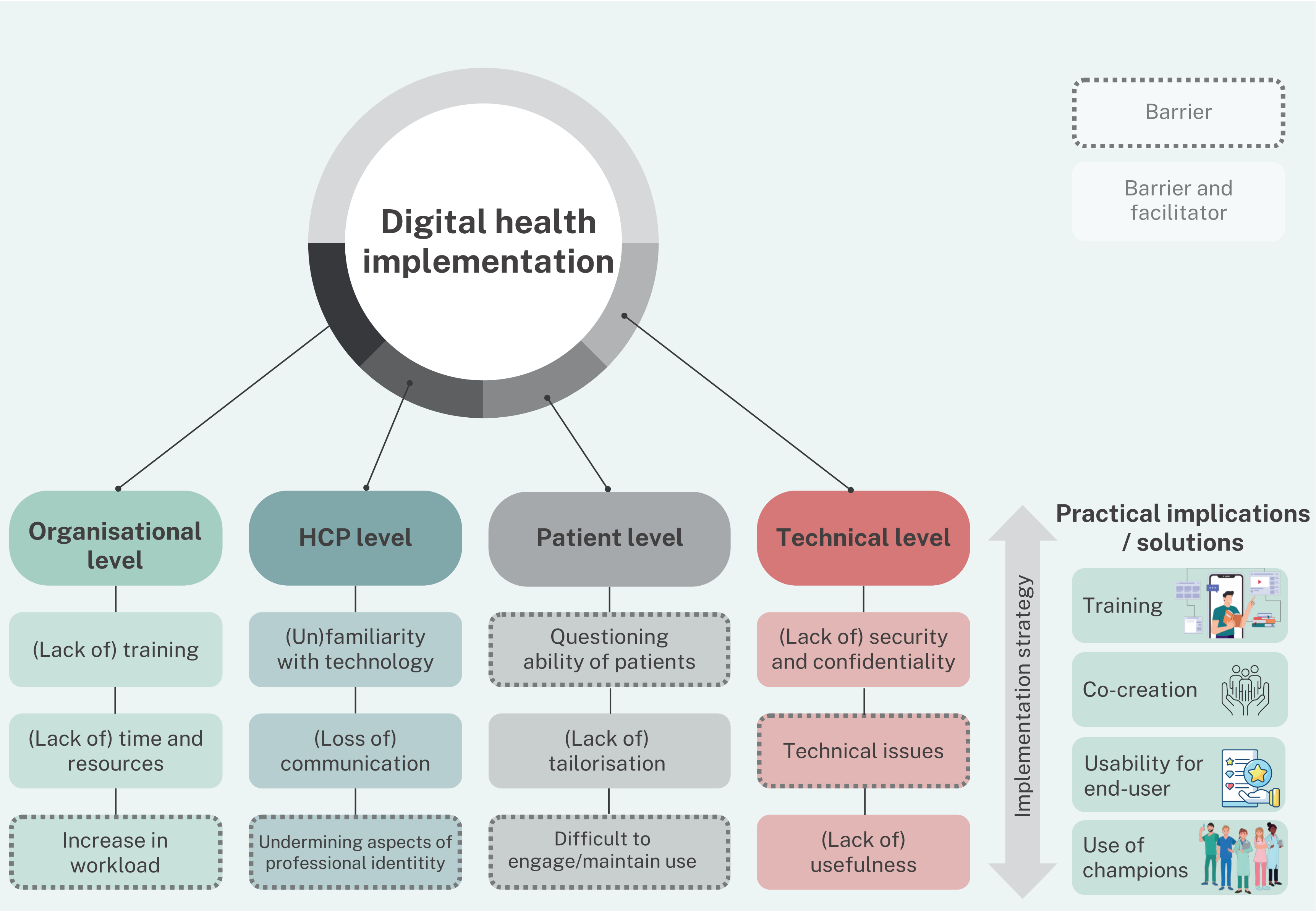
Digital health implementation
Barrier
Barrier and facilitator
Organisational level
HCP level
Patient level
Technical level
(Lack of) training
(Lack of) time and resources
Increase in workload
(Un)familiarity with technology
(Loss of) communication
Undermining aspects of professional identitity
Questioning ability of patients
(Lack of) tailorisation
Difficult to engage/maintain use
(Lack of) security and confidentiality
Technical issues
(Lack of) usefulness
Implementation strategy
Practical implications / solutions
Training
Co-creation
Usability for end-user
Use of champions

Supplementary material

Supplementary Table 1. In- and exclusion criteria

Inclusion criteria	Exclusion criteria
• Publication type: systematic review, meta-analysis, literature review. At least when systematic review was mentioned in title or abstract.<br>• Describing barriers, facilitators, successes, inhibitions, failures for the adoption, implementation, maintenance of technology in practice.<br>• From the healthcare professional perspective: medical specialists, nurse practitioners, and physician assistants.	• Articles written in other language than Dutch, German, or English<br>• Publication type: research protocol, empirical studies, opinion papers, conference abstract.<br>• No digital technology described<br>• Focus on other phase than implementation, adoption, maintenance phase of digital technology<br>• No patient interaction with the technology<br>• Different target group (managerial, patient, primary care, nurse).<br>• Article describes a framework related to technology implementation<br>• No barriers or facilitators described

For peer review only

For peer review only - http://bmjopen.bmj.com/site/about/guidelines.xhtml

Supplementary Table 2. PICO with original PubMed search and results (and translated to Embase and Web of Science)

PICO*	Participants	Intervention	Context	Outcome
Research question	Medical specialists, nurse practitioners, physician assistants involved with digital technology	Digital technology based on patient interaction	Implementation, adoption, maintenance of technology in healthcare	Barriers and facilitators
PubMed	"Physicians"[Mesh] OR "Physician Assistants"[Mesh] OR "Nurse Practitioners"[Mesh] "Attitude of health personnel"[Mesh] OR "Allergist*"[tiab] OR "Anesthesiologist*"[tiab] OR "Anaesthesiologist*"[tiab] OR "Cardiologist*"[tiab] OR "Dermatologist*"[tiab] OR "Doctor*"[tiab] OR "Endocrinologist*"[tiab] OR "Gastroenterologist*"[tiab] OR "Geriatrician*"[tiab] OR "Geriatrist*"[tiab] OR "Gerontologist*"[tiab] OR "Gynaecologist*" OR "Gynecologist*"[tiab] OR "Hematologist*"[tiab] OR "Hepatologist*"[tiab] OR "Hospitalist*"[tiab] OR "Immunologist*"[tiab] OR "Infectious disease specialist*"[tiab] OR "Internist*"[tiab] OR "Intensivist*"[tiab] OR "Medical geneticist*"[tiab] OR "Medical microbiologist*"[tiab] OR "Medical specialist*"[tiab] OR "Nephrologist*"[tiab] OR "Neurologist*"[tiab] OR "Obstetrician*"[tiab] OR "Oncologist*"[tiab] OR "Ophthalmologist*"[tiab] OR "Orthopedic specialist*"[tiab] OR "Otolaryngologist*"[tiab] OR "Pathologist*"[tiab] OR "Paediatrician*"[tiab] OR "Pediatrician*"[tiab] OR "Physician*"[tiab] OR "Neonatologist*"[tiab] OR "Neurosurgeon*"[tiab] OR "Pathologist*"[tiab] OR "Physiatrist*"[tiab] OR "Psychiatrist*"[tiab] OR "Physician*"[tiab] OR "Pulmonologist*"[tiab] OR "Radiologist*"[tiab] OR "Radiotherapist*"[tiab] OR "Rheumatologist*"[tiab] OR "Surgeon*"[tiab] OR "Neurosurgeon*"[tiab] OR "Urologist*"[tiab] OR "nurse practitioner*"[tiab]	"Telemedicine"[Mesh] OR "User-Computer Interface"[Mesh] OR "Multimedia"[Mesh] OR "Cell phones"[Mesh] OR "Public health informatics"[Mesh] OR "Medical informatics"[Mesh] OR "Computers, handheld"[Mesh] OR "Mobile Applications"[Mesh] OR "Internet"[Mesh] OR "Patient Portals"[Mesh] OR "econsult*"[tiab] OR "e-consult*"[tiab] OR "ediagnos*"[tiab] OR "e diagnos*"[tiab] OR "mobile health*"[tiab] OR "mhealth*"[tiab] OR "m health*"[tiab] OR "telehealth*"[tiab] OR "tele health"[tiab] OR "remote consult*"[tiab] OR "teleconsult*"[tiab] OR "tele consult*"[tiab] OR "telediagnos*"[tiab] OR "tele diagnos*"[tiab] OR "telemedic*"[tiab] OR "tele medic*"[tiab] OR "telemonitor*"[tiab] OR "tele monitor*"[tiab] OR "ehealth*"[tiab] OR "e-health*"[tiab] OR "telecare"[tiab] OR "tele care"[tiab] OR "digital health"[tiab] OR "app"[tiab] OR "apps"[tiab] OR "smartphone*"[tiab] OR "phone application*"[tiab] OR "telephone application*"[tiab] OR "mobile application*"[tiab] OR "mobile technolog*"[tiab] OR "health technolog*"[tiab] OR "health application*"[tiab] OR "internet*"[tiab] OR "world wide web*"[tiab] OR "webportal*"[tiab] OR "web portal*"[tiab] OR "patient portal*"[tiab] OR "ipad"[tiab] OR "ipads"[tiab] OR "telecounsel*"[tiab] OR "tele counsel*"[tiab] OR "remote counsel*"[tiab] OR "distance consult*"[tiab] OR "distance counsel*"[tiab] OR "distant consult*"[tiab] OR "patient monitoring"[tiab] OR "interactive voice response*"[tiab] OR "multimedia"[tiab] OR "Mhapps"[tiab] OR "e-coach*"[tiab] OR "wearable*"[tiab] OR "handheld"[tiab] OR "virtual patient*"[tiab] OR "computer"[tiab] OR "digital*"[tiab]	"Organisational Innovation"[Mesh] OR "organisational chang*"[tiab] OR "organisational chang*"[tiab] OR "organisational innovat*"[tiab] OR "organisational innovat*"[tiab] OR "implement*"[tiab] OR "adopt*"[tiab] OR "transform*"[tiab]	No search terms included

* The C of Comparison is replaced to Context, since we did not look for studies with controlled conditions (placebo/no digital technology). Search terms between columns were combined with AND; the search is limited to publication type [systematic reviews, literature reviews and meta-analysis].

For peer review only - http://bmjopen.bmj.com/site/about/guidelines.xhtml

Supplementary Table 2. PubMed Results (17 June 2022)

Search	PubMed Query – June 17, 2022	Results
#5	#1 AND #2 AND #3 AND #4	4,945
#4	(("Review Literature as Topic"[Mesh] OR "Review"[Publication Type] OR "Meta-Analysis as Topic"[Mesh] OR "review*"[tiab] OR "meta-analys*"[tiab] OR "Meta-Analysis "[Publication Type] OR systematic[sb]) NOT ("Letter"[Publication Type] OR "Editorial"[Publication Type] OR "Comment"[Publication Type]))	4,149,865
#3	"Organizational Innovation"[Mesh] OR "organizational chang*"[tiab] OR "organisational chang*"[tiab] OR "organizational innovat*"[tiab] OR "organisational innovat*"[tiab] OR "implement*"[tiab] OR "adopt*"[tiab] OR "transform*"[tiab]	1,458,582
#2	"Physicians"[Mesh] OR "Physician Assistants"[Mesh] OR "Nurse Practitioners"[Mesh] "Attitude of health personnel"[Mesh] OR "Allergist*"[tiab] OR "Anesthesiologist*"[tiab] OR "Anaesthesiologist*"[tiab] OR "Cardiologist*"[tiab] OR "Dermatologist*"[tiab] OR "Doctor*"[tiab] OR "Endocrinologist*"[tiab] OR "Gastroenterologist*"[tiab] OR "Geriatrician*"[tiab] OR "Geriatrist*"[tiab] OR "Gerontologist*"[tiab] OR "Gynaecologist*" OR "Gynecologist*"[tiab] OR "Hematologist*"[tiab] OR "Hepatologist*"[tiab] OR "Hospitalist*"[tiab] OR "Immunologist*"[tiab] OR "Infectious disease specialist*"[tiab] OR "Internist*"[tiab] OR "Intensivist*"[tiab] OR "Medical geneticist*"[tiab] OR "Medical microbiologist*"[tiab] OR "Medical specialist*"[tiab] OR "Nephrologist*"[tiab] OR "Neurologist*"[tiab] OR "Obstetrician*"[tiab] OR "Oncologist*"[tiab] OR "Ophthalmologist*"[tiab] OR "Orthopedic specialist*"[tiab] OR "Otolaryngologist*"[tiab] OR "Pathologist*"[tiab] OR "Paediatrician*"[tiab] OR "Pediatrician*"[tiab] OR "Physician*"[tiab] OR "Neonatologist*"[tiab] OR "Neurosurgeon*"[tiab] OR "Pathologist*"[tiab] OR "Physiatrist*"[tiab] OR "Psychiatrist*"[tiab] OR "Physician*"[tiab] OR "Pulmonologist*"[tiab] OR "Radiologist*"[tiab] OR "Radiotherapist*"[tiab] OR "Rheumatologist*"[tiab] OR "Surgeon*"[tiab] OR "Neurosurgeon*"[tiab] OR "Urologist*"[tiab] OR "nurse practitioner*"[tiab] OR "clinician*"[tiab]	1,297,435
#1	"Telemedicine"[Mesh] OR "User-Computer Interface"[Mesh] OR "Multimedia"[Mesh] OR "Cell phones"[Mesh] OR "Public health informatics"[Mesh] OR "Medical informatics"[Mesh] OR "Nursing informatics"[Mesh] OR "Computers, handheld"[Mesh] OR "Mobile Applications"[Mesh] OR "Internet"[Mesh] OR "Patient Portals"[Mesh] OR "econsult*"[tiab] OR "e-consult*"[tiab] OR "ediagnos*"[tiab] OR "e diagnos*"[tiab] OR "mobile health*"[tiab] OR "mhealth*"[tiab] OR "m health*"[tiab] OR "telehealth*"[tiab] OR "tele health"[tiab] OR "remote consult*"[tiab] OR "teleconsult*"[tiab] OR "tele consult*"[tiab] OR "telediagnos*"[tiab] OR "tele diagnos*"[tiab] OR "telemedic*"[tiab] OR "tele medic*"[tiab] OR "telemonitor*"[tiab] OR "tele monitor*"[tiab] OR "ehealth*"[tiab] OR "e-health*"[tiab] OR "telecare"[tiab] OR "tele care"[tiab] OR "digital health"[tiab] OR "app"[tiab] OR "apps"[tiab] OR "smartphone*"[tiab] OR "phone application*"[tiab] OR "telephone application*"[tiab] OR "mobile application*"[tiab] OR "mobile technolog*"[tiab] OR "health technolog*"[tiab] OR "health application*"[tiab] OR "internet*"[tiab] OR "world wide web*"[tiab] OR "webportal*"[tiab] OR "web portal*"[tiab] OR "patient portal*"[tiab] OR "ipad"[tiab] OR "ipads"[tiab] OR "telecounsel*"[tiab] OR "tele counsel*"[tiab] OR "remote counsel*"[tiab] OR "distance consult*"[tiab] OR "distance counsel*"[tiab] OR "distant consult*"[tiab] OR "patient monitoring"[tiab] OR "interactive voice response*"[tiab] OR "multimedia"[tiab] OR "Mhapps"[tiab] OR "e-coach*"[tiab] OR "wearable*"[tiab] OR "handheld"[tiab] OR "virtual patient*"[tiab] OR "computer"[tiab] OR "digital*"[tiab]	1,098,834

For peer review only - http://bmjopen.bmj.com/site/about/guidelines.xhtml

Supplementary Table 2. Embase.com Results (17 June 2022)

Search	Embase Query – June 17, 2022	Results
#5	#1 AND #2 AND #3 AND #4	2,976
#4	('systematic review'/exp OR 'meta analysis'/exp OR 'review*':ab,ti,kw OR 'meta-analys*':ab,ti,kw) NOT ('conference abstract'/it OR 'conference review'/it OR 'editorial'/it OR 'erratum'/it OR 'letter'/it OR 'note'/it OR 'short survey'/it)	2,739,615
#3	'organizational chang*':ti,ab,kw OR 'organisational chang*':ti,ab,kw OR 'organizational innovat*':ti,ab,kw OR 'organisational innovat*':ti,ab,kw OR 'implement*':ti,ab,kw OR 'adopt*':ti,ab,kw OR 'transform*':ti,ab,kw	1,776,391
#2	'physician'/exp OR 'physician assistant'/exp OR 'nurse practitioner'/exp OR 'allergist*':ti,ab,kw OR 'anesthesiologist*':ti,ab,kw OR 'anaesthesiologist*':ti,ab,kw OR 'cardiologist*':ti,ab,kw OR 'dermatologist*':ti,ab,kw OR 'doctor*':ti,ab,kw OR 'endocrinologist*':ti,ab,kw OR 'gastroenterologist*':ti,ab,kw OR 'geriatrician*':ti,ab,kw OR 'geriatrist*':ti,ab,kw OR 'gerontologist*':ti,ab,kw OR 'gynaecologist*':ti,ab,kw OR 'gynecologist*':ti,ab,kw OR 'hematologist*':ti,ab,kw OR 'hepatologist*':ti,ab,kw OR 'hospitalist*':ti,ab,kw OR 'immunologist*':ti,ab,kw OR 'infectious disease specialist*':ti,ab,kw OR 'internist*':ti,ab,kw OR 'intensivist*':ti,ab,kw OR 'medical geneticist*':ti,ab,kw OR 'medical microbiologist*':ti,ab,kw OR 'medical specialist*':ti,ab,kw OR 'nephrologist*':ti,ab,kw OR 'neurologist*':ti,ab,kw OR 'obstetrician*':ti,ab,kw OR 'oncologist*':ti,ab,kw OR 'ophthalmologist*':ti,ab,kw OR 'orthopedic specialist*':ti,ab,kw OR 'otolaryngologist*':ti,ab,kw OR 'paediatrician*':ti,ab,kw OR 'pediatrician*':ti,ab,kw OR 'neonatologist*':ti,ab,kw OR 'pathologist*':ti,ab,kw OR 'physiatrist*':ti,ab,kw OR 'psychiatrist*':ti,ab,kw OR 'physician*':ti,ab,kw OR 'pulmonologist*':ti,ab,kw OR 'radiologist*':ti,ab,kw OR 'radiotherapist*':ti,ab,kw OR 'rheumatologist*':ti,ab,kw OR 'surgeon*':ti,ab,kw OR 'neurosurgeon*':ti,ab,kw OR 'urologist*':ti,ab,kw OR 'nurse practitioner*':ti,ab,kw OR 'clinician*':ti,ab,kw	2,201,667
#1	'telehealth'/de OR 'telemedicine'/de OR 'telemonitoring'/exp OR 'internet'/exp OR 'computer interface'/exp OR 'multimedia'/exp OR 'mobile phone'/exp OR 'microcomputer'/exp OR 'mobile application'/exp OR 'medical informatics'/exp OR 'econsult*':ti,ab,kw OR 'e-consult*':ti,ab,kw OR 'ediagnos*':ti,ab,kw OR 'e diagnos*':ti,ab,kw OR 'mobile health*':ti,ab,kw OR 'mhealth*':ti,ab,kw OR 'm health*':ti,ab,kw OR 'telehealth*':ti,ab,kw OR 'tele health':ti,ab,kw OR 'remote consult*':ti,ab,kw OR 'teleconsult*':ti,ab,kw OR 'tele consult*':ti,ab,kw OR 'telediagnos*':ti,ab,kw OR 'tele diagnos*':ti,ab,kw OR 'telemedic*':ti,ab,kw OR 'tele medic*':ti,ab,kw OR 'telemonitor*':ti,ab,kw OR 'tele monitor*':ti,ab,kw OR 'ehealth*':ti,ab,kw OR 'e-health*':ti,ab,kw OR 'telecare':ti,ab,kw OR 'tele care':ti,ab,kw OR 'digital health':ti,ab,kw OR 'app':ti,ab,kw OR 'apps':ti,ab,kw OR 'smartphone*':ti,ab,kw OR 'phone application*':ti,ab,kw OR 'telephone application*':ti,ab,kw OR 'mobile application*':ti,ab,kw OR 'mobile technolog*':ti,ab,kw OR 'health technolog*':ti,ab,kw OR 'health application*':ti,ab,kw OR 'internet*':ti,ab,kw OR 'world wide web*':ti,ab,kw OR 'webportal*':ti,ab,kw OR 'web portal*':ti,ab,kw OR 'patient portal*':ti,ab,kw OR 'ipad':ti,ab,kw OR 'ipads':ti,ab,kw OR 'telecounsel*':ti,ab,kw OR 'tele counsel*':ti,ab,kw OR 'remote counsel*':ti,ab,kw OR 'distance consult*':ti,ab,kw OR 'distance counsel*':ti,ab,kw OR 'distant consult*':ti,ab,kw OR 'patient monitoring':ti,ab,kw OR 'interactive voice response*':ti,ab,kw OR 'multimedia':ti,ab,kw OR 'mhapps':ti,ab,kw OR 'e-coach*':ti,ab,kw OR 'wearable*':ti,ab,kw OR 'handheld':ti,ab,kw OR 'virtual patient*':ti,ab,kw OR 'computer':ti,ab,kw OR 'digital*':ti,ab,kw	889,047

For peer review only

Supplementary Table 2. Clarivate Analytics/Web of Science Core Collection Results (17 June 2022)

Search	Web of Science Query – June 17, 2022	Results
#5	#1 AND #2 AND #3 AND #4	2,693
#4	TS=("review*" OR "meta-analys*")	3,174,557
#3	TS=("organizational chang*" OR "organisational chang*" OR "organizational innovat*" OR "organisational innovat*" OR "implement*" OR "adopt*" OR "transform*")	3,965,854
#2	TS=("Allergist*" OR "Anesthesiologist*" OR "Anaesthesiologist*" OR "Cardiologist*" OR "Dermatologist*" OR "Doctor*" OR "Endocrinologist*" OR "Gastroenterologist*" OR "Geriatrician*" OR "Geriatrist*" OR "Gerontologist*" OR "Gynaecologist*" OR "Gynecologist*" OR "Hematologist*" OR "Hepatologist*" OR "Hospitalist*" OR "Immunologist*" OR "Infectious disease specialist*" OR "Internist*" OR "Intensivist*" OR "Medical geneticist*" OR "Medical microbiologist*" OR "Medical specialist*" OR "Nephrologist*" OR "Neurologist*" OR "Obstetrician*" OR "Oncologist*" OR "Ophthalmologist*" OR "Orthopedic specialist*" OR "Otolaryngologist*" OR "Pathologist*" OR "Paediatrician*" OR "Pediatrician*" OR "Physician*" OR "Neonatologist*" OR "Neurosurgeon*" OR "Pathologist*" OR "Physiatrist*" OR "Psychiatrist*" OR "Physician*" OR "Pulmonologist*" OR "Radiologist*" OR "Radiotherapist*" OR "Rheumatologist*" OR "Surgeon*" OR "Neurosurgeon*" OR "Urologist*" OR "nurse practitioner*" OR "clinician*")	1,227,838
#1	TS=("econsult*" OR "e-consult*" OR "ediagnos*" OR "e diagnos*" OR "mobile health*" OR "mhealth*" OR "m health*" OR "telehealth*" OR "tele health" OR "remote consult*" OR "teleconsult*" OR "tele consult*" OR "telediagnos*" OR "tele diagnos*" OR "telemedic*" OR "tele medic*" OR "telemonitor*" OR "tele monitor*" OR "ehealth*" OR "e-health*" OR "telecare" OR "tele care" OR "digital health" OR "app" OR "apps" OR "smartphone*" OR "phone application*" OR "telephone application*" OR "mobile application*" OR "mobile technolog*" OR "health technolog*" OR "health application*" OR "internet*" OR "world wide web*" OR "webportal*" OR "web portal*" OR "patient portal*" OR "ipad" OR "ipads" OR "telecounsel*" OR "tele counsel*" OR "remote counsel*" OR "distance consult*" OR "distance counsel*" OR "distant consult*" OR "patient monitoring" OR "interactive voice response*" OR "multimedia" OR "Mhapps" OR "e-coach*" OR "wearable*" OR "handheld" OR "virtual patient*" OR "computer" OR "digital*")	1,564,093

For peer review only

For peer review only - http://bmjopen.bmj.com/site/about/guidelines.xhtml

Supplementary Table 3. Quality assessment of included studies (n=33)

**Author (year)**	**Q1** Is the review question clearly and explicitly stated?	**Q2** Were the inclusion criteria appropriate for the review question?	**Q3** Was the search strategy appropriate?	**Q4** Were the sources and resources used to search for studies adequate?	**Q5** Were the criteria for appraising the studies appropriate?[1]	**Q6** Was the critical appraisal conducted by two or more reviewers independently?[1]	**Q7** Were methods implemented to minimise errors in data extraction?	**Q8** Were the methods used to combine studies appropriate?[1,2]	**Q9** Was the likelihood of publication bias assessed?[1]	**Q10** Were recommendations for policy and/or practice supported by the reported data?	**Q11** Were the specific drivers for new research appropriate?
Almathami, Win and Vlahu-Gjorgievska (1)	Yes	Yes	Yes	Yes	Yes	**Unclear**	Yes	Yes	No	Yes	**Unclear**
Antoun (2)	Yes	**Unclear**	**Unclear**	**Unclear**	NA*	NA	**Unclear**	NA	NA	Yes	Yes
Appleton, Williams (3)	Yes	Yes	Yes	Yes	Yes	Yes	Yes	Yes	NA	Yes	Yes
Batterham, Sunderland (4)	Yes	Yes	**Unclear**	Yes	**Unclear**	**Unclear**	**Unclear**	Yes	NA	Yes	Yes
Bokolo (5)	Yes	Yes	Yes	Yes	NA	NA	**Unclear**	Yes	NA	Yes	Yes
Brunton, Bower and Sanders (6)	Yes	Yes	Yes	Yes	Yes	Yes	Yes	Yes	NA	Yes	Yes
Cowan, McKean (7)	Yes	**Unclear**	Yes	No	NA	NA	**Unclear**	Yes	NA	Yes	Yes
de Grood, Raissi (8)	Yes	Yes	Yes	Yes	NA	NA	Yes	Yes	NA	Yes	Yes
De Guzman, Snoswell (9)	Yes	Yes	Yes	Yes	Yes	Yes	Yes	Yes	NA	Yes	Yes
Finkelstein, Knight (10)	Yes	Yes	Yes	Yes	NA	NA	Yes	Yes	NA	Yes	Yes
Gagnon, Ngangue (11)	Yes	Yes	Yes	Yes	NA	NA	Yes	Yes	No	Yes	**Unclear**
Greenhalgh, A'Court and Shaw (12)	Yes	**Unclear**	No	Yes	NA	NA	No	Yes	NA	Yes	Yes
Hassibian and Hassibian (13)	Yes	**Unclear**	No	Yes	NA	NA	**Unclear**	NA	NA	Yes	**Unclear**
Hopstaken, Verweij (14)	Yes	Yes	Yes	Yes	Yes	Yes	Yes	Yes	NA	Yes	Yes
Jacob, Sanchez-Vazquez and Ivory (15)	Yes	Yes	Yes	Yes	Yes	**Unclear**	Yes	Yes	NA	Yes	Yes

For peer review only - http://bmjopen.bmj.com/site/about/guidelines.xhtml

Lewinski, Rushton (16)	Yes	Yes	Yes	Yes	Yes	**Unclear**	Yes	Yes	NA	Yes	Yes
Li, Talaei-Khoei (17)	Yes	Yes	Yes	Yes	**Unclear**	**Unclear**	Yes	Yes	NA	Yes	Yes
Liyanage-Don, Fung (18)	Yes	Yes	Yes	Yes	NA	NA	**Unclear**	Yes	NA	Yes	Yes
Maclure, Stewart and Strath (19)	Yes	Yes	Yes	Yes	Yes	Yes	Yes	Yes	NA	Yes	Yes
Meng, McAiney (20)	Yes	Yes	Yes	Yes	NA	NA	Yes	Yes	NA	Yes	Yes
Metzger, Jatana (21)	Yes	Yes	Yes	Yes	NA	NA	Yes	Yes	NA	Yes	Yes
Mileski, Kruse (22)	Yes	Yes	Yes	Yes	NA	NA	Yes	Yes	NA	Yes	No
Muir, de Boer (23)	Yes	Yes	Yes	Yes	Yes	Yes	No	Yes	NA	Yes	Yes
O'Cathail, Sivanandan (24)	Yes	Yes	Yes	Yes	NA	NA	Yes	Yes	NA	Yes	Yes
Palacholla, Fischer (25)	Yes	Yes	Yes	Yes	NA	NA	Yes	Yes	NA	Yes	Yes
Slater, Campbell (26)	Yes	Yes	Yes	Yes	Yes	Yes	Yes	Yes	NA	Yes	Yes
Smith, Jacobsen (27)	Yes	Yes	Yes	Yes	Yes	Yes	Yes	Yes	NA	Yes	Yes
Varsi, Solberg Nes (28)	Yes	Yes	Yes	Yes	Yes	No	Yes	Yes	NA	Yes	Yes
Vedel, Akhlaghpour (29)	Yes	Yes	Yes	Yes	Yes	Yes	Yes	Yes	**Unclear**	Yes	Yes
Vyas, Hambrick (30)	Yes	Yes	Yes	No	NA	NA	Yes	NA	NA	Yes	Yes
Whitelaw, Pellegrini (31)	Yes	Yes	Yes	Yes	NA	NA	Yes	Yes	NA	Yes	Yes
Xyrichis, Iliopoulou (32)	Yes	Yes	Yes	Yes	Yes	Yes	Yes	Yes	NA	Yes	Yes
Zaman, Khan (33)	Yes	Yes	Yes	Yes	NA	NA	Yes	Yes	NA	Yes	Yes

[1] Questions not applicable for other review types. [2] Question interpreted more broadly in the assessment of other review types: is the synthesis appropriate for the review question? [*] *Not applicable*

For peer review only

For peer review only - http://bmjopen.bmj.com/site/about/guidelines.xhtml

References

1. Almathami HKY, Win KT, Vlahu-Gjorgievska E. Barriers and Facilitators That Influence Telemedicine-Based, Real-Time, Online Consultation at Patients' Homes: Systematic Literature Review. J Med Internet Res. 2020;22(2):e16407.
2. Antoun J. Electronic mail communication between physicians and patients: a review of challenges and opportunities. Fam Pract. 2016;33(2):121-6.
3. Appleton R, Williams J, San Juan NV, Needle JJ, Schlief M, Jordan H, et al. Implementation, adoption, and perceptions of telemental health during the COVID-19 pandemic: systematic review. Journal of medical Internet research. 2021;23(12):e31746.
4. Batterham PJ, Sunderland M, Calear AL, Davey CG, Christensen H, Teesson M, et al. Developing a roadmap for the translation of e-mental health services for depression. Aust N Z J Psychiatry. 2015;49(9):776-84.
5. Bokolo AJ. Exploring the adoption of telemedicine and virtual software for care of outpatients during and after COVID-19 pandemic. Ir J Med Sci. 2021;190(1):1-10.
6. Brunton L, Bower P, Sanders C. The Contradictions of Telehealth User Experience in Chronic Obstructive Pulmonary Disease (COPD): A Qualitative Meta-Synthesis. PLoS One. 2015;10(10):e0139561.
7. Cowan KE, McKean AJ, Gentry MT, Hilty DM. Barriers to Use of Telepsychiatry: Clinicians as Gatekeepers. Mayo Clinic Proceedings. 2019;94(12):2510-23.
8. de Grood C, Raissi A, Kwon Y, Santana MJ. Adoption of e-health technology by physicians: a scoping review. J Multidiscip Healthc. 2016;9:335-44.
9. De Guzman KR, Snoswell CL, Taylor ML, Senanayake B, Haydon HM, Batch JA, et al. A Systematic Review of Pediatric Telediabetes Service Models. Diabetes Technol Ther. 2020;22(8):623-38.
10. Finkelstein J, Knight A, Marinopoulos S, Gibbons MC, Berger Z, Aboumatar H, et al. Enabling patient-centered care through health information technology. Evid Rep Technol Assess (Full Rep). 2012(206):1-1531.
11. Gagnon MP, Ngangue P, Payne-Gagnon J, Desmartis M. m-Health adoption by healthcare professionals: a systematic review. Journal of the American Medical Informatics Association. 2016;23(1):212-20.
12. Greenhalgh T, A'Court C, Shaw S. Understanding heart failure; explaining telehealth - a hermeneutic systematic review. BMC Cardiovasc Disord. 2017;17(1):156.
13. Hassibian MR, Hassibian S. Telemedicine acceptance and implementation in developing countries: Benefits, categories, and barriers. Razavi International Journal of Medicine. 2016;4(3).
14. Hopstaken JS, Verweij L, van Laarhoven CJ, Blijlevens NM, Stommel MW, Hermens RP. Effect of digital care platforms on quality of care for oncological patients and barriers and facilitators for their implementation: Systematic review. Journal of Medical Internet Research. 2021;23(9):e28869.
15. Jacob C, Sanchez-Vazquez A, Ivory C. Social, Organizational, and Technological Factors Impacting Clinicians' Adoption of Mobile Health Tools: Systematic Literature Review. Jmir Mhealth and Uhealth. 2020;8(2).
16. Lewinski AA, Rushton S, Van Voorhees E, Boggan JC, Whited JD, Shoup JP, et al. Implementing remote triage in large health systems: A qualitative evidence synthesis. Res Nurs Health. 2021;44(1):138-54.
17. Li J, Talaei-Khoei A, Seale H, Ray P, Macintyre CR. Health Care Provider Adoption of eHealth: Systematic Literature Review. Interact J Med Res. 2013;2(1):e7.
18. Liyanage-Don N, Fung D, Phillips E, Kronish IM. Implementing Home Blood Pressure Monitoring into Clinical Practice. Current Hypertension Reports. 2019;21(2).
19. Maclure K, Stewart D, Strath A. A systematic review of medical and non-medical practitioners' views of the impact of ehealth on shared care. European Journal of Hospital Pharmacy. 2014;21(1):54-62.

For peer review only - http://bmjopen.bmj.com/site/about/guidelines.xhtml

20. Meng G, McAiney C, Perlman CM, McKillop I, Tisseverasinghe T, Chen HH. Service process factors affecting patients' and clinicians' experiences on rapid teleconsultation implementation in out-patient neurology services during COVID-19 pandemic: a scoping review. BMC health services research. 2022;22(1):1-17.
21. Metzger G, Jatana K, Apfeld J, Deans KJ, Minneci PC, Halaweish I. State of telemedicine use in pediatric surgery in the USA-where we stand and what we can gain from the COVID-19 pandemic: A scoping review. World Journal of Pediatric Surgery. 2021;4(1).
22. Mileski M, Kruse CS, Catalani J, Haderer T. Adopting Telemedicine for the Self-Management of Hypertension: Systematic Review. JMIR Med Inform. 2017;5(4):e41.
23. Muir SD, de Boer K, Nedeljkovic M, Meyer D. Barriers and facilitators of videoconferencing psychotherapy implementation in veteran mental health care environments: a systematic review. BMC Health Serv Res. 2020;20(1):999.
24. O'Cathail M, Sivanandan MA, Diver C, Patel P, Christian J. The Use of Patient-Facing Teleconsultations in the National Health Service: Scoping Review. JMIR Med Inform. 2020;8(3):e15380.
25. Palacholla RS, Fischer N, Coleman A, Agboola S, Kirley K, Felsted J, et al. Provider- and Patient-Related Barriers to and Facilitators of Digital Health Technology Adoption for Hypertension Management: Scoping Review. JMIR Cardio. 2019;3(1):e11951.
26. Slater H, Campbell JM, Stinson JN, Burley MM, Briggs AM. End User and Implementer Experiences of mHealth Technologies for Noncommunicable Chronic Disease Management in Young Adults: Systematic Review. J Med Internet Res. 2017;19(12):e406.
27. Smith SM, Jacobsen JHW, Atlas AP, Khoja A, Kovoor JG, Tivey DR, et al. Telehealth in surgery: an umbrella review. ANZ journal of surgery. 2021;91(11):2360-75.
28. Varsi C, Solberg Nes L, Kristjansdottir OB, Kelders SM, Stenberg U, Zangi HA, et al. Implementation Strategies to Enhance the Implementation of eHealth Programs for Patients With Chronic Illnesses: Realist Systematic Review. J Med Internet Res. 2019;21(9):e14255.
29. Vedel I, Akhlaghpour S, Vaghefi I, Bergman H, Lapointe L. Health information technologies in geriatrics and gerontology: a mixed systematic review. Journal of the American Medical Informatics Association. 2013;20(6):1109-19.
30. Vyas KS, Hambrick HR, Shakir A, Morrison SD, Tran DC, Pearson K, et al. A Systematic Review of the Use of Telemedicine in Plastic and Reconstructive Surgery and Dermatology. Ann Plast Surg. 2017;78(6):736-68.
31. Whitelaw S, Pellegrini DM, Mamas MA, Cowie M, Van Spall HGC. Barriers and facilitators of the uptake of digital health technology in cardiovascular care: a systematic scoping review. Eur Heart J Digit Health. 2021;2(1):62-74.
32. Xyrichis A, Iliopoulou K, Mackintosh NJ, Bench S, Terblanche M, Philippou J, Sandall J. Healthcare stakeholders' perceptions and experiences of factors affecting the implementation of critical care telemedicine (CCT): qualitative evidence synthesis. Cochrane Database Syst Rev. 2021;2(2):Cd012876.
33. Zaman SB, Khan RK, Evans RG, Thrift AG, Maddison R, Islam SMS. Exploring Barriers to and Enablers of the Adoption of Information and Communication Technology for the Care of Older Adults With Chronic Diseases: Scoping Review. JMIR aging. 2022;5(1):e25251.

For peer review only - http://bmjopen.bmj.com/site/about/guidelines.xhtml

Reporting checklist for systematic review (with or without a meta-analysis).

Based on the PRISMA guidelines.

Instructions to authors

Complete this checklist by entering the page numbers from your manuscript where readers will find each of the items listed below.

Your article may not currently address all the items on the checklist. Please modify your text to include the missing information. If you are certain that an item does not apply, please write "n/a" and provide a short explanation.

Upload your completed checklist as an extra file when you submit to a journal.

In your methods section, say that you used the PRISMAreporting guidelines, and cite them as:

Page MJ, McKenzie JE, Bossuyt PM, Boutron I, Hoffmann TC, Mulrow CD, Shamseer L, Tetzlaff JM, Akl EA, Brennan SE, Chou R, Glanville J, Grimshaw JM, Hróbjartsson A, Lalu MM, Li T, Loder EW, Mayo-Wilson E, McDonald S, McGuinness LA, Stewart LA, Thomas J, Tricco AC, Welch VA, Whiting P, Moher D. The PRISMA 2020 statement: An updated guideline for reporting systematic reviews

		Reporting Item	Page Number
**Title**			
Title	#1	Identify the report as a systematic review	1
**Abstract**			

For peer review only

Abstract	#2	Report an abstract addressing each item in the PRISMA 2020 for Abstracts checklist	2
**Introduction**			
Background/rationale	#3	Describe the rationale for the review in the context of existing knowledge	5
Objectives	#4	Provide an explicit statement of the objective(s) or question(s) the review addresses	5
**Methods**			
Eligibility criteria	#5	Specify the inclusion and exclusion criteria for the review and how studies were grouped for the syntheses	7
Information sources	#6	Specify all databases, registers, websites, organisations, reference lists, and other sources searched or consulted to identify studies. Specify the date when each source was last searched or consulted	8
Search strategy	#7	Present the full search strategies for all databases, registers, and websites, including any filters and limits used	8
Selection process	#8	Specify the methods used to decide whether a study met the inclusion criteria of the review, including how many reviewers screened each record and each report retrieved, whether they worked independently, and, if applicable, details of automation tools used in the process	9

For peer review only

Data collection process	#9	Specify the methods used to collect data from reports, including how many reviewers collected data from each report, whether they worked independently, any processes for obtaining or confirming data from study investigators, and, if applicable, details of automation tools used in the process	9
Data items	#10a	List and define all outcomes for which data were sought. Specify whether all results that were compatible with each outcome domain in each study were sought (for example, for all measures, time points, analyses), and, if not, the methods used to decide which results to collect	9
Study risk of bias assessment	#11	Specify the methods used to assess risk of bias in the included studies, including details of the tool(s) used, how many reviewers assessed each study and whether they worked independently, and, if applicable, details of automation tools used in the process	10
Effect measures	#12	Specify for each outcome the effect measure(s) (such as risk ratio, mean difference) used in the synthesis or presentation of results	NA
Synthesis methods	#13a	Describe the processes used to decide which studies were eligible for each synthesis (such as tabulating the study intervention characteristics and comparing against the planned groups for each synthesis (item #5))	9-10

For peer review only

Synthesis methods	#13b	Describe any methods required to prepare the data for presentation or synthesis, such as handling of missing summary statistics or data conversions	9-10
Synthesis methods	#13c	Describe any methods used to tabulate or visually display results of individual studies and syntheses	9-10
Synthesis methods	#13d	Describe any methods used to synthesise results and provide a rationale for the choice(s). If meta-analysis was performed, describe the model(s), method(s) to identify the presence and extent of statistical heterogeneity, and software package(s) used	9-10
Synthesis methods	#13e	Describe any methods used to explore possible causes of heterogeneity among study results (such as subgroup analysis, meta-regression)	NA
Synthesis methods	#13f	Describe any sensitivity analyses conducted to assess robustness of the synthesised results	NA
Reporting bias assessment	#14	Describe any methods used to assess risk of bias due to missing results in a synthesis (arising from reporting biases)	NA
Certainty assessment	#15	Describe any methods used to assess certainty (or confidence) in the body of evidence for an outcome	NA
Data items	#10b	List and define all other variables for which data were sought (such as participant and intervention characteristics, funding sources). Describe any	9

For peer review only

		assumptions made about any missing or unclear information	
**Results**			
Study selection	#16a	Describe the results of the search and selection process, from the number of records identified in the search to the number of studies included in the review, ideally using a flow diagram (http://www.prisma-statement.org/PRISMAStatement/FlowDiagram)	11
Study selection	#16b	Cite studies that might appear to meet the inclusion criteria, but which were excluded, and explain why they were excluded	11
Study characteristics	#17	Cite each included study and present its characteristics	14
Risk of bias in studies	#18	Present assessments of risk of bias for each included study	NA
Results of individual studies	#19	For all outcomes, present for each study (a) summary statistics for each group (where appropriate) and (b) an effect estimate and its precision (such as confidence/credible interval), ideally using structured tables or plots	NA
Results of syntheses	#20a	For each synthesis, briefly summarise the characteristics and risk of bias among contributing studies	NA
Results of syntheses	#20b	Present results of all statistical syntheses conducted. If meta-analysis was done, present for each the summary	NA

For peer review only

		estimate and its precision (such as confidence/credible interval) and measures of statistical heterogeneity. If comparing groups, describe the direction of the effect	
Results of syntheses	#20c	Present results of all investigations of possible causes of heterogeneity among study results	NA
Results of syntheses	#20d	Present results of all sensitivity analyses conducted to assess the robustness of the synthesised results	NA
Risk of reporting biases in syntheses	#21	Present assessments of risk of bias due to missing results (arising from reporting biases) for each synthesis assessed	NA
Certainty of evidence	#22	Present assessments of certainty (or confidence) in the body of evidence for each outcome assessed	NA
**Discussion**			
Results in context	#23a	Provide a general interpretation of the results in the context of other evidence	20
Limitations of included studies	#23b	Discuss any limitations of the evidence included in the review	22
Limitations of the review methods	#23c	Discuss any limitations of the review processes used	22
Implications	#23d	Discuss implications of the results for practice, policy, and future research	23
**Other information**			

For peer review only

Registration and protocol	#24a	Provide registration information for the review, including register name and registration number, or state that the review was not registered	NA
Registration and protocol	#24b	Indicate where the review protocol can be accessed, or state that a protocol was not prepared	n/a
Registration and protocol	#24c	Describe and explain any amendments to information provided at registration or in the protocol	NA
Support	#25	Describe sources of financial or non-financial support for the review, and the role of the funders or sponsors in the review	24
Competing interests	#26	Declare any competing interests of review authors	24
Availability of data, code, and other materials	#27	Report which of the following are publicly available and where they can be found: template data collection forms; data extracted from included studies; data used for all analyses; analytic code; any other materials used in the review	24

The PRISMA checklist is distributed under the terms of the Creative Commons Attribution License CC-BY. This checklist was completed on 19. May 2023 using https://www.goodreports.org/, a tool made by the EQUATOR Network in collaboration with Penelope.ai

For peer review only
